# Supplementary material for: Activation of an atypical plant NLR with an N-terminal deletion initiates cell death at the vacuole
Source: EMBO Rep. 2024 Sep 6;25(10):19. doi: 10.1038/s44319-024-00240-4 (PMC11467418; doi:10.1038/s44319-024-00240-4)

Appendix for

Activation of an atypical plant NLR with an N-terminal deletion initiates cell death at the vacuole

Table of content:

Appendix Figure legends……………………………………………………..page 1

Appendix Figure S1…………………………………………………………..page 2

Appendix Figure S2…………………………………………………………..page 3

Appendix Figure S3…………………………………………………………..page 4

Appendix Figure S4…………………………………………………………..page 5

Appendix Figure S1. Most Arabidopsis ANLs are also PML NLRs.

Arabidopsis Col-0 ANLs can be grouped into 5 subclusters, and most ANLs have a putative N-myristoylation and/or S-acylation site at their N-termini (ANLs lacking this site(s) are indicated by a black star). PML5/At1g61300 is in subclusters 3 with PML11/At1g61180, PML12/At1g61310 and PML13/At1g61190. Phylogenetic rooted tree is based on an alignment build with full length protein sequences and was generated with ClustalW 3.0 tool of CLC Main Workbench v.23.0.Bootstrap values are shown at the branches.

Appendix Figure S2. Amino acid alignment of full-length Col-0 PML5 and its closest homologs.

Alignment of PML5/At1g61300 and its closest Col-0 homologs PML11/At1g61180, PML12/At1g61310 and PML13/At1g61190. Differences in amino acid composition are highlighted in light green and amino acid conservation (in percentage) is presented as a bar-blot in blue. Alignment was done with CLC Main Workbench tool v.22.02.

Appendix Figure S3. Alignment of PML5 and PML5-like NLRs of different Arabidopsis accessions.

Alignment of Col-0 PML5/At1g61300 and PML5-like NLRs of 13 Arabidopsis accessions. The close Col-0 homolog PML11/At1g61180 is shown to better visualize the 113 amino acid deletion. Differences in amino acid composition are highlighted in light green and amino acid conservation (in percentage) is presented as a bar-blot in blue. Alignment was done with CLC Main Workbench tool v.22.02.

Appendix Figure S4. PML5-like deletion may have occurred independently in *Arabidopsis* and *Capsella* species.

A maximum likelihood phylogeny based on the amino acid sequence of the NB-ARC domain of PML paralogs from representatives of the Brassicales. Nodes with black dots have a bootstrap support of at least 85%. The multiple sequence alignment next to the phylogeny shows the presence and absence of the 5-prime deletion across the tree. Arabidopsis PML5 sequences are nested within the intact homologs present in the species.

Appendix Figure S1

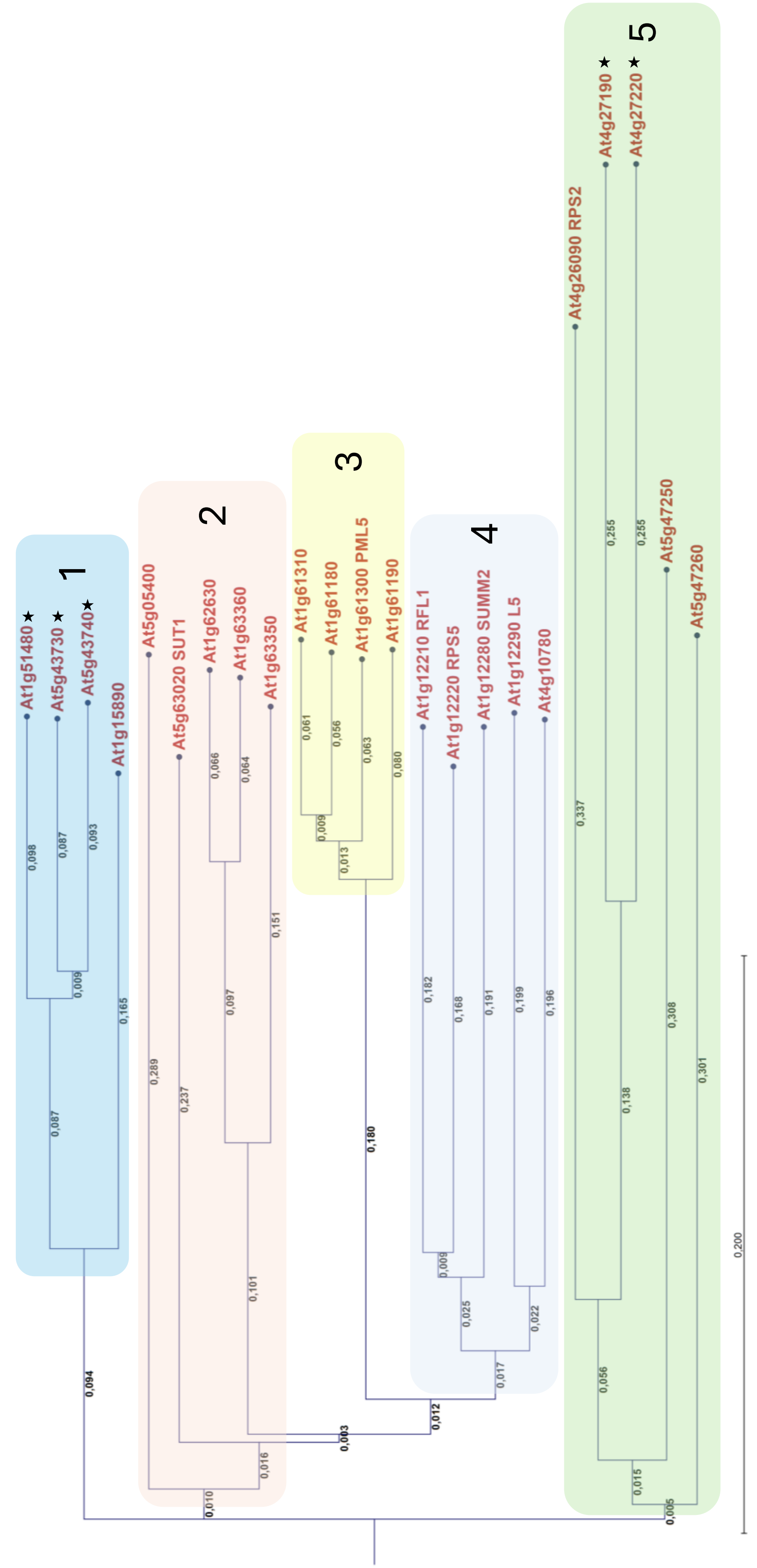

Appendix Figure S2

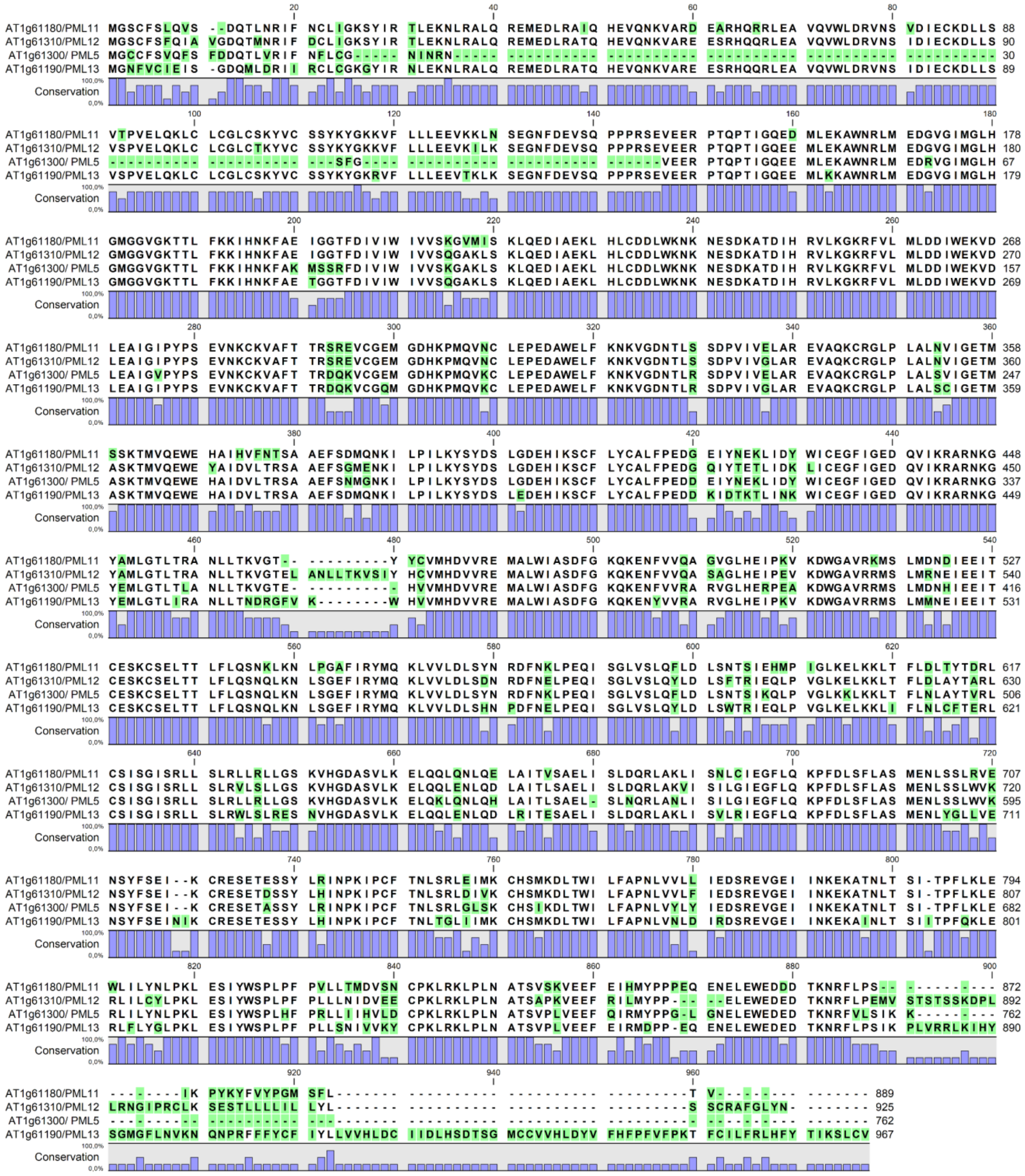

Appendix Figure S3

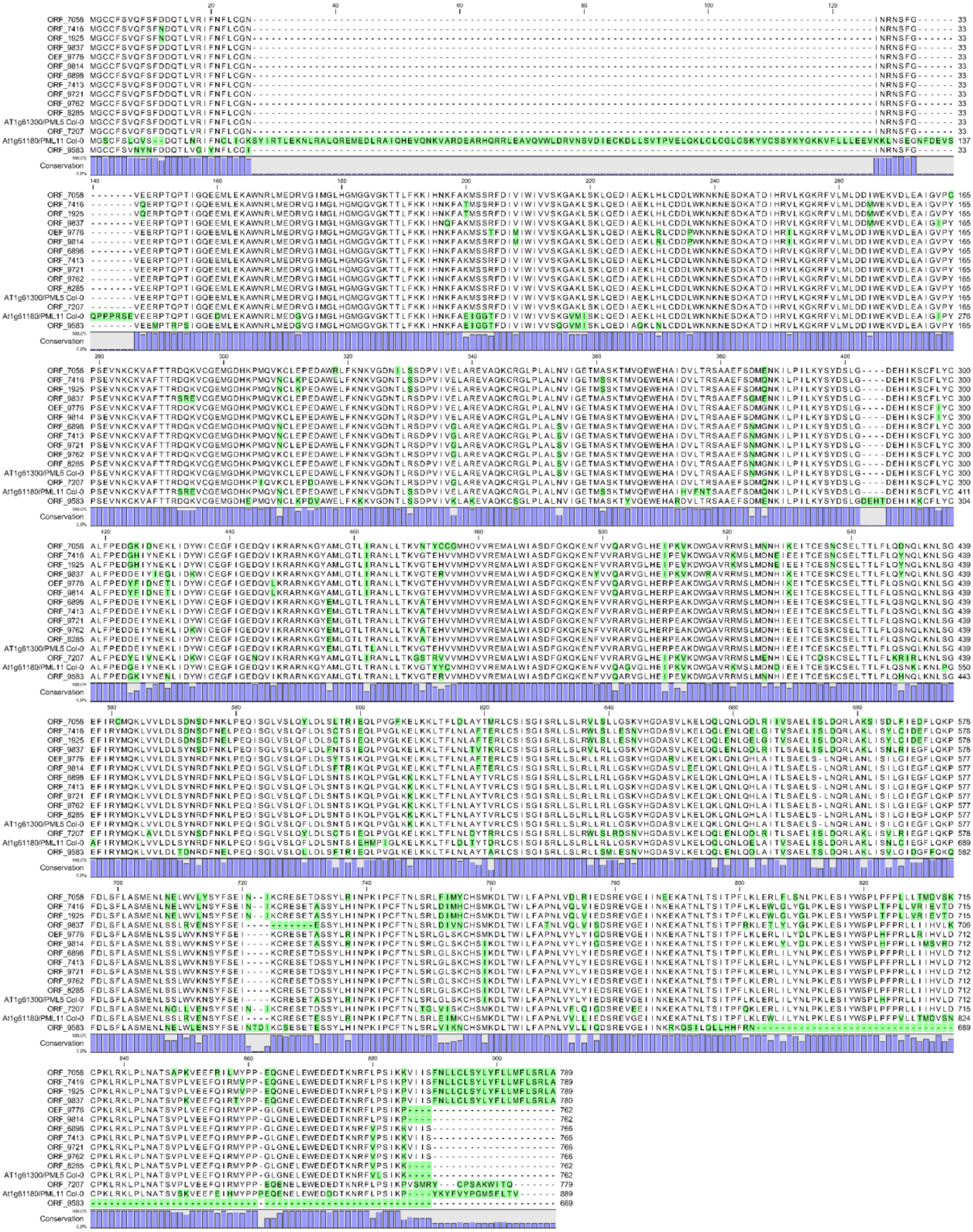

Appendix Figure S4

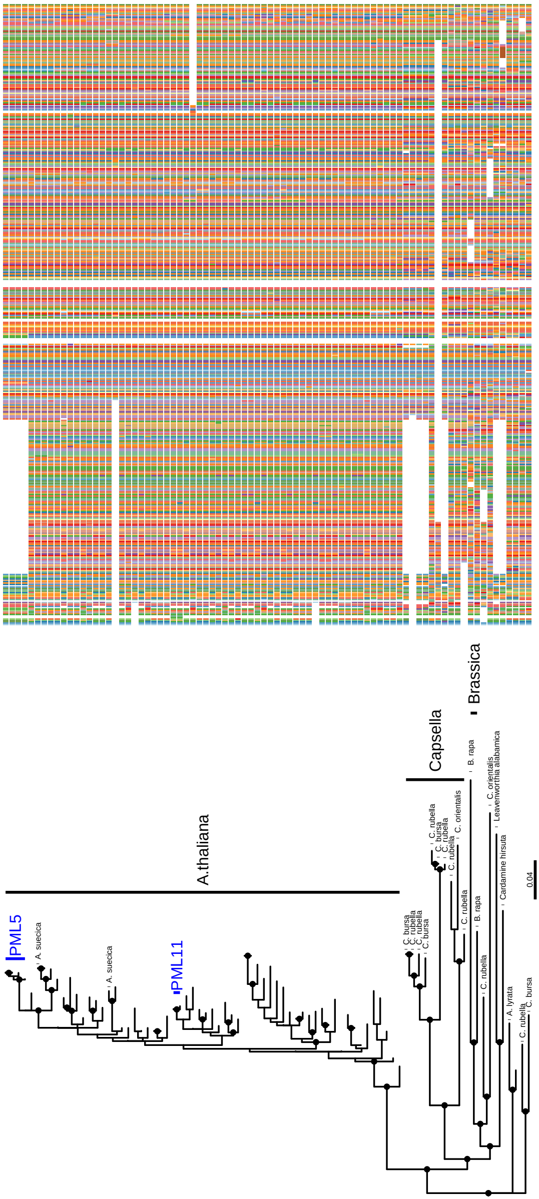

Supplement: Supplementary file 1 — Appendix [file 44319_2024_240_MOESM1_ESM.pdf]
