# Supplementary figures and images for: Activation of an atypical plant NLR with an N-terminal deletion initiates cell death at the vacuole
Source: EMBO Rep. 2024 Sep 6;25(10):19. doi: 10.1038/s44319-024-00240-4 (PMC11467418; doi:10.1038/s44319-024-00240-4)

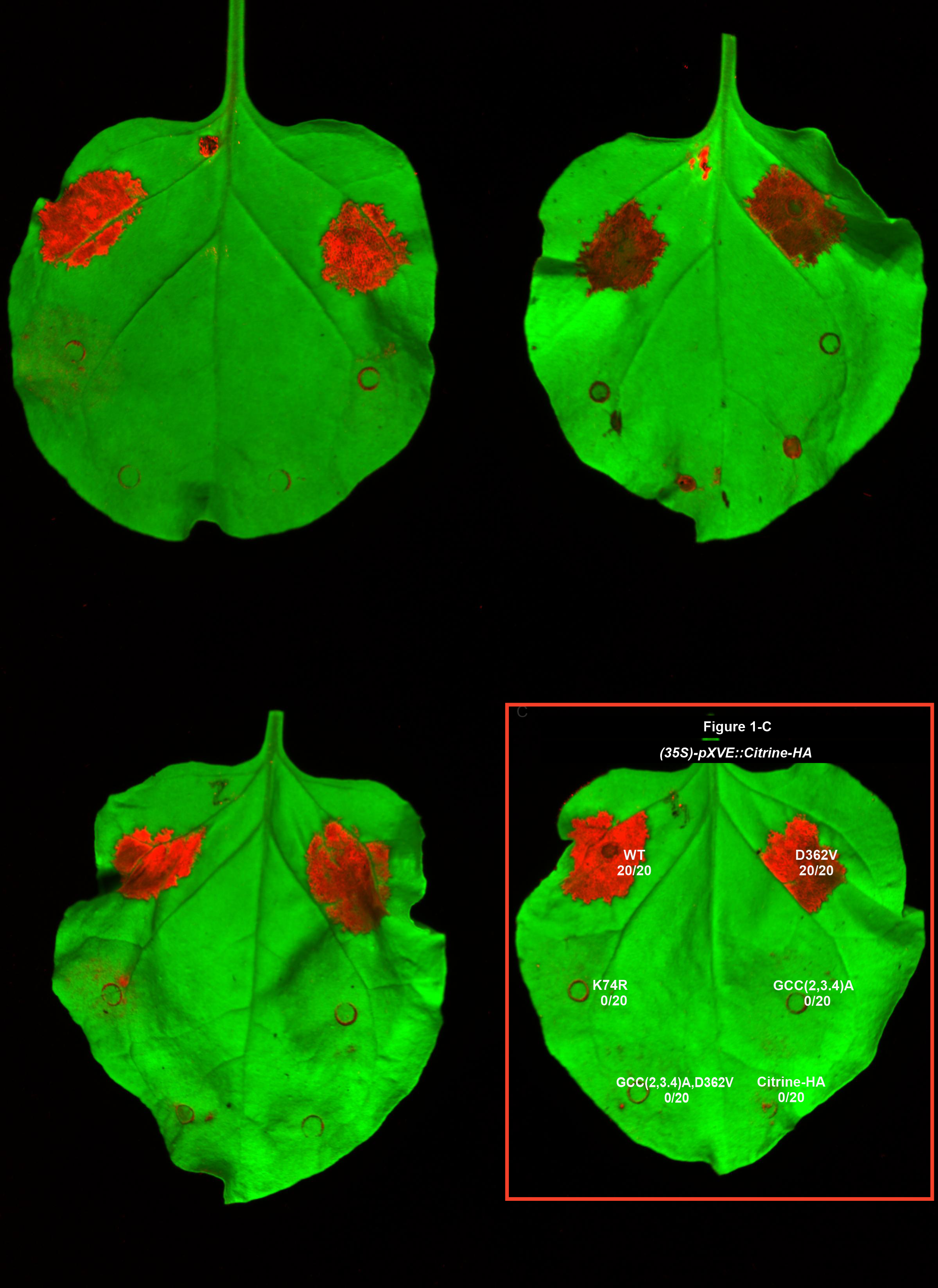

Supplement: Supplementary file 6 — Source data Fig. 1 [file 44319_2024_240_MOESM6_ESM.zip › Figure 1/1C/Figure 1C.1.png]

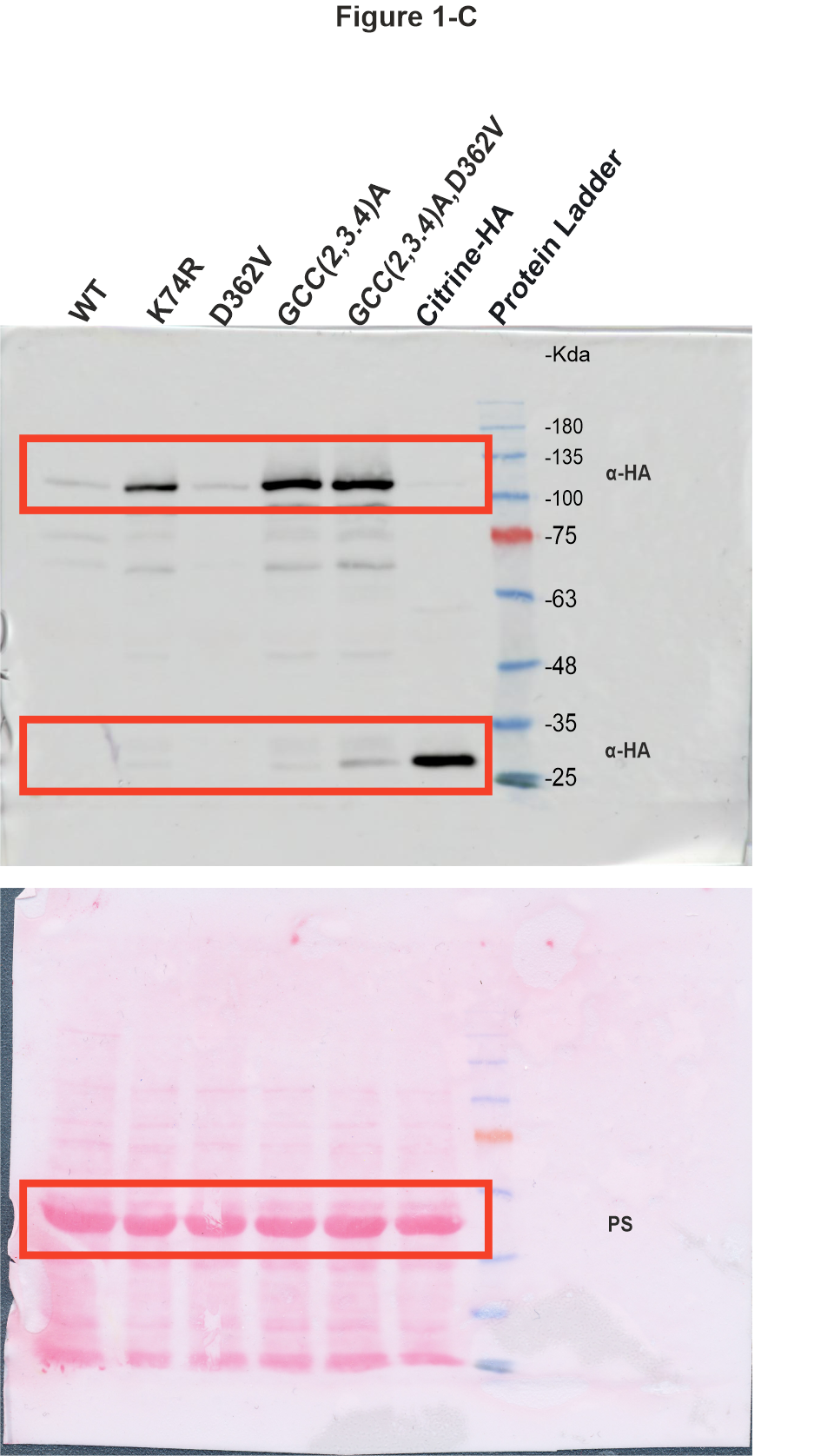

Supplement: Supplementary file 6 — Source data Fig. 1 [file 44319_2024_240_MOESM6_ESM.zip › Figure 1/1C/Figure 1C.2.png]

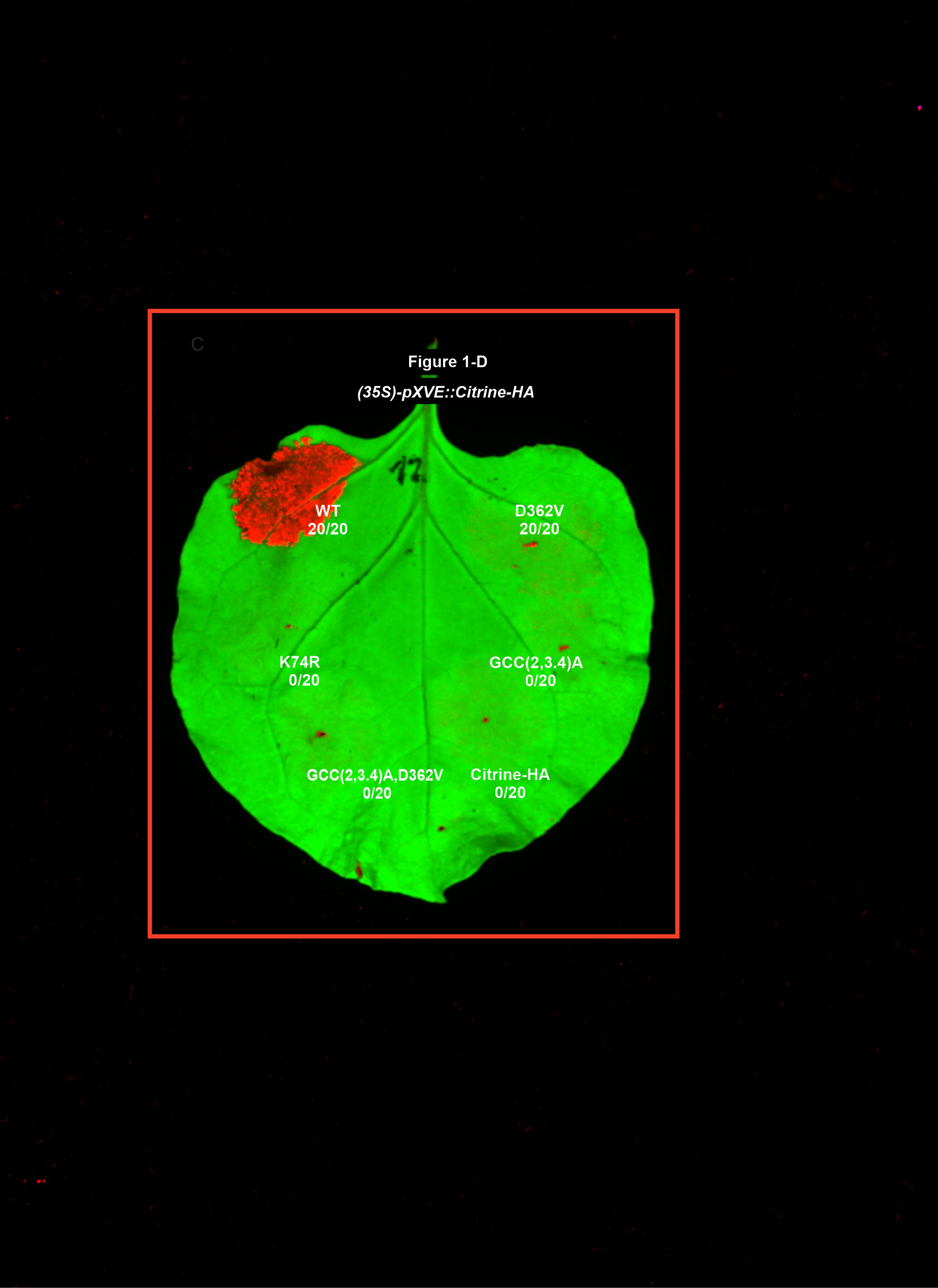

Supplement: Supplementary file 6 — Source data Fig. 1 [file 44319_2024_240_MOESM6_ESM.zip › Figure 1/1D/Figure 1D.1.png]

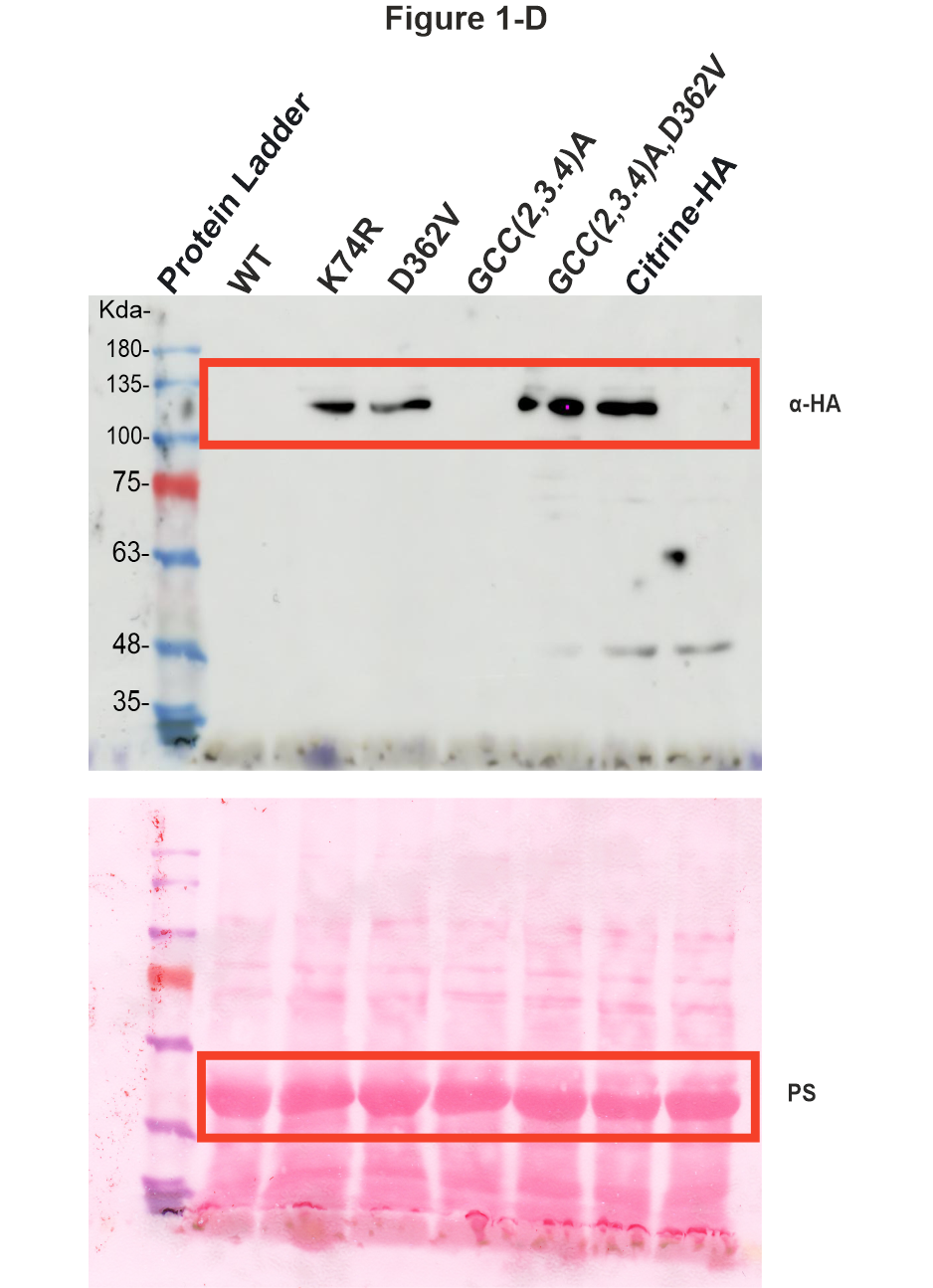

Supplement: Supplementary file 6 — Source data Fig. 1 [file 44319_2024_240_MOESM6_ESM.zip › Figure 1/1D/Figure 1D.2.png]

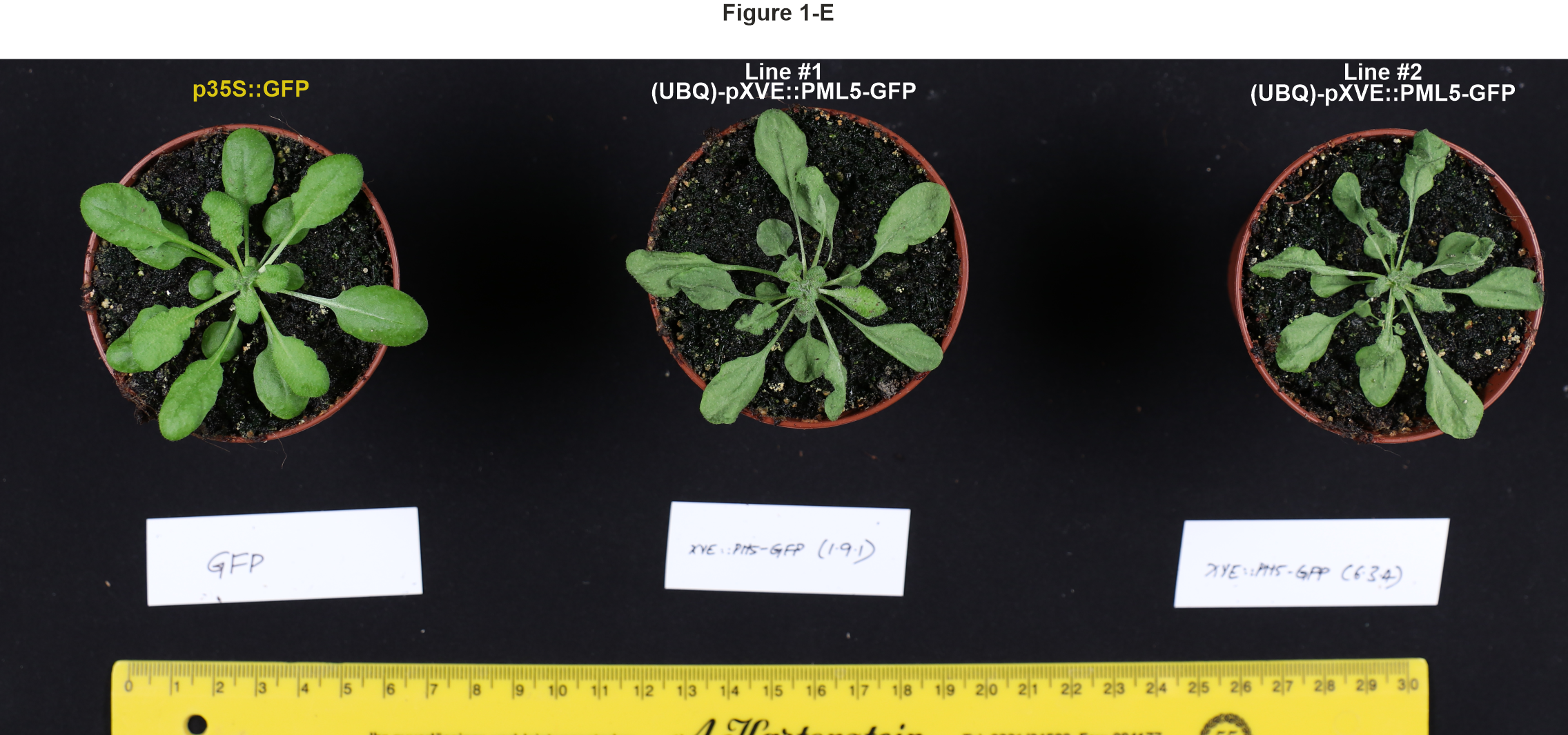

Supplement: Supplementary file 6 — Source data Fig. 1 [file 44319_2024_240_MOESM6_ESM.zip › Figure 1/1E/Figure 1E.png]

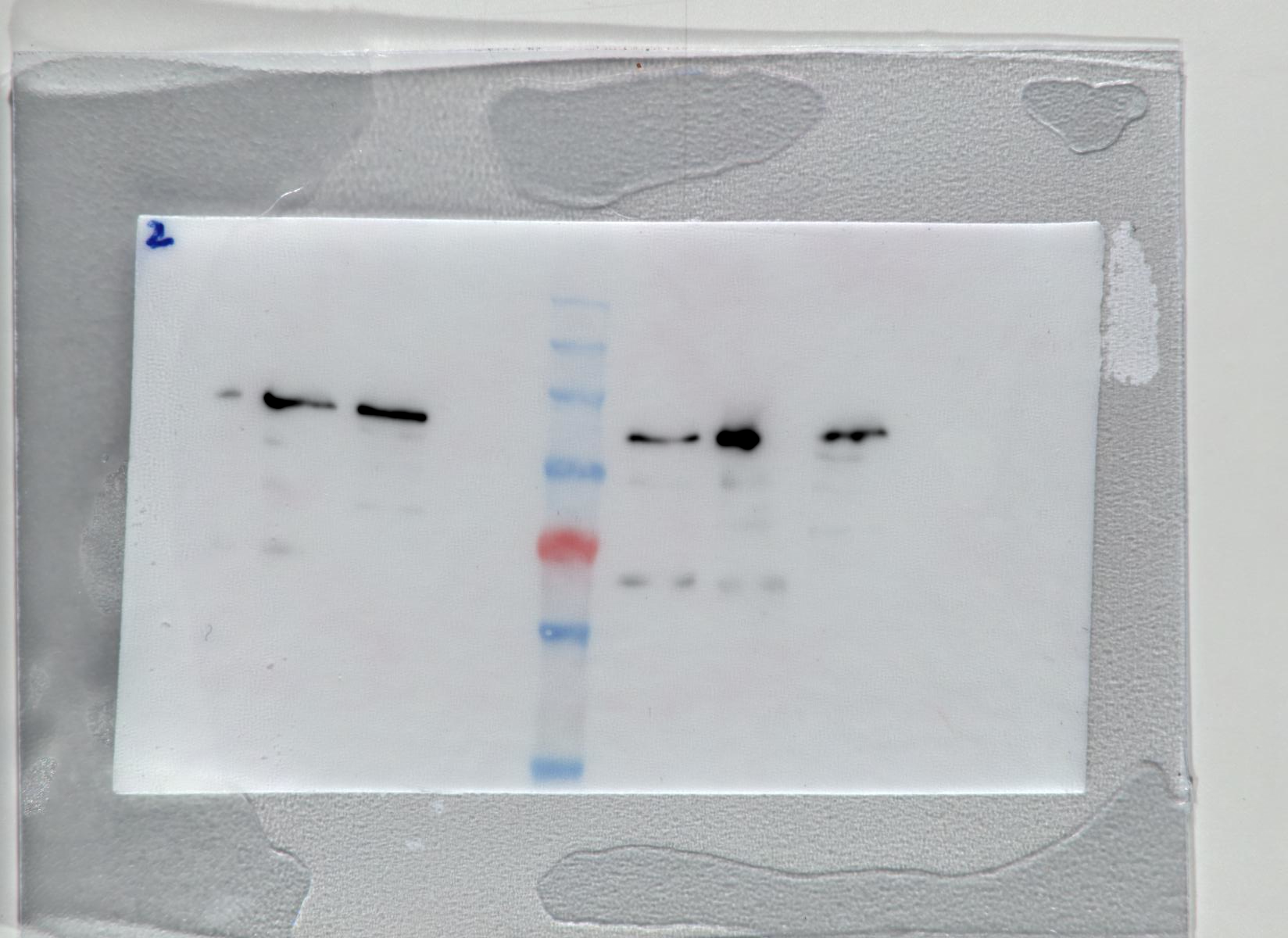

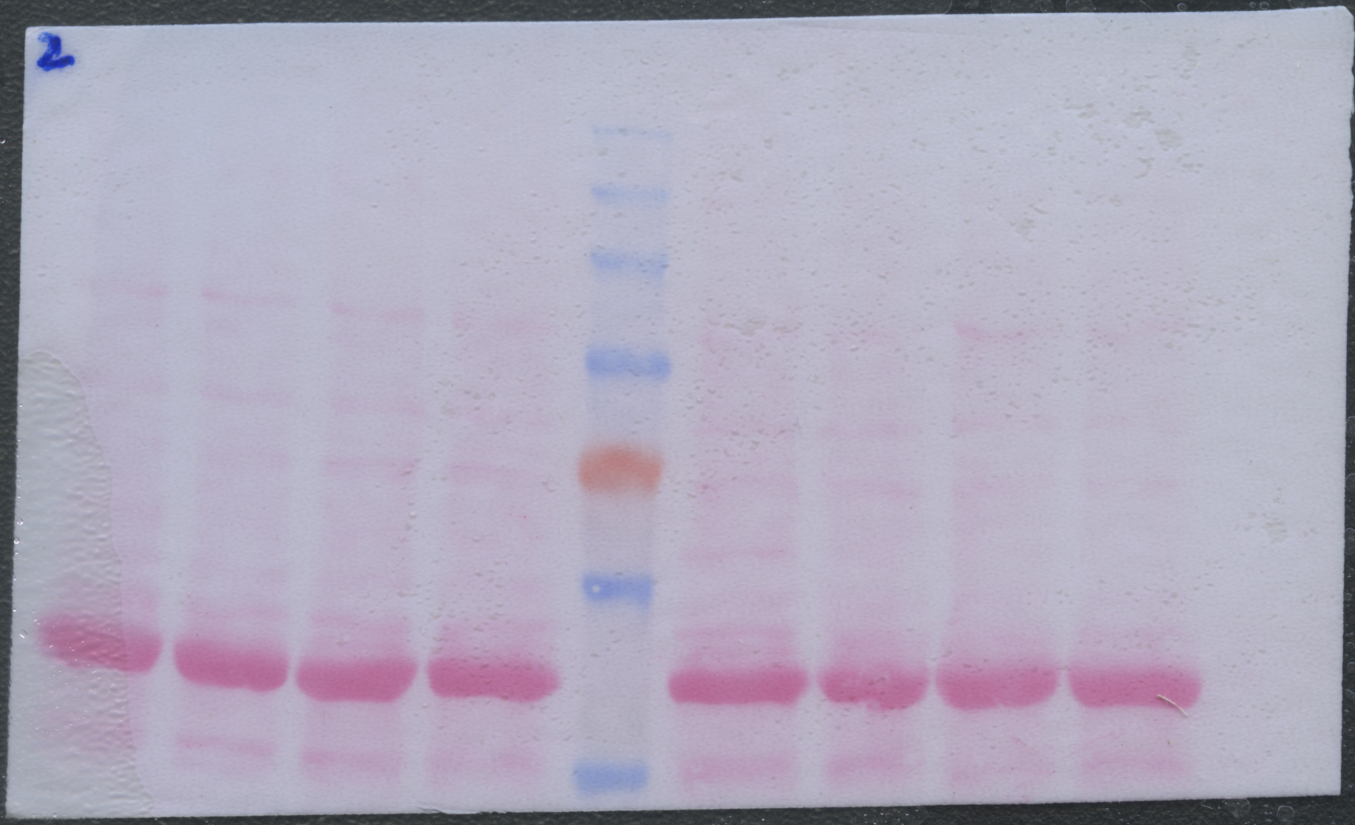

Supplement: Supplementary file 6 — Source data Fig. 1 [file 44319_2024_240_MOESM6_ESM.zip › Figure 1/1F/1F_Calcium Blot and PS.docx]

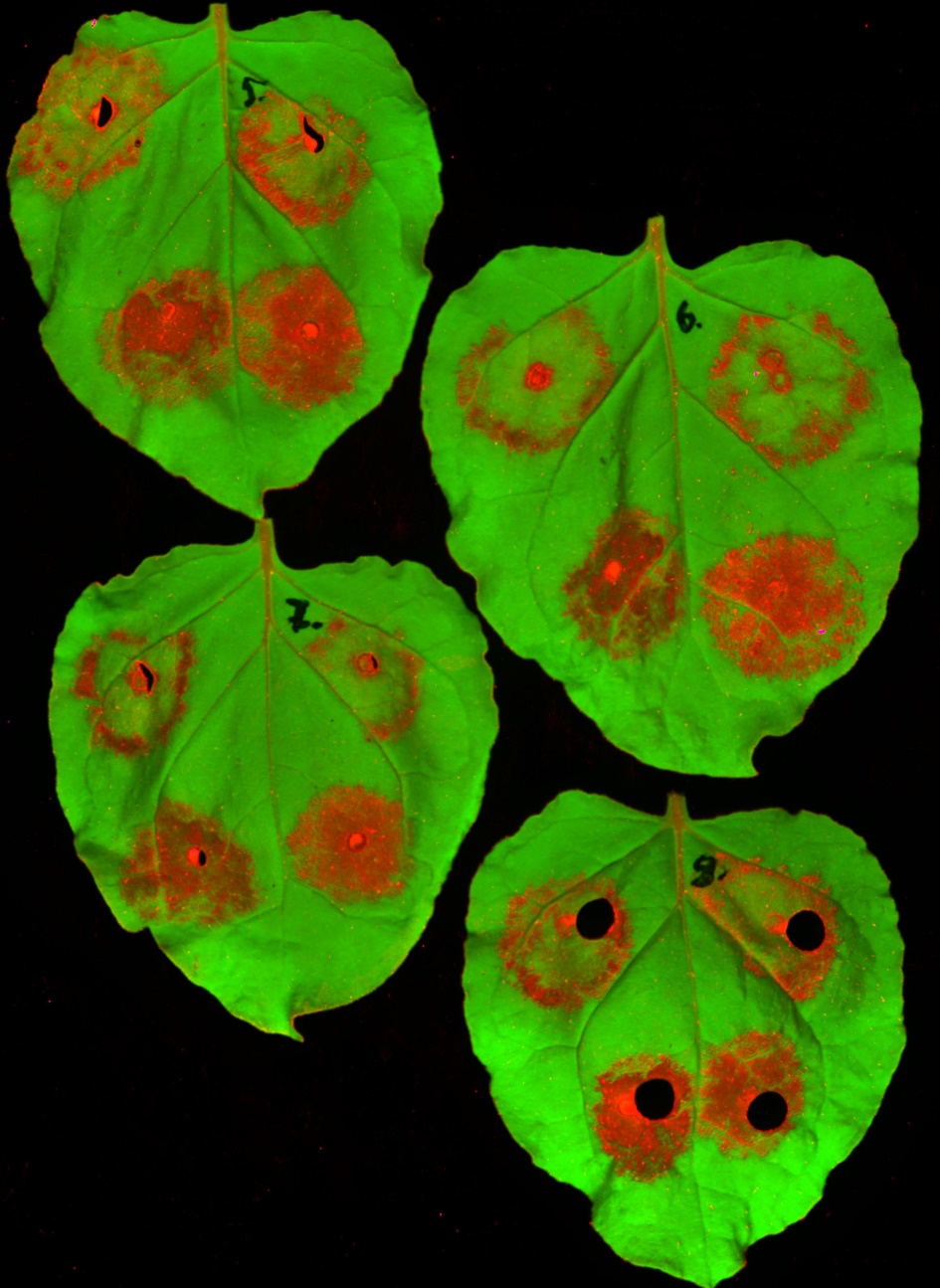

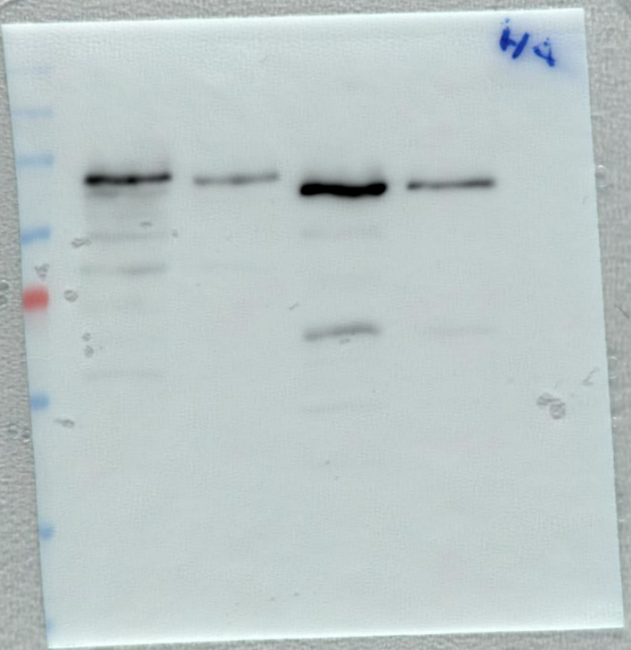


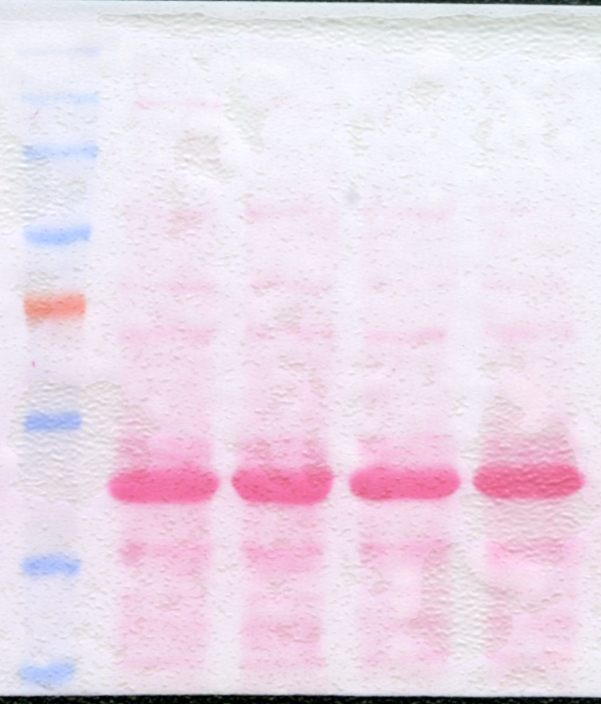

Supplement: Supplementary file 6 — Source data Fig. 1 [file 44319_2024_240_MOESM6_ESM.zip › Figure 1/1G/1F_La_Cell death_WB_PS.docx]

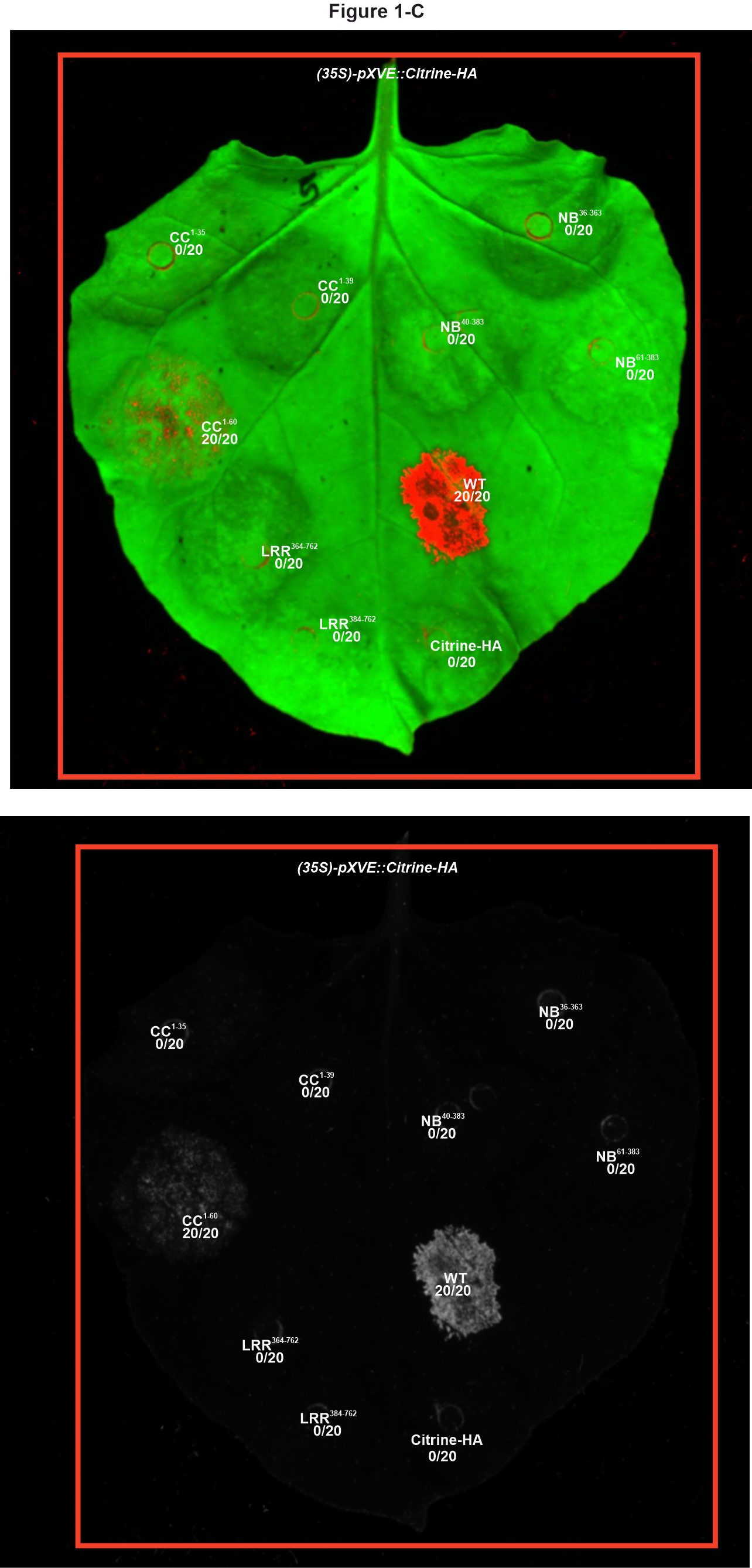

Supplement: Supplementary file 7 — Source data Fig. 2 [file 44319_2024_240_MOESM7_ESM.zip › Figure 2/2B/Fig 2B.1.png]

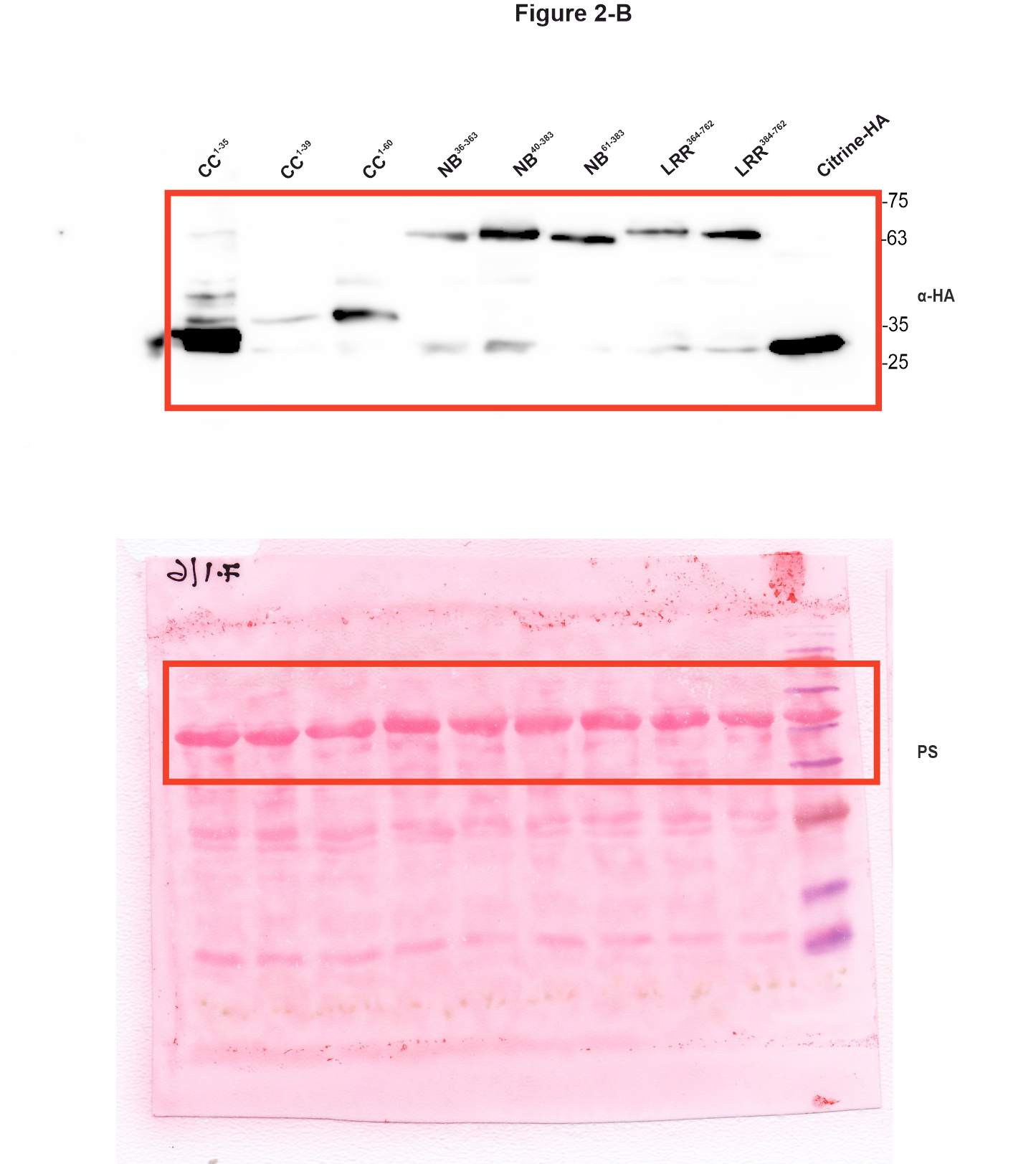

Supplement: Supplementary file 7 — Source data Fig. 2 [file 44319_2024_240_MOESM7_ESM.zip › Figure 2/2B/Fig 2B.2.png]

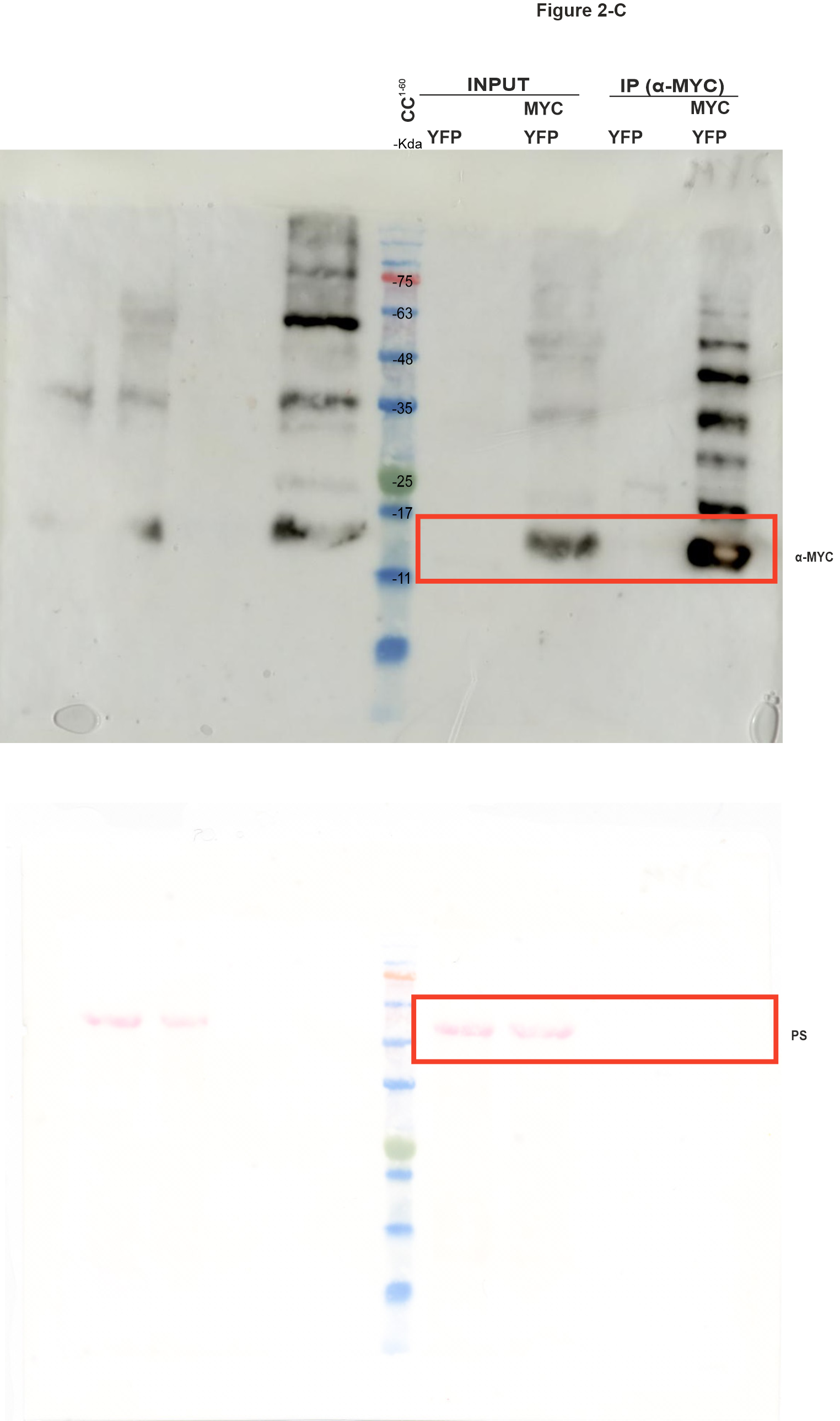

Supplement: Supplementary file 7 — Source data Fig. 2 [file 44319_2024_240_MOESM7_ESM.zip › Figure 2/2C/Fig 2C.1.png]

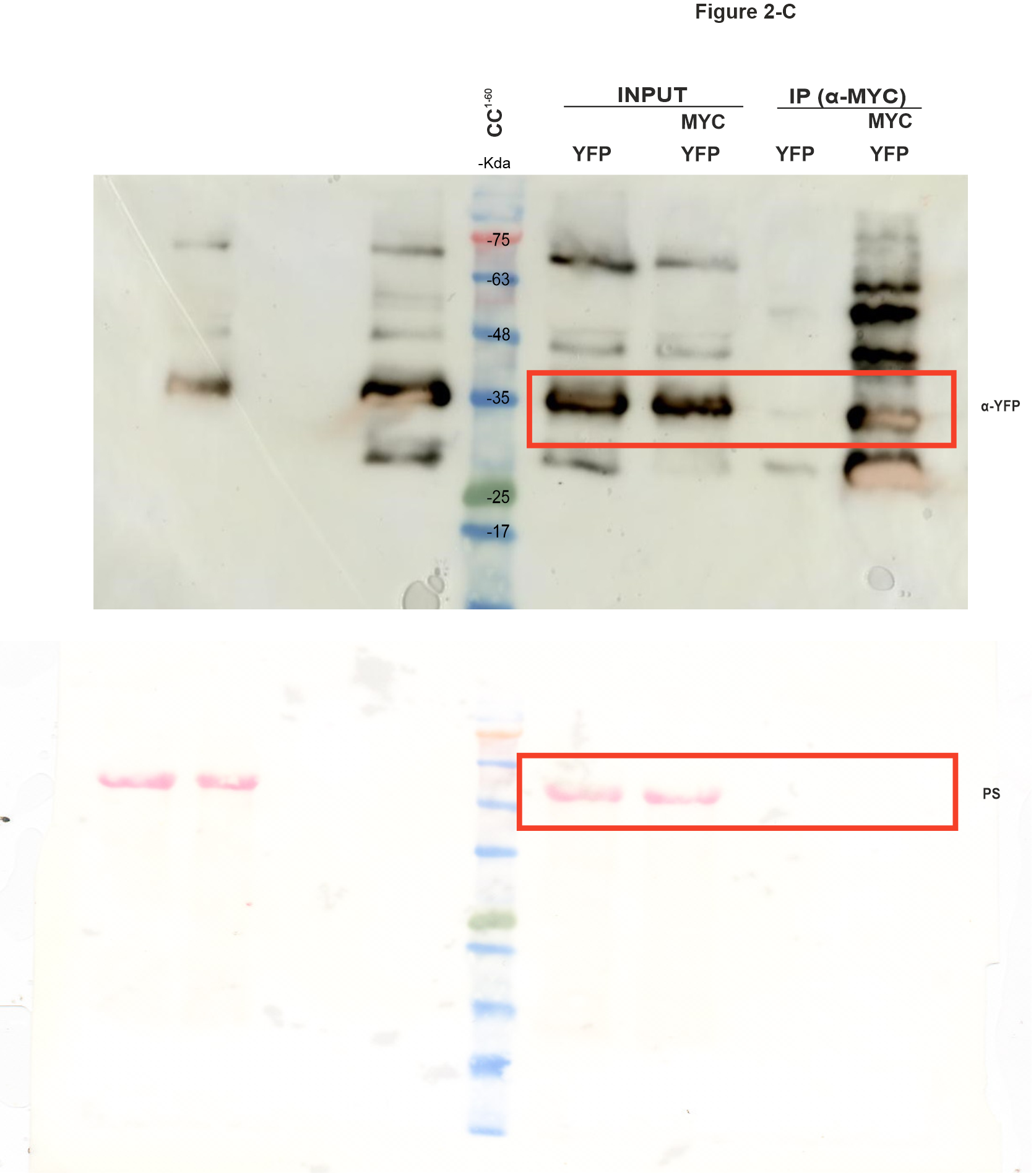

Supplement: Supplementary file 7 — Source data Fig. 2 [file 44319_2024_240_MOESM7_ESM.zip › Figure 2/2C/Fig 2C.2.png]

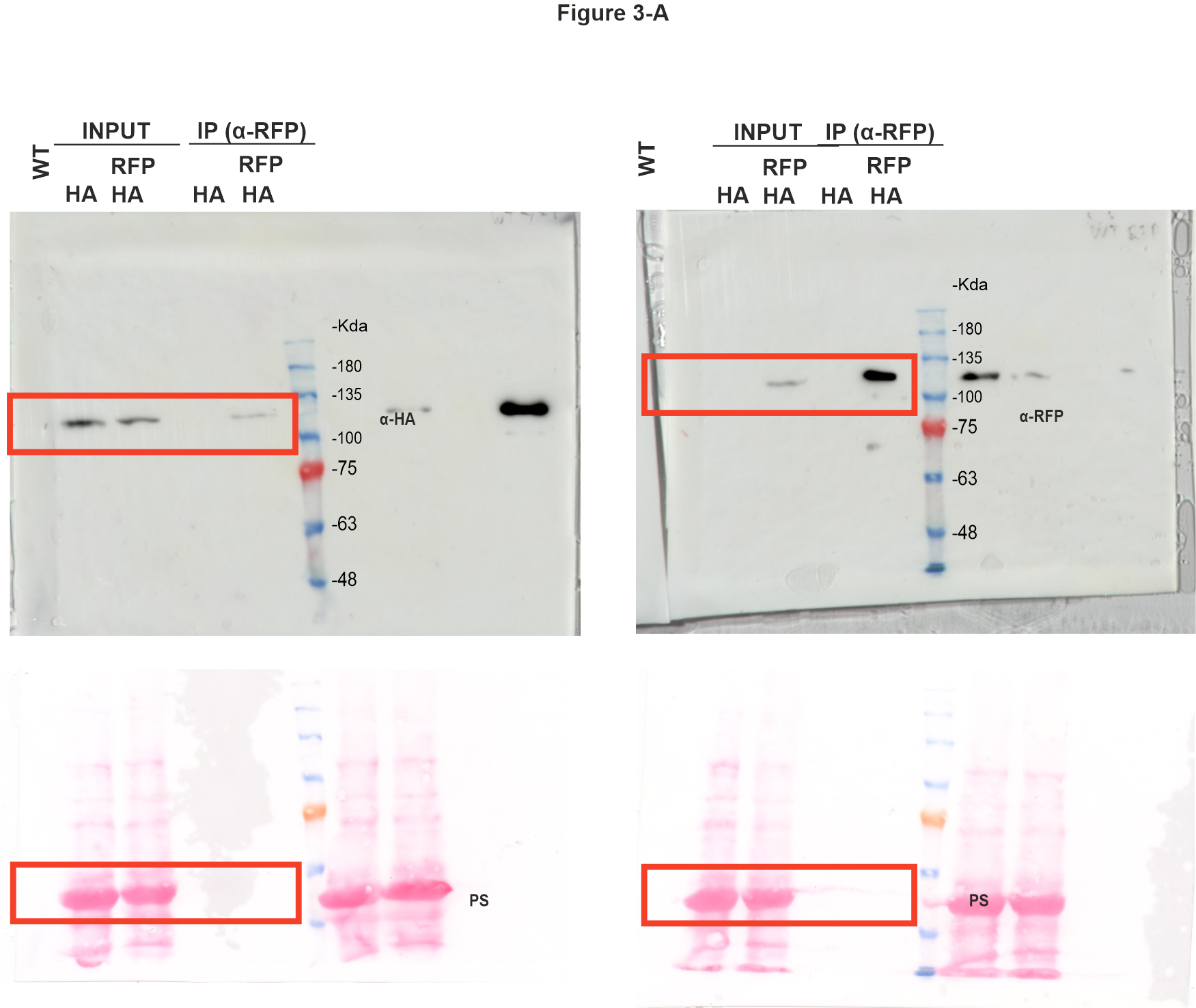

Supplement: Supplementary file 8 — Source data Fig. 3 [file 44319_2024_240_MOESM8_ESM.zip › Figure 3/3A/Fig 3 A.png]

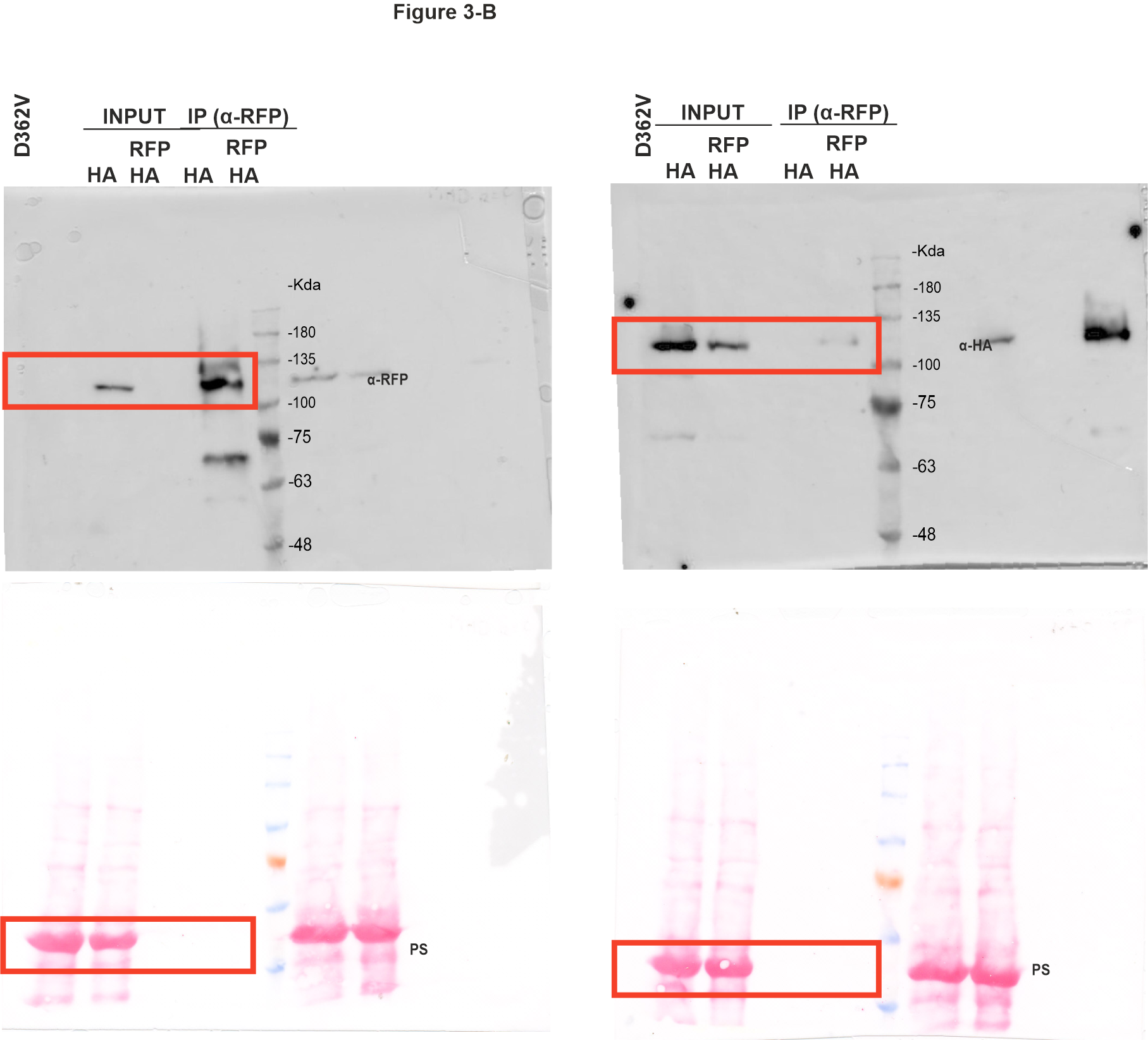

Supplement: Supplementary file 8 — Source data Fig. 3 [file 44319_2024_240_MOESM8_ESM.zip › Figure 3/3B/Fig 3 B.png]

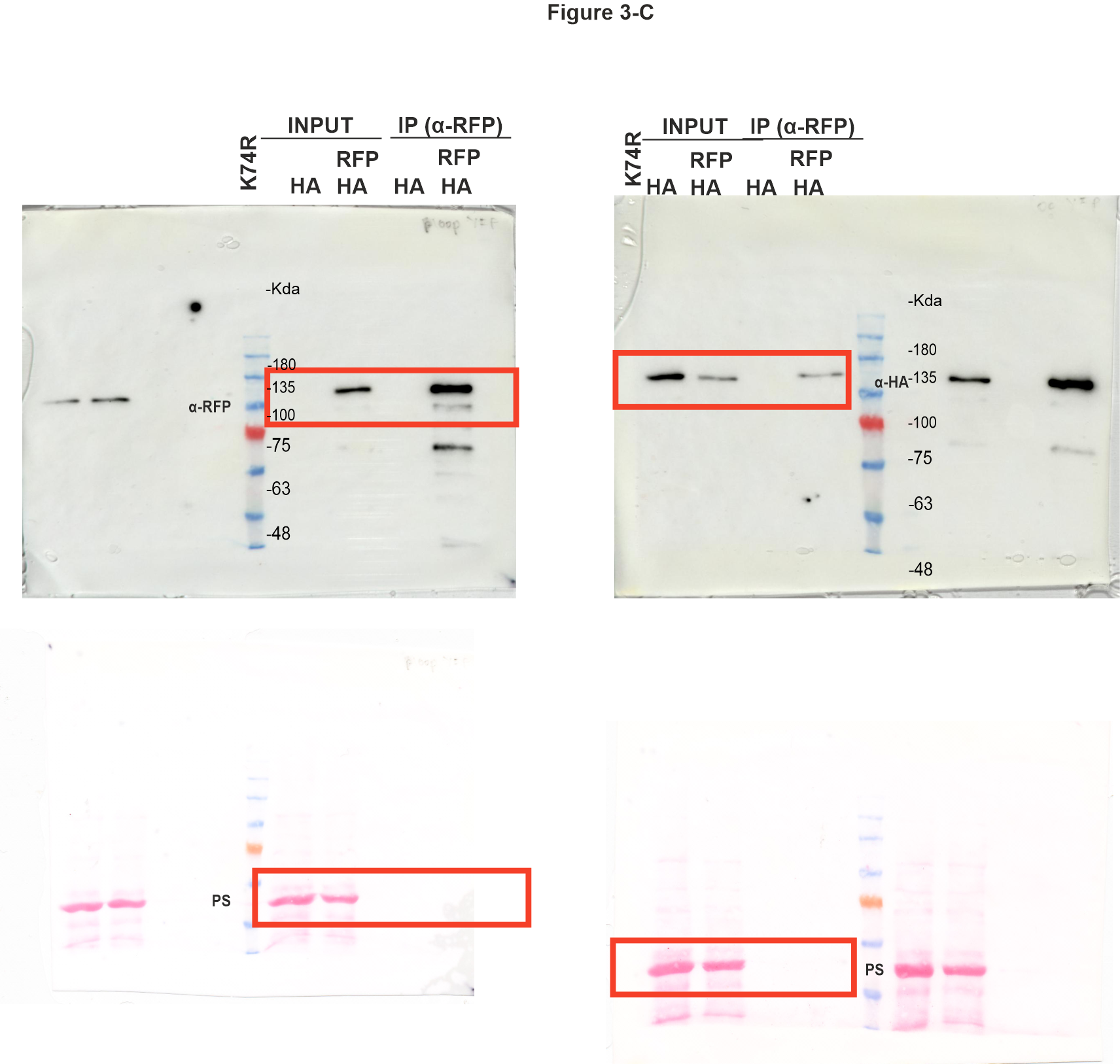

Supplement: Supplementary file 8 — Source data Fig. 3 [file 44319_2024_240_MOESM8_ESM.zip › Figure 3/3C/Fig 3 C.png]

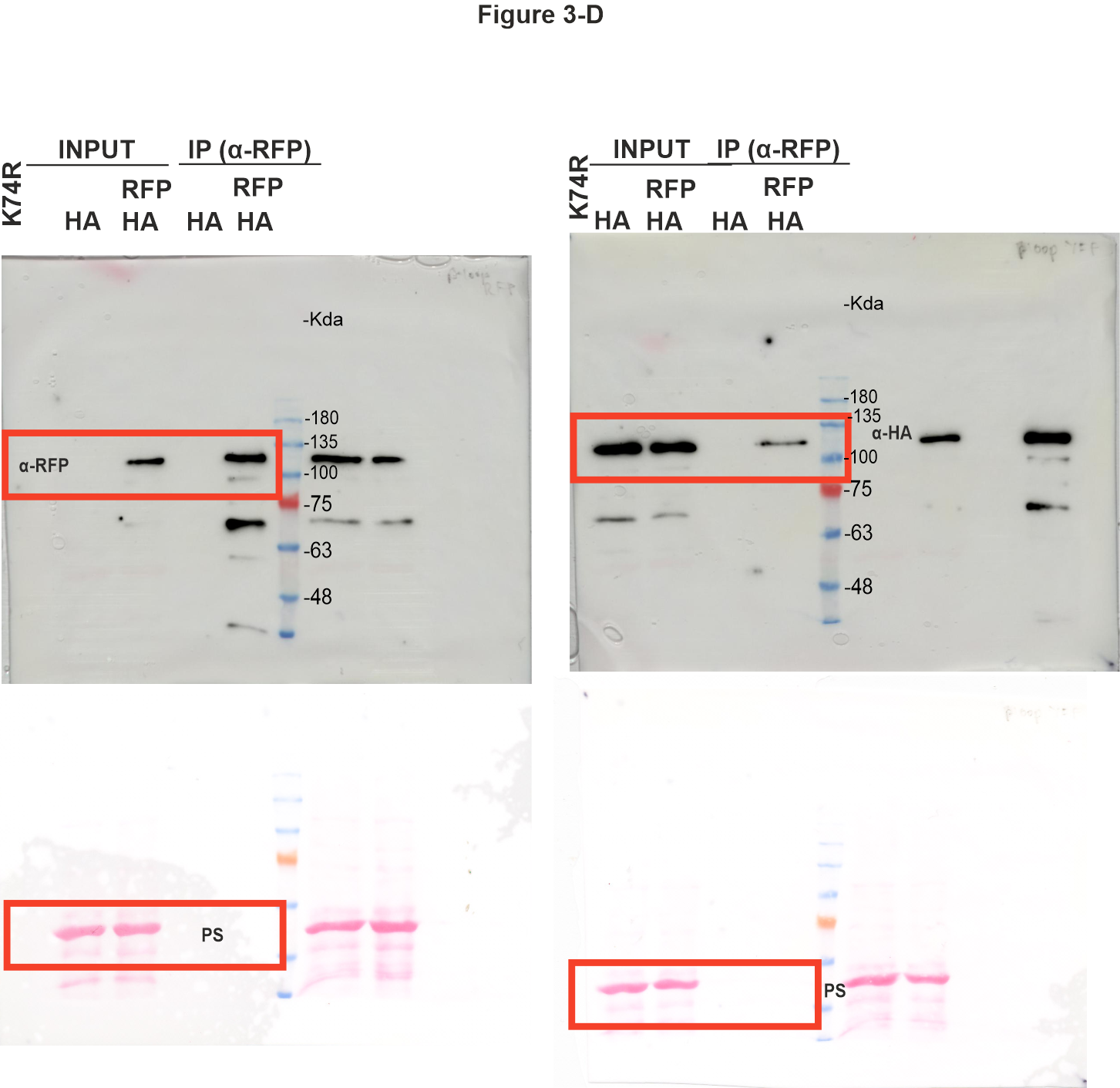

Supplement: Supplementary file 8 — Source data Fig. 3 [file 44319_2024_240_MOESM8_ESM.zip › Figure 3/3D/Fig 3 D.png]

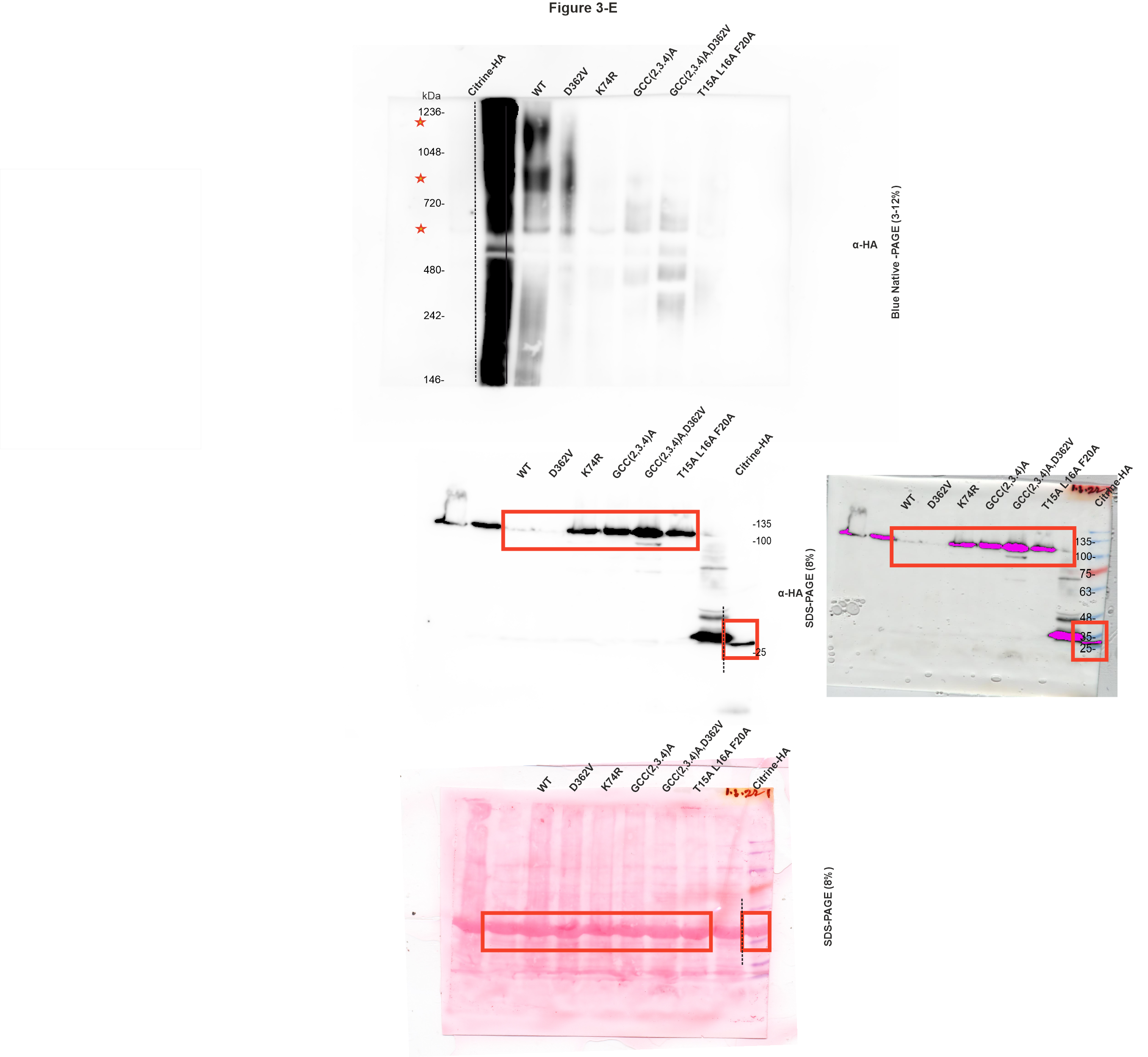

Supplement: Supplementary file 8 — Source data Fig. 3 [file 44319_2024_240_MOESM8_ESM.zip › Figure 3/3E/Fig 3 E.png]

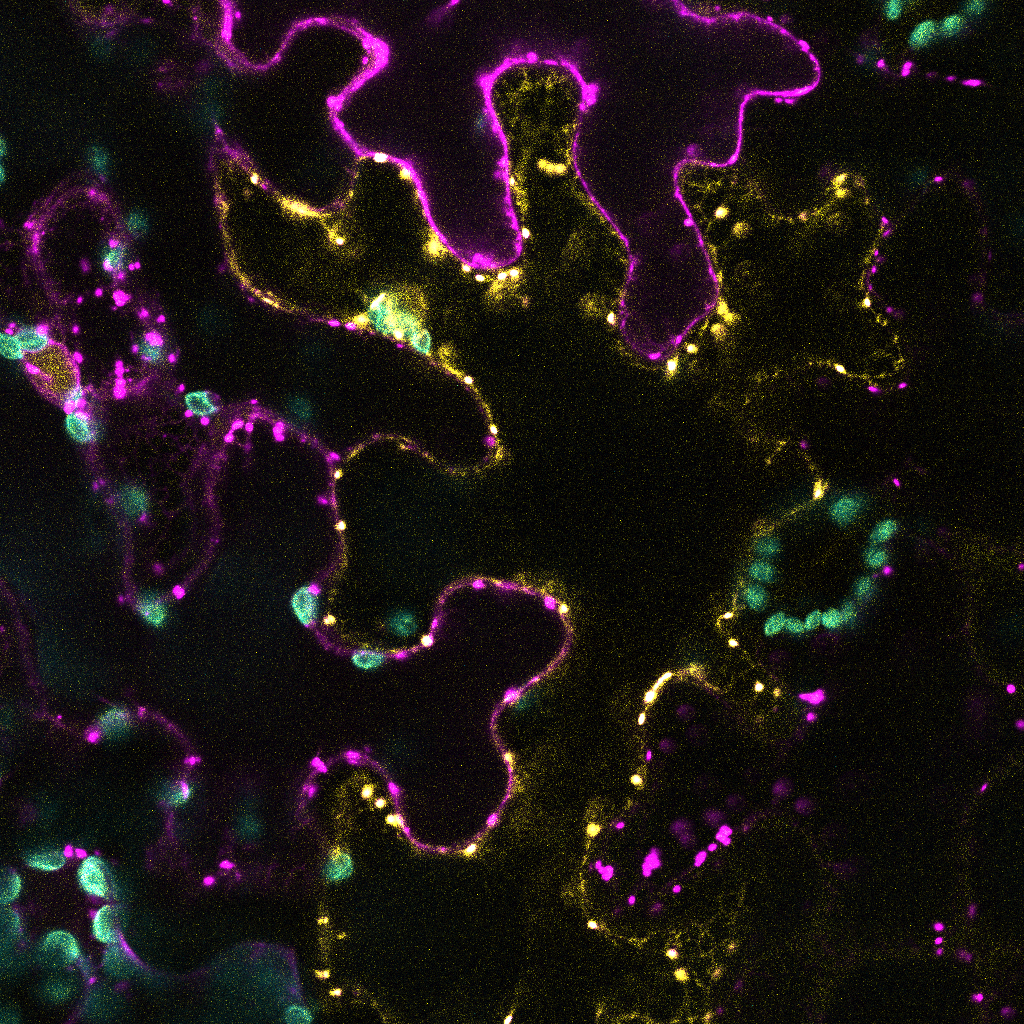

Supplement: Supplementary file 9 — Source data Fig. 4 [file 44319_2024_240_MOESM9_ESM.zip › Figure 4/Figure 4A/Fig(4-A)-PML5+Golgi.tiff]

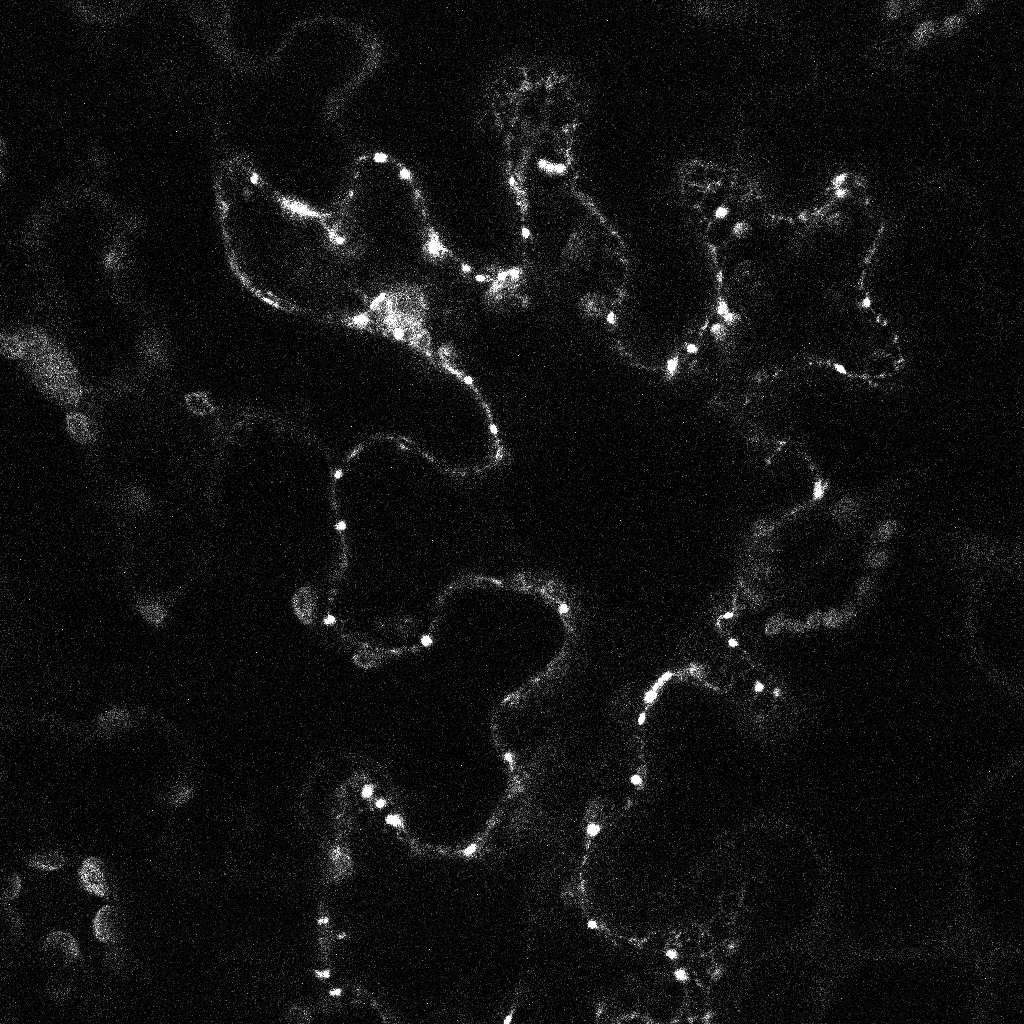

Supplement: Supplementary file 9 — Source data Fig. 4 [file 44319_2024_240_MOESM9_ESM.zip › Figure 4/Figure 4A/Fig(4-A)-PML5+Golgi.tiff_files/Fig(4-A)-PML5+Golgi_b0v0t0z0c0x0-1024y0-1024.tiff]

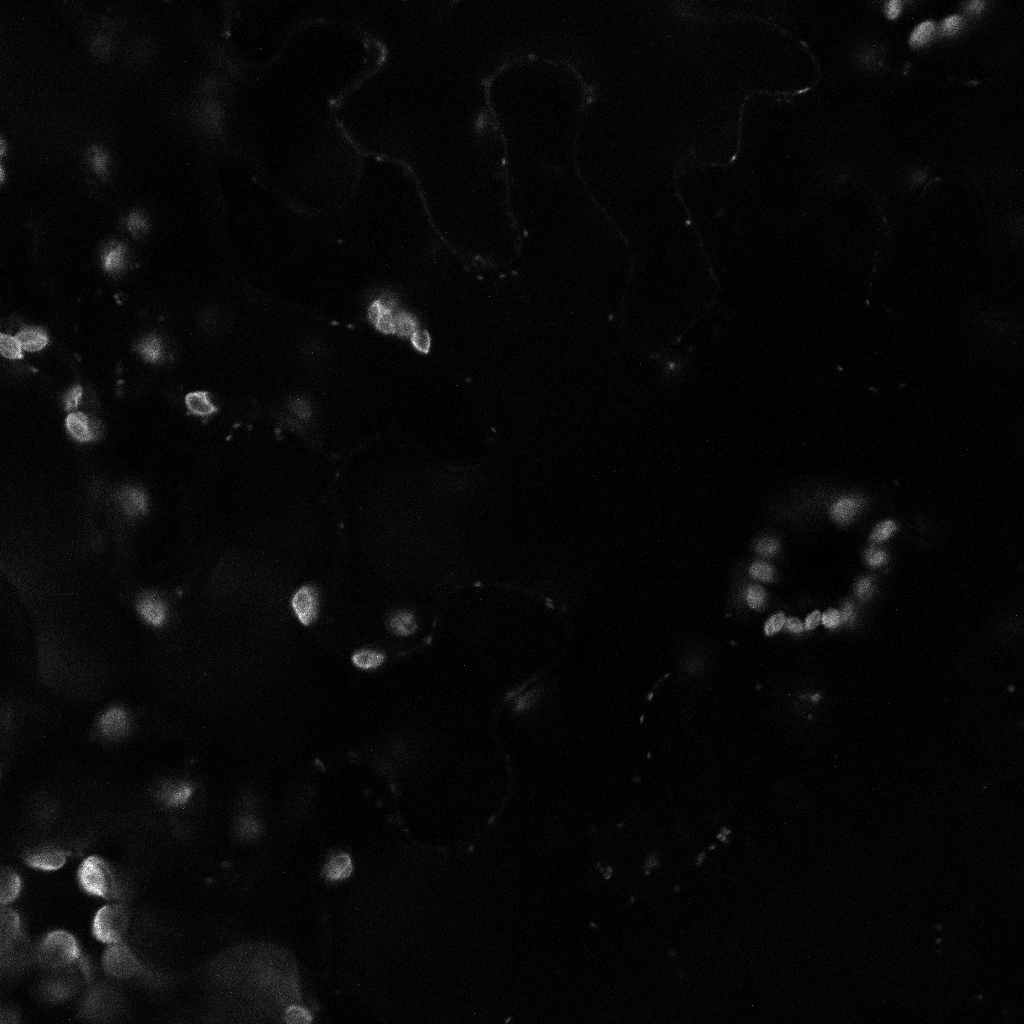

Supplement: Supplementary file 9 — Source data Fig. 4 [file 44319_2024_240_MOESM9_ESM.zip › Figure 4/Figure 4A/Fig(4-A)-PML5+Golgi.tiff_files/Fig(4-A)-PML5+Golgi_b0v0t0z0c1x0-1024y0-1024.tiff]

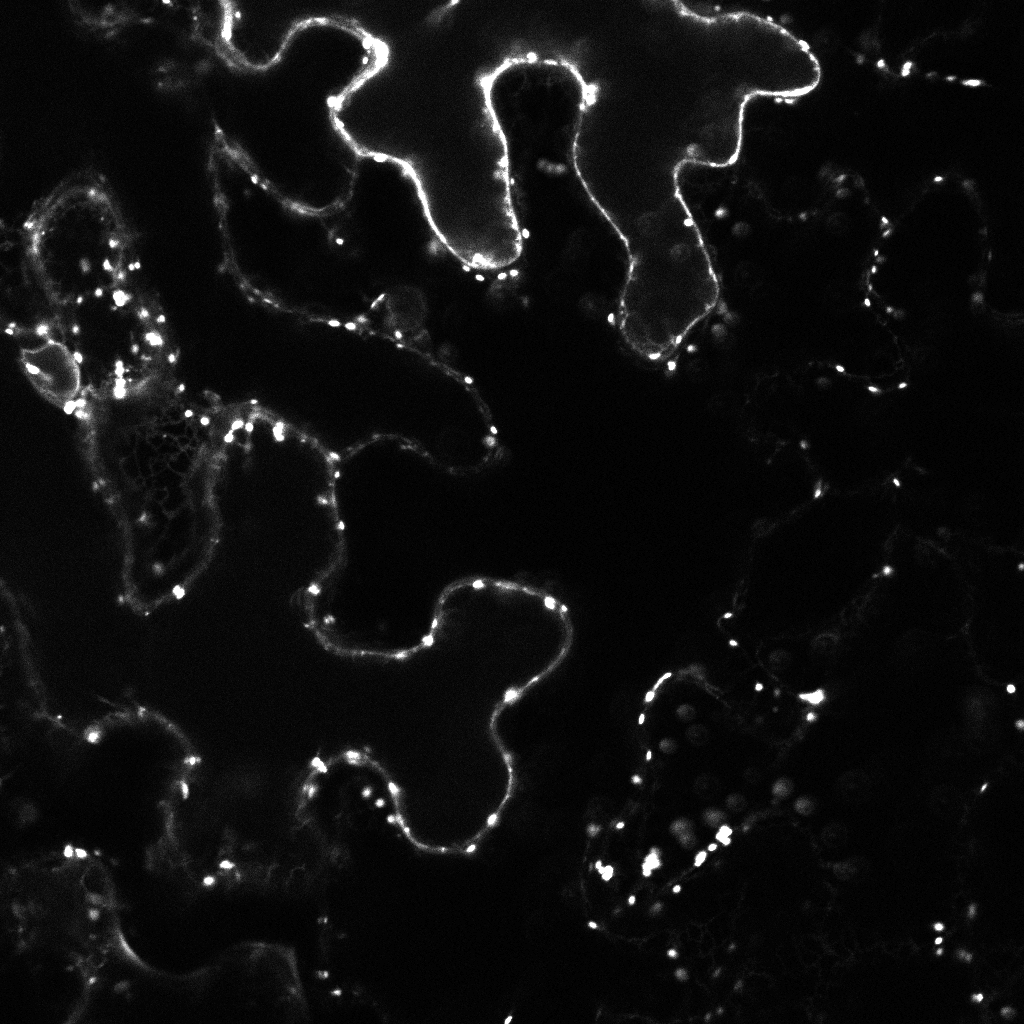

Supplement: Supplementary file 9 — Source data Fig. 4 [file 44319_2024_240_MOESM9_ESM.zip › Figure 4/Figure 4A/Fig(4-A)-PML5+Golgi.tiff_files/Fig(4-A)-PML5+Golgi_b0v0t0z0c2x0-1024y0-1024.tiff]

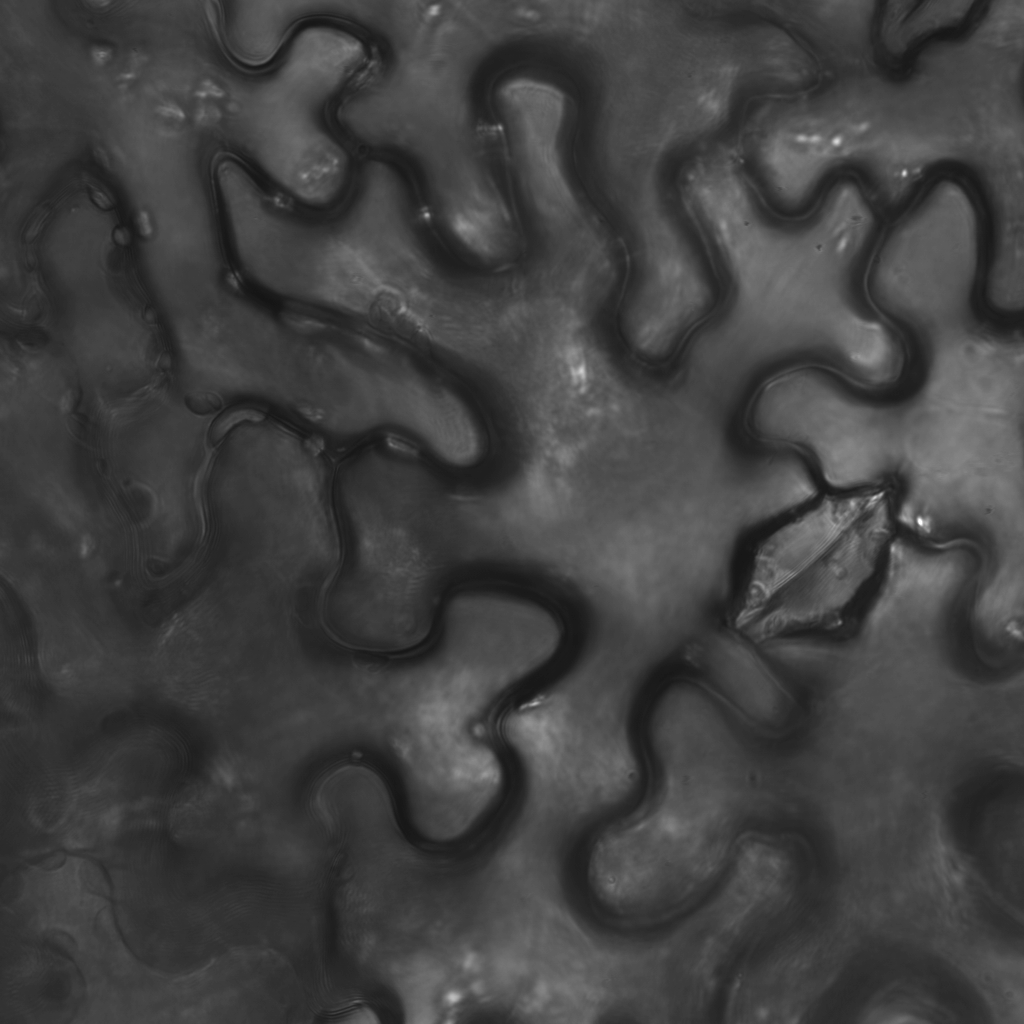

Supplement: Supplementary file 9 — Source data Fig. 4 [file 44319_2024_240_MOESM9_ESM.zip › Figure 4/Figure 4A/Fig(4-A)-PML5+Golgi.tiff_files/Fig(4-A)-PML5+Golgi_b0v0t0z0c3x0-1024y0-1024.tiff]

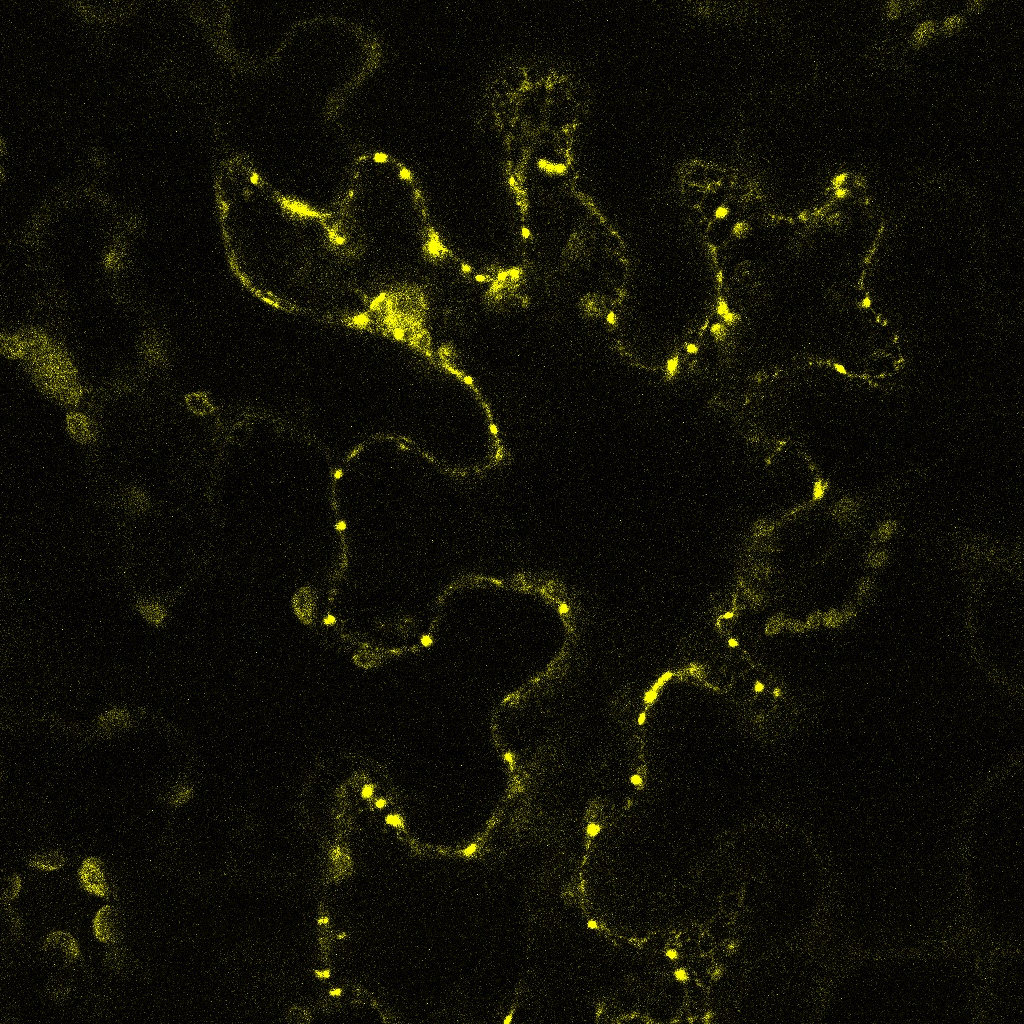

Supplement: Supplementary file 9 — Source data Fig. 4 [file 44319_2024_240_MOESM9_ESM.zip › Figure 4/Figure 4A/Fig(4-A)-PML5+Golgi/Image 59_c1.jpg]

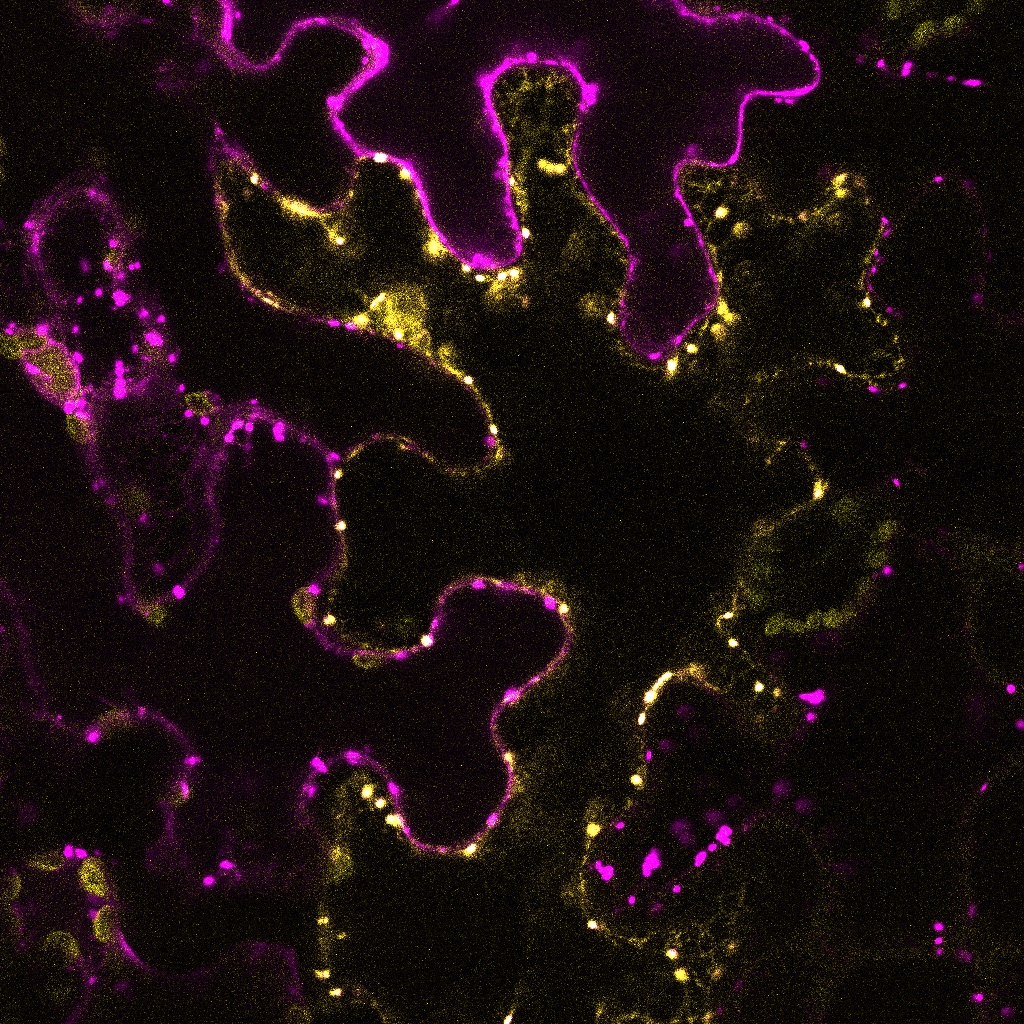

Supplement: Supplementary file 9 — Source data Fig. 4 [file 44319_2024_240_MOESM9_ESM.zip › Figure 4/Figure 4A/Fig(4-A)-PML5+Golgi/Image 59_c1-4.jpg]

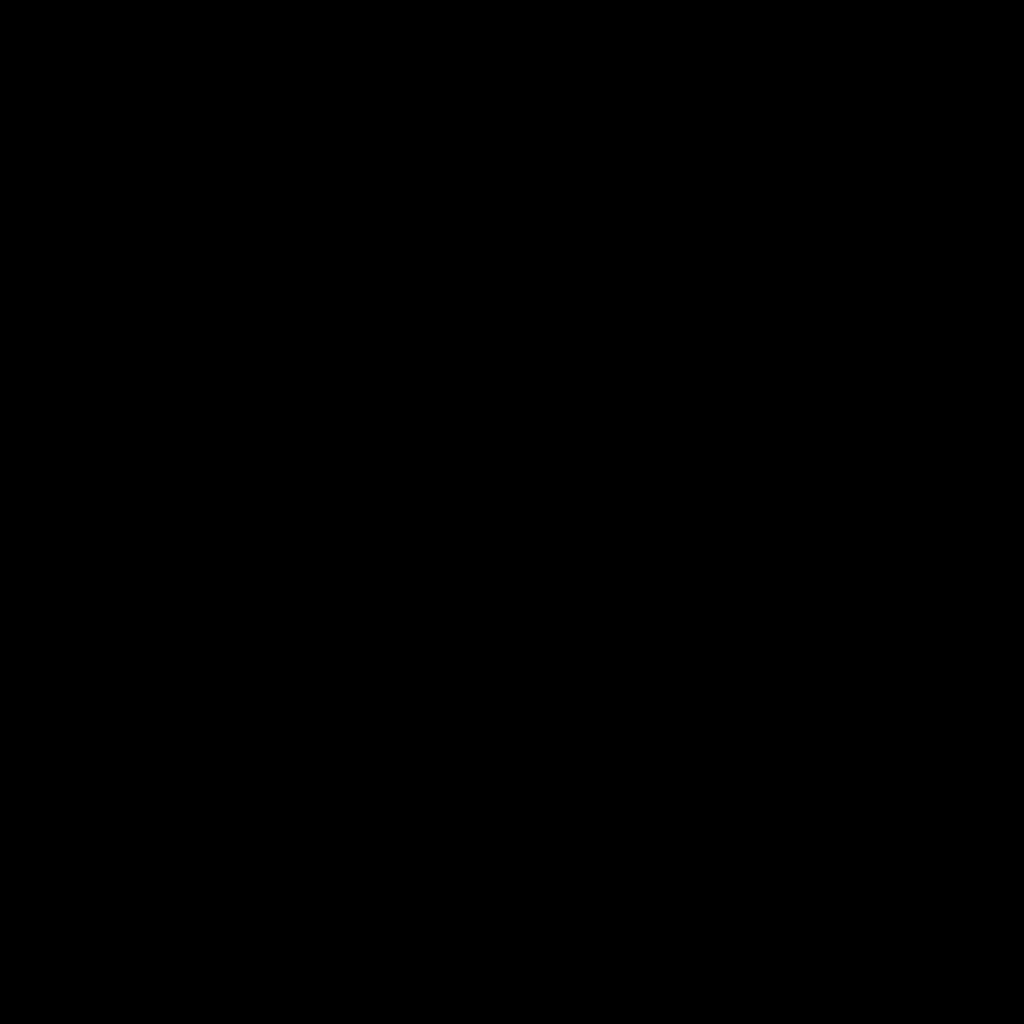

Supplement: Supplementary file 9 — Source data Fig. 4 [file 44319_2024_240_MOESM9_ESM.zip › Figure 4/Figure 4A/Fig(4-A)-PML5+Golgi/Image 59_c2.jpg]

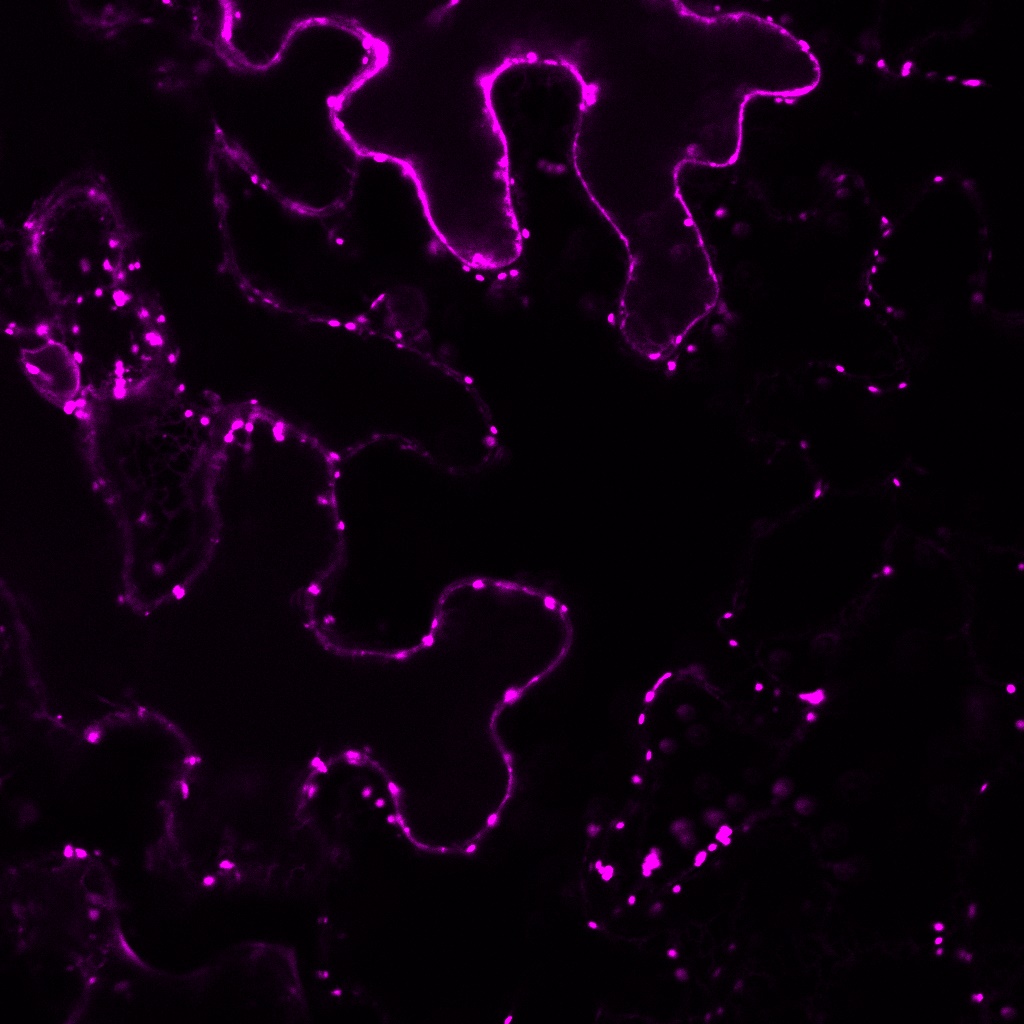

Supplement: Supplementary file 9 — Source data Fig. 4 [file 44319_2024_240_MOESM9_ESM.zip › Figure 4/Figure 4A/Fig(4-A)-PML5+Golgi/Image 59_c3.jpg]

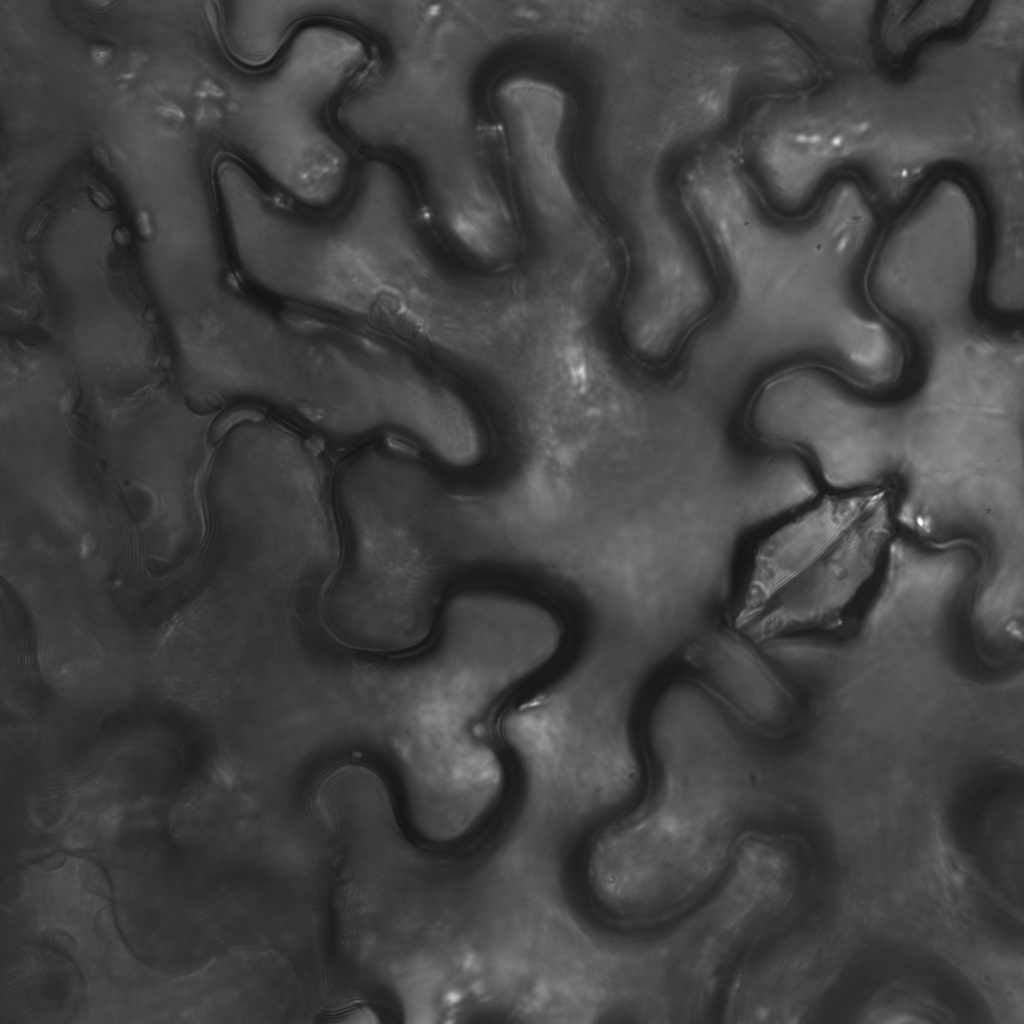

Supplement: Supplementary file 9 — Source data Fig. 4 [file 44319_2024_240_MOESM9_ESM.zip › Figure 4/Figure 4A/Fig(4-A)-PML5+Golgi/Image 59_c4.jpg]

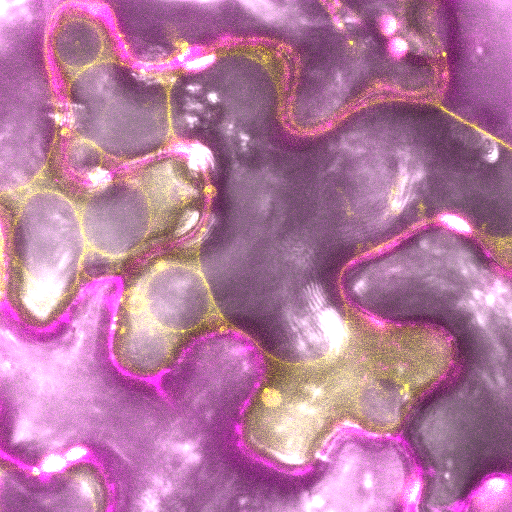

Supplement: Supplementary file 9 — Source data Fig. 4 [file 44319_2024_240_MOESM9_ESM.zip › Figure 4/Figure 4B/Fig(4-B)-PML5+Vacuolar.tiff]

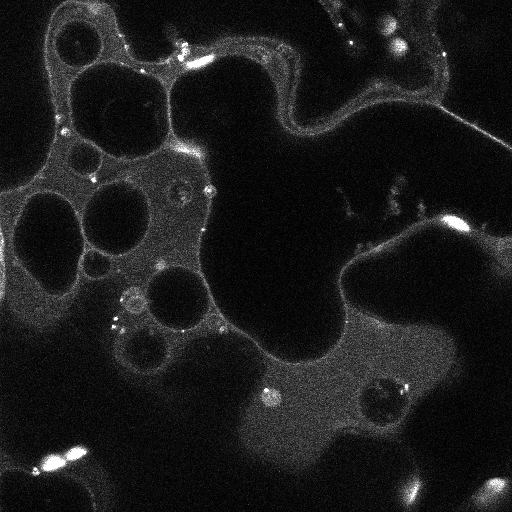

Supplement: Supplementary file 9 — Source data Fig. 4 [file 44319_2024_240_MOESM9_ESM.zip › Figure 4/Figure 4B/Fig(4-B)-PML5+Vacuolar.tiff_files/Fig(4-B)-PML5+Vacuolar_b0v0t0z0c0x0-512y0-512.tiff]

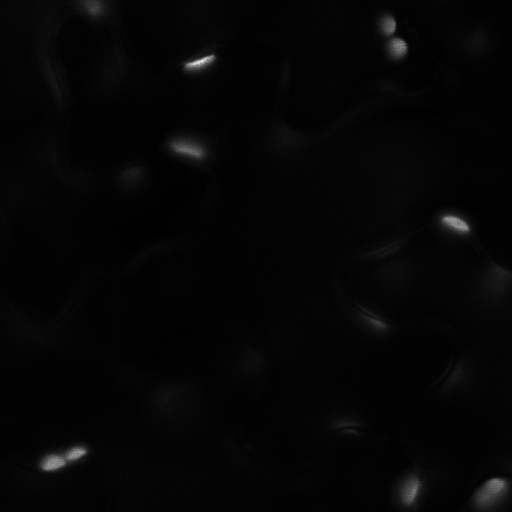

Supplement: Supplementary file 9 — Source data Fig. 4 [file 44319_2024_240_MOESM9_ESM.zip › Figure 4/Figure 4B/Fig(4-B)-PML5+Vacuolar.tiff_files/Fig(4-B)-PML5+Vacuolar_b0v0t0z0c1x0-512y0-512.tiff]

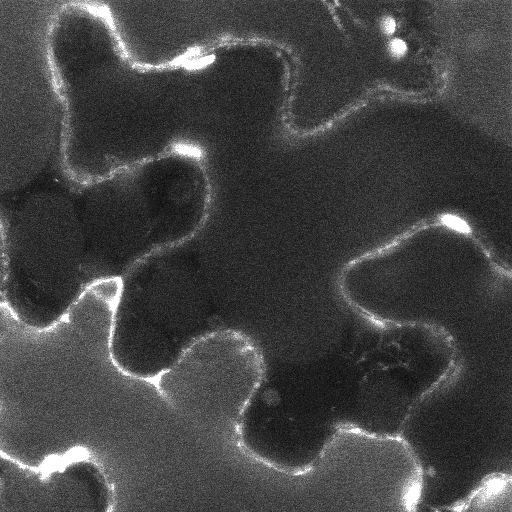

Supplement: Supplementary file 9 — Source data Fig. 4 [file 44319_2024_240_MOESM9_ESM.zip › Figure 4/Figure 4B/Fig(4-B)-PML5+Vacuolar.tiff_files/Fig(4-B)-PML5+Vacuolar_b0v0t0z0c2x0-512y0-512.tiff]

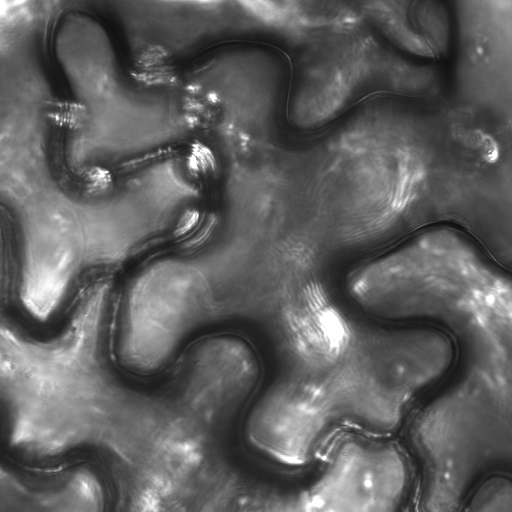

Supplement: Supplementary file 9 — Source data Fig. 4 [file 44319_2024_240_MOESM9_ESM.zip › Figure 4/Figure 4B/Fig(4-B)-PML5+Vacuolar.tiff_files/Fig(4-B)-PML5+Vacuolar_b0v0t0z0c3x0-512y0-512.tiff]

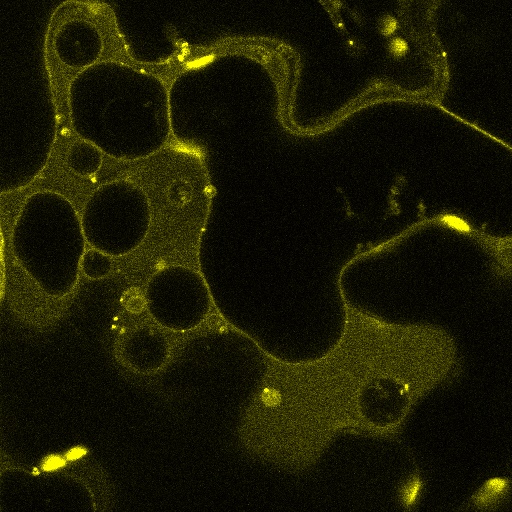

Supplement: Supplementary file 9 — Source data Fig. 4 [file 44319_2024_240_MOESM9_ESM.zip › Figure 4/Figure 4B/Fig(4-B)-PML5+Vacuolar-image/Image 73_c1.jpg]

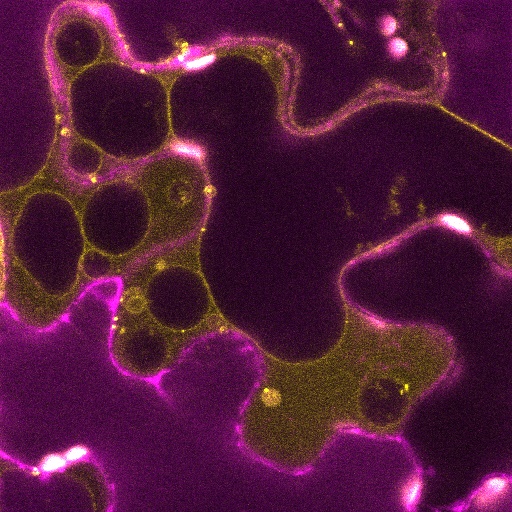

Supplement: Supplementary file 9 — Source data Fig. 4 [file 44319_2024_240_MOESM9_ESM.zip › Figure 4/Figure 4B/Fig(4-B)-PML5+Vacuolar-image/Image 73_c1-4.jpg]

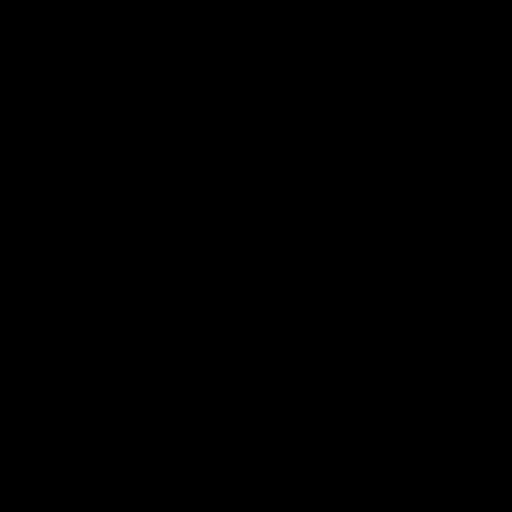

Supplement: Supplementary file 9 — Source data Fig. 4 [file 44319_2024_240_MOESM9_ESM.zip › Figure 4/Figure 4B/Fig(4-B)-PML5+Vacuolar-image/Image 73_c2.jpg]

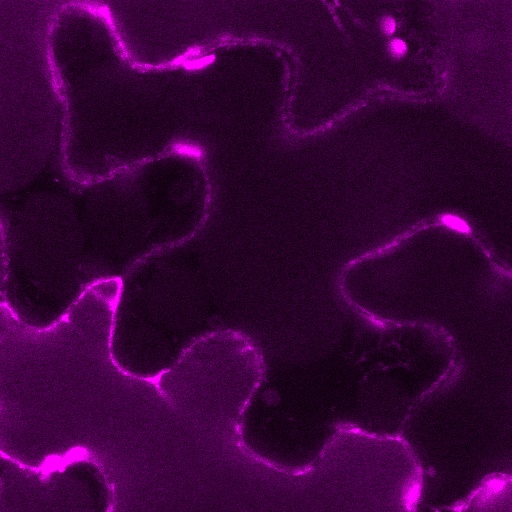

Supplement: Supplementary file 9 — Source data Fig. 4 [file 44319_2024_240_MOESM9_ESM.zip › Figure 4/Figure 4B/Fig(4-B)-PML5+Vacuolar-image/Image 73_c3.jpg]

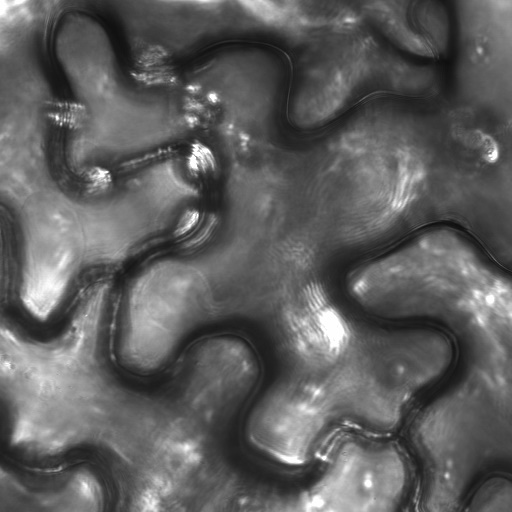

Supplement: Supplementary file 9 — Source data Fig. 4 [file 44319_2024_240_MOESM9_ESM.zip › Figure 4/Figure 4B/Fig(4-B)-PML5+Vacuolar-image/Image 73_c4.jpg]

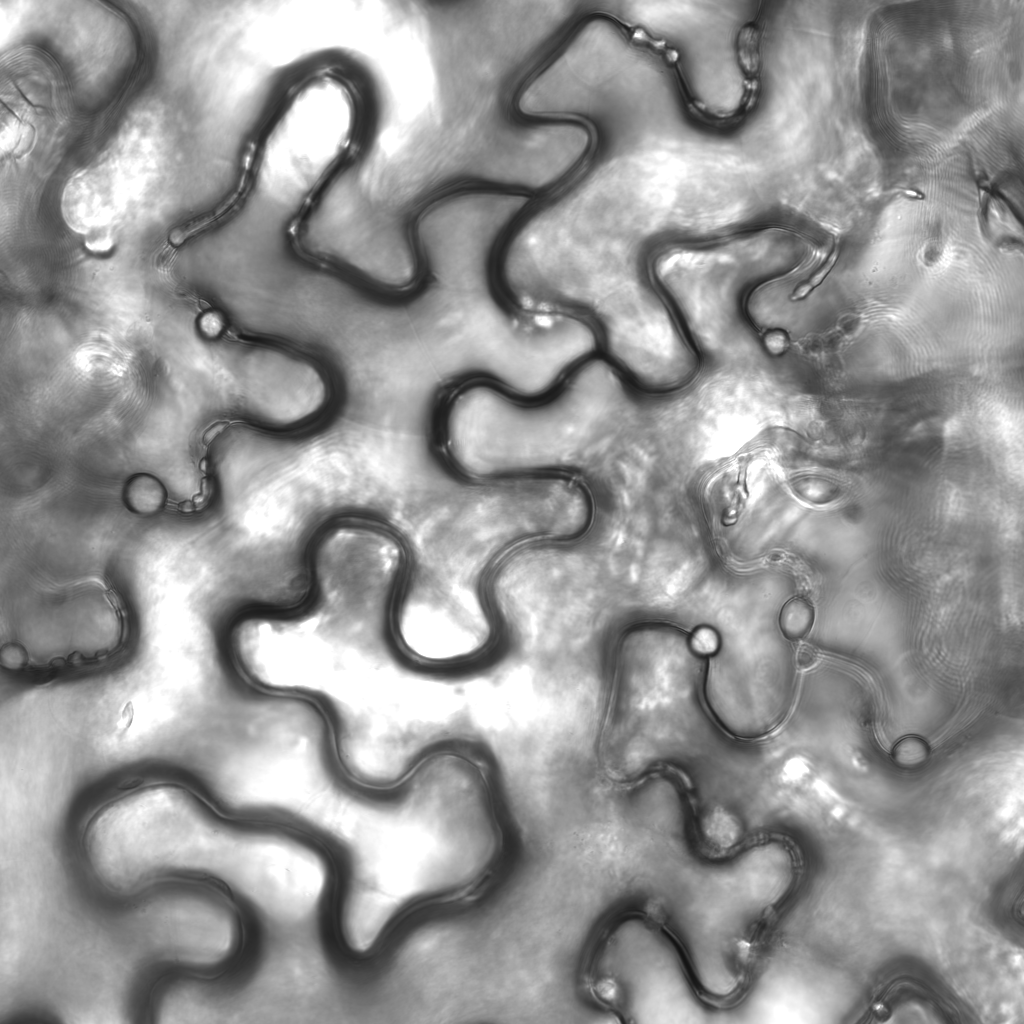

Supplement: Supplementary file 9 — Source data Fig. 4 [file 44319_2024_240_MOESM9_ESM.zip › Figure 4/Figure 4C/Black White/Bright Field.tif]

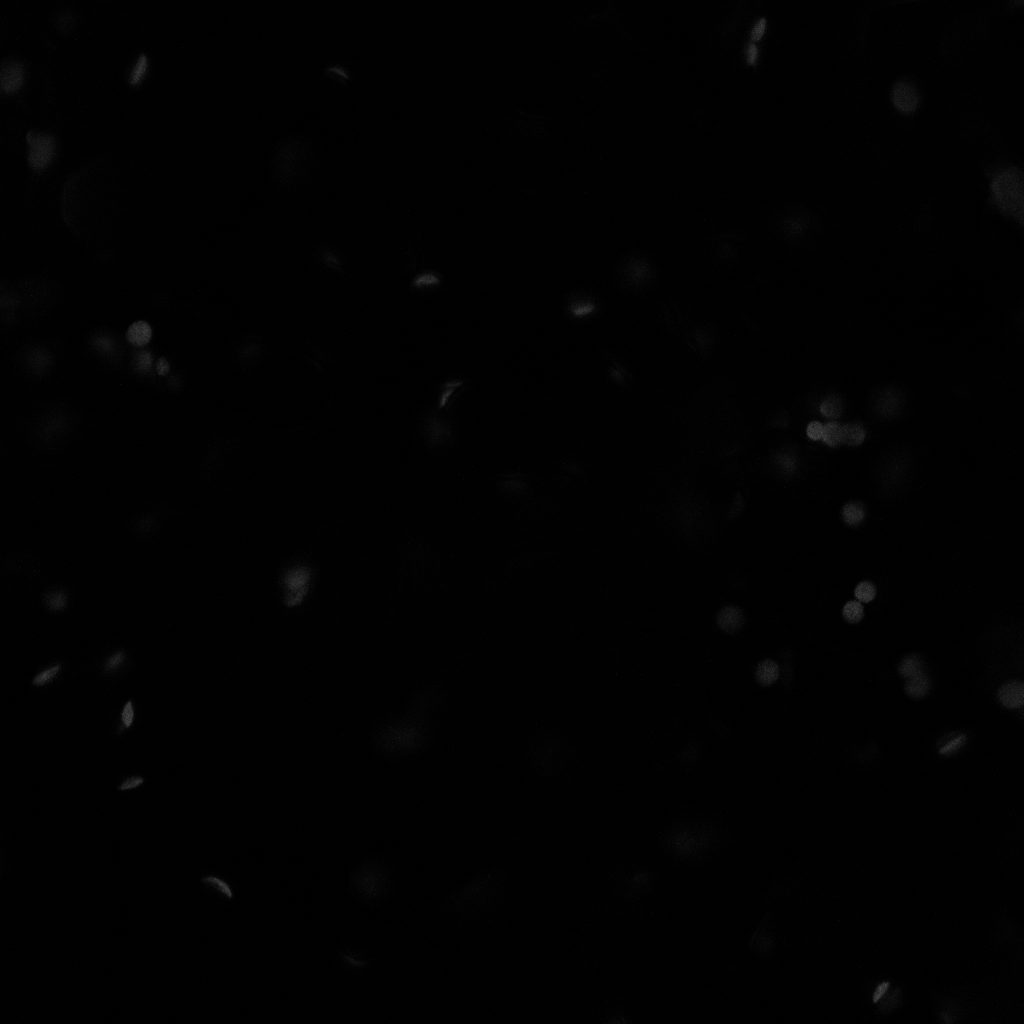

Supplement: Supplementary file 9 — Source data Fig. 4 [file 44319_2024_240_MOESM9_ESM.zip › Figure 4/Figure 4C/Black White/Cyan (ChlA).tif]

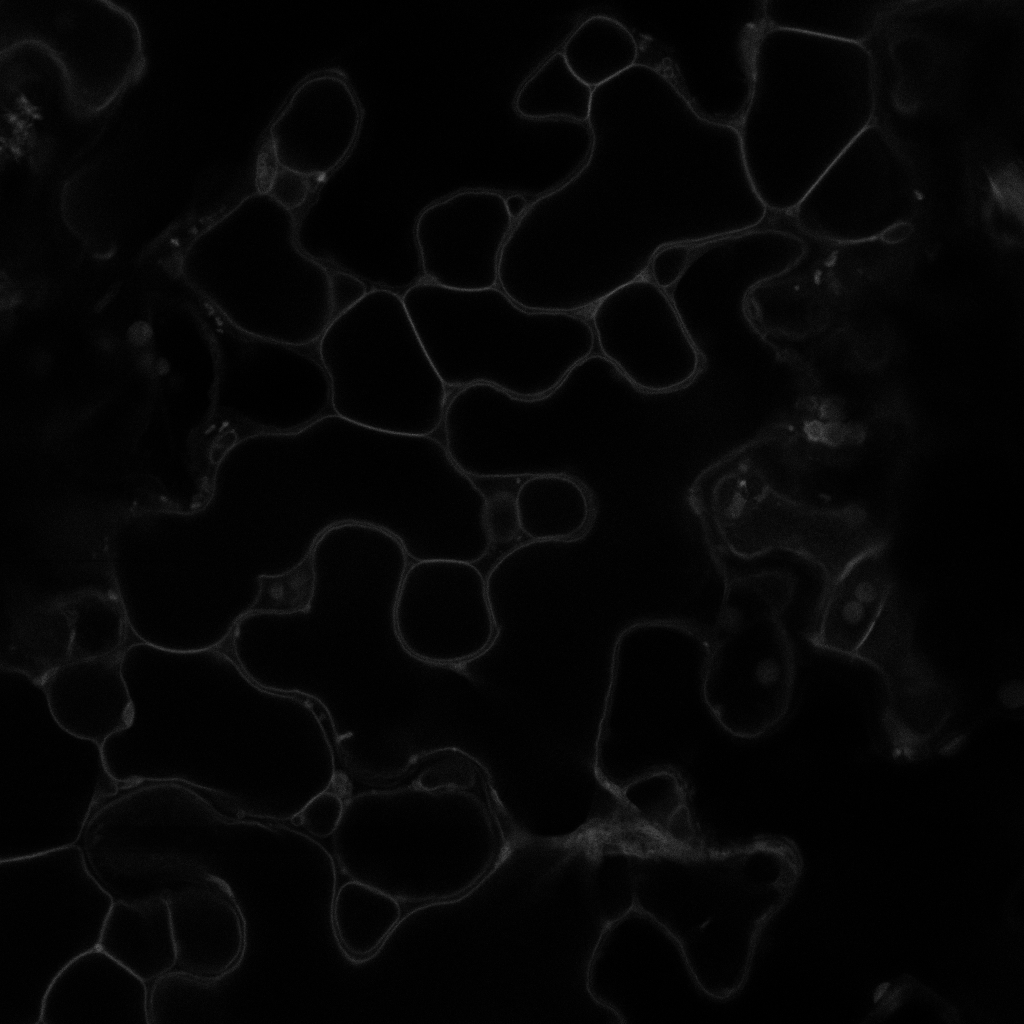

Supplement: Supplementary file 9 — Source data Fig. 4 [file 44319_2024_240_MOESM9_ESM.zip › Figure 4/Figure 4C/Black White/RFP (CBL6-mCherry).tif]

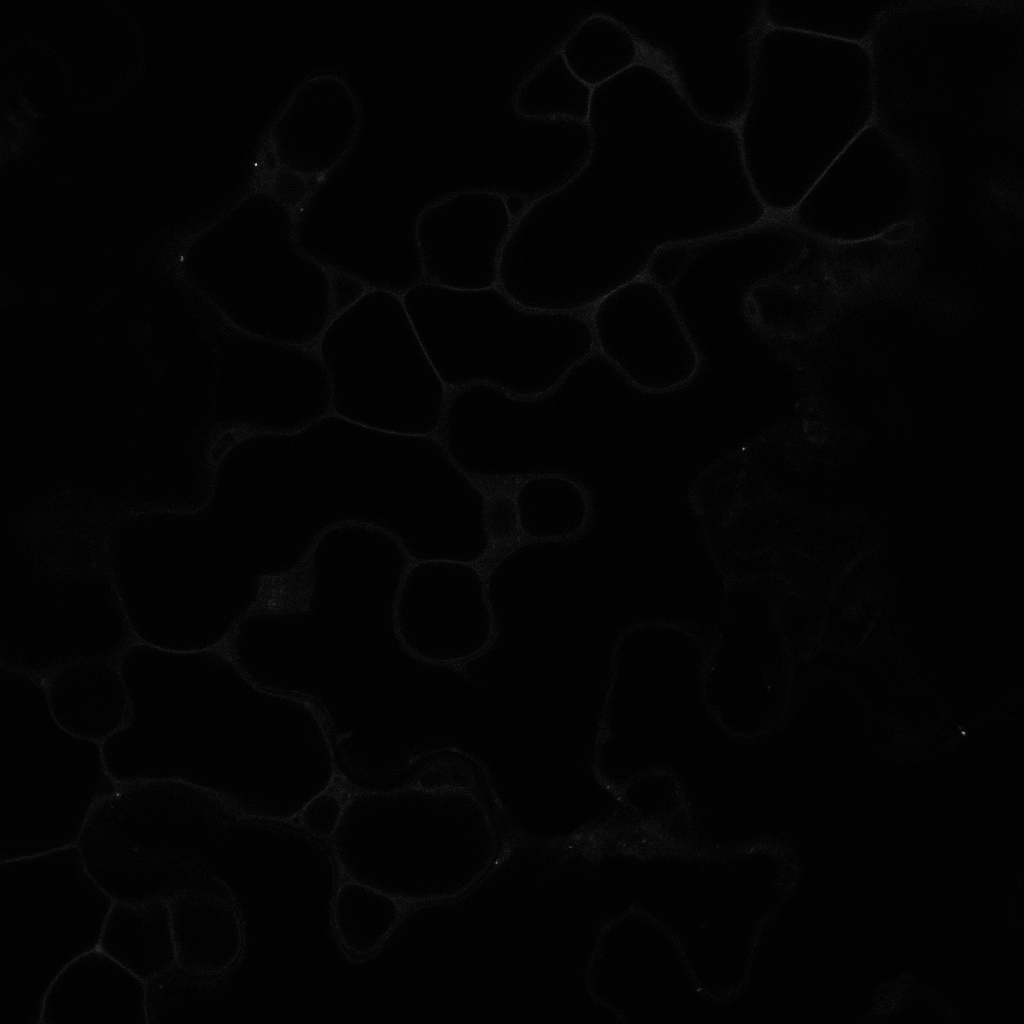

Supplement: Supplementary file 9 — Source data Fig. 4 [file 44319_2024_240_MOESM9_ESM.zip › Figure 4/Figure 4C/Black White/YFP (PML5-Citrine-HA).tif]

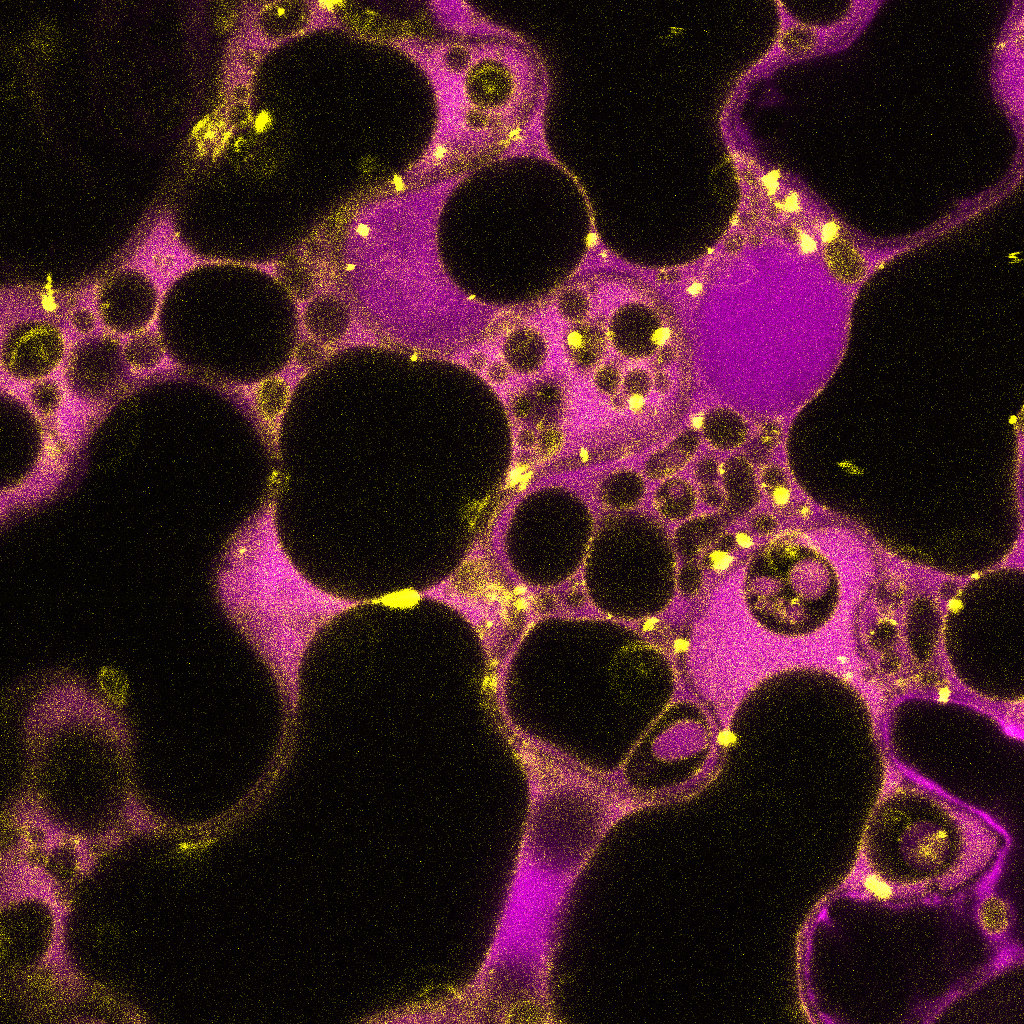

Supplement: Supplementary file 9 — Source data Fig. 4 [file 44319_2024_240_MOESM9_ESM.zip › Figure 4/Figure 4D/Fig(4-D)-PML5+Nucleo-Cytosol.tiff]

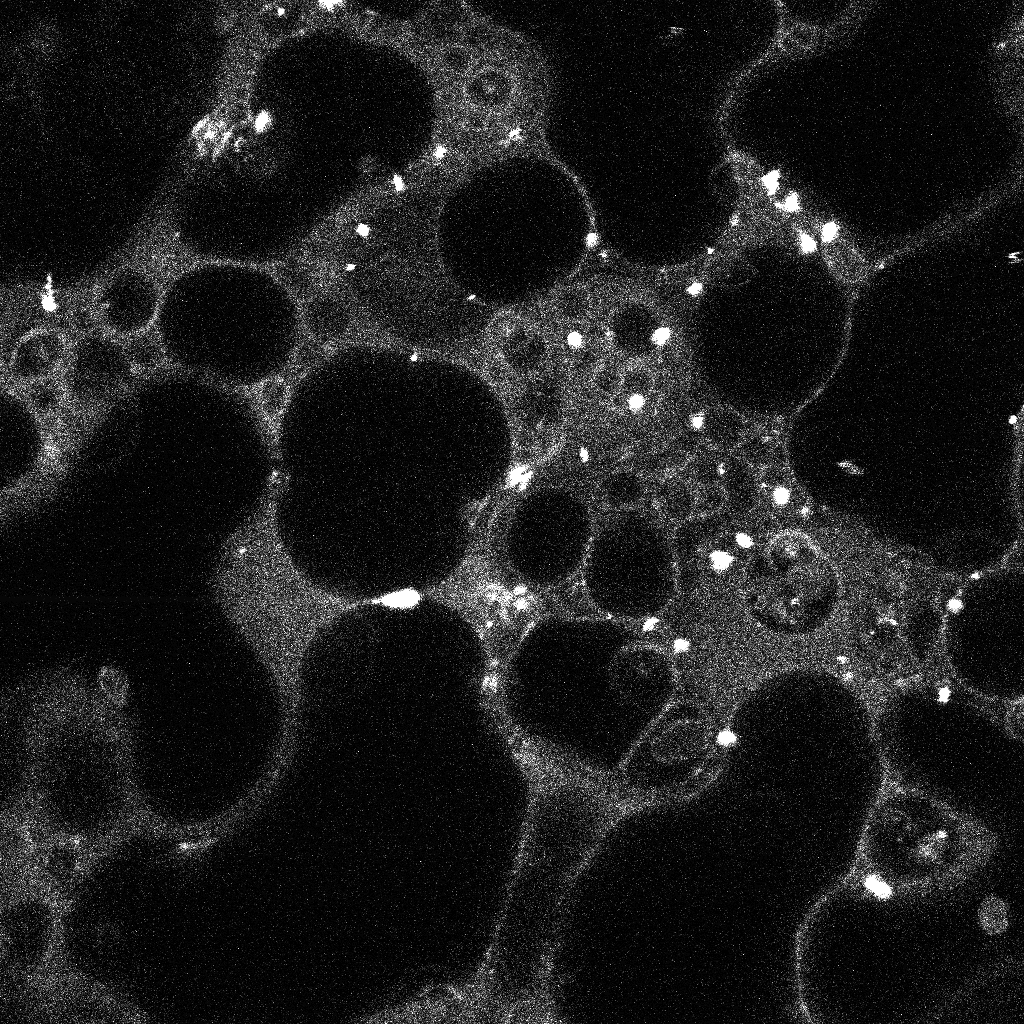

Supplement: Supplementary file 9 — Source data Fig. 4 [file 44319_2024_240_MOESM9_ESM.zip › Figure 4/Figure 4D/Fig(4-D)-PML5+Nucleo-Cytosol.tiff_files/Fig(4-D)-PML5+Nucleo-Cytosol_b0v0t0z0c0x0-1024y0-1024.tiff]

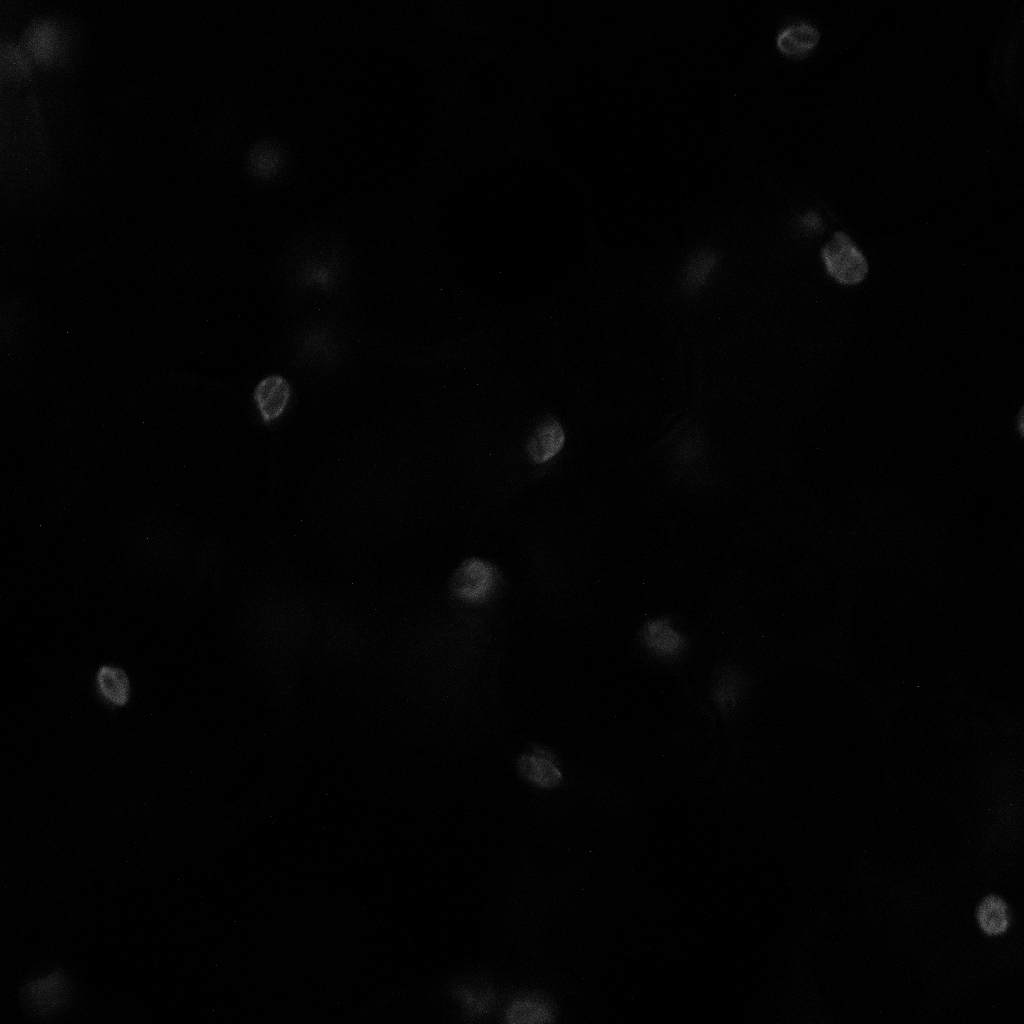

Supplement: Supplementary file 9 — Source data Fig. 4 [file 44319_2024_240_MOESM9_ESM.zip › Figure 4/Figure 4D/Fig(4-D)-PML5+Nucleo-Cytosol.tiff_files/Fig(4-D)-PML5+Nucleo-Cytosol_b0v0t0z0c1x0-1024y0-1024.tiff]

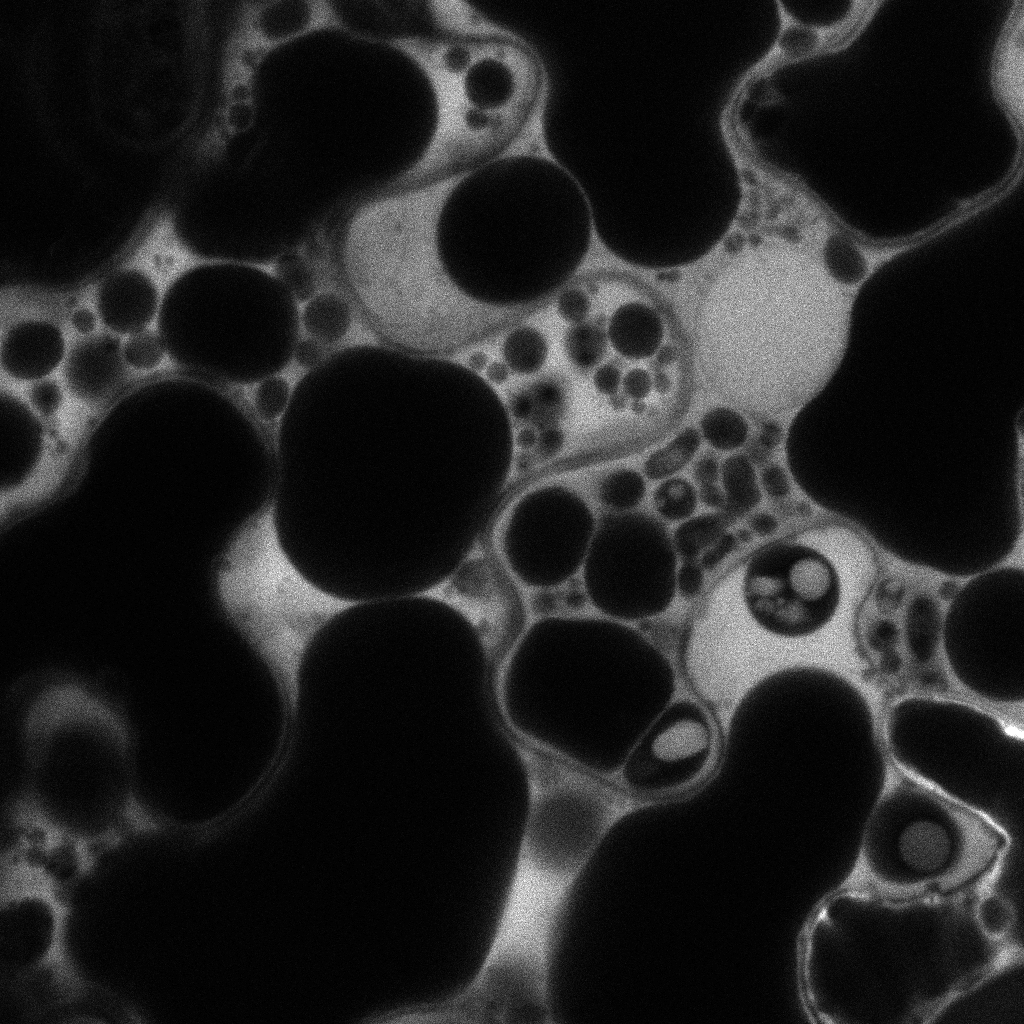

Supplement: Supplementary file 9 — Source data Fig. 4 [file 44319_2024_240_MOESM9_ESM.zip › Figure 4/Figure 4D/Fig(4-D)-PML5+Nucleo-Cytosol.tiff_files/Fig(4-D)-PML5+Nucleo-Cytosol_b0v0t0z0c2x0-1024y0-1024.tiff]

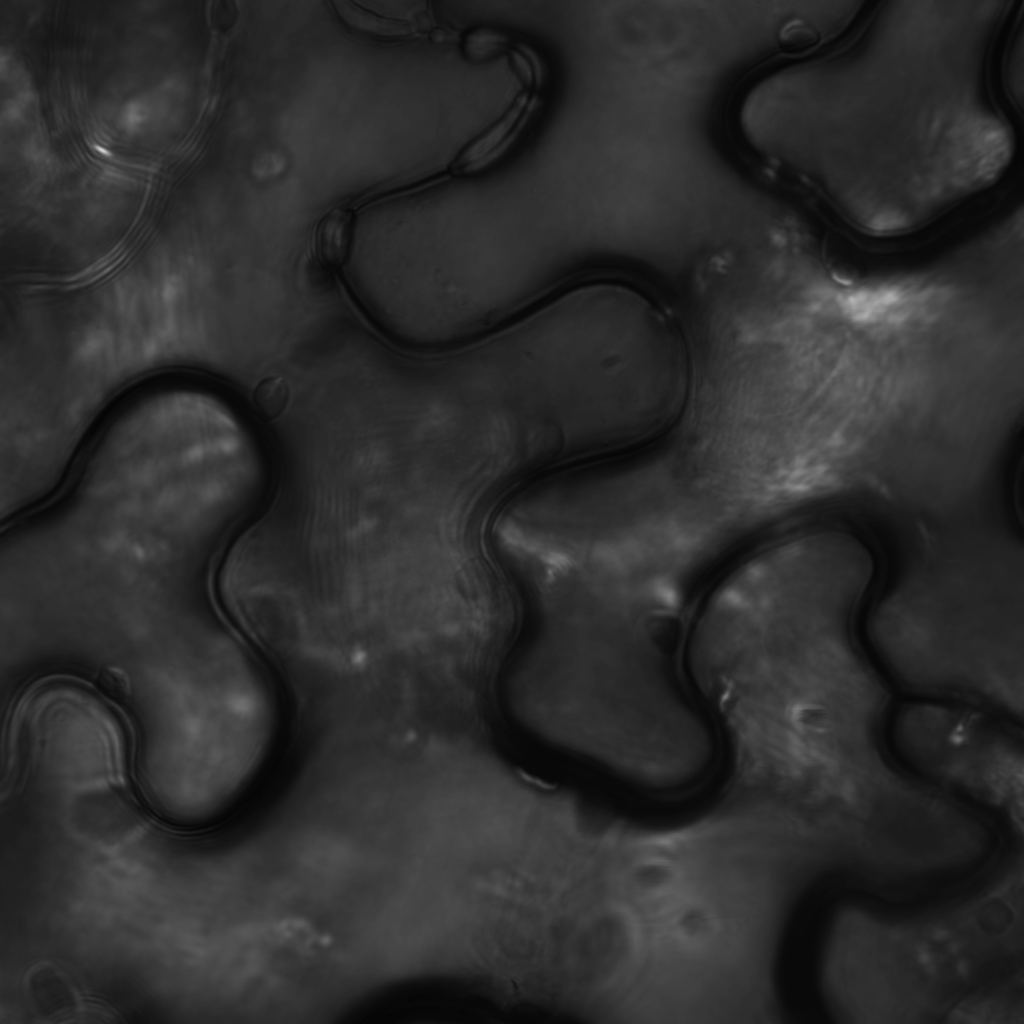

Supplement: Supplementary file 9 — Source data Fig. 4 [file 44319_2024_240_MOESM9_ESM.zip › Figure 4/Figure 4D/Fig(4-D)-PML5+Nucleo-Cytosol.tiff_files/Fig(4-D)-PML5+Nucleo-Cytosol_b0v0t0z0c3x0-1024y0-1024.tiff]

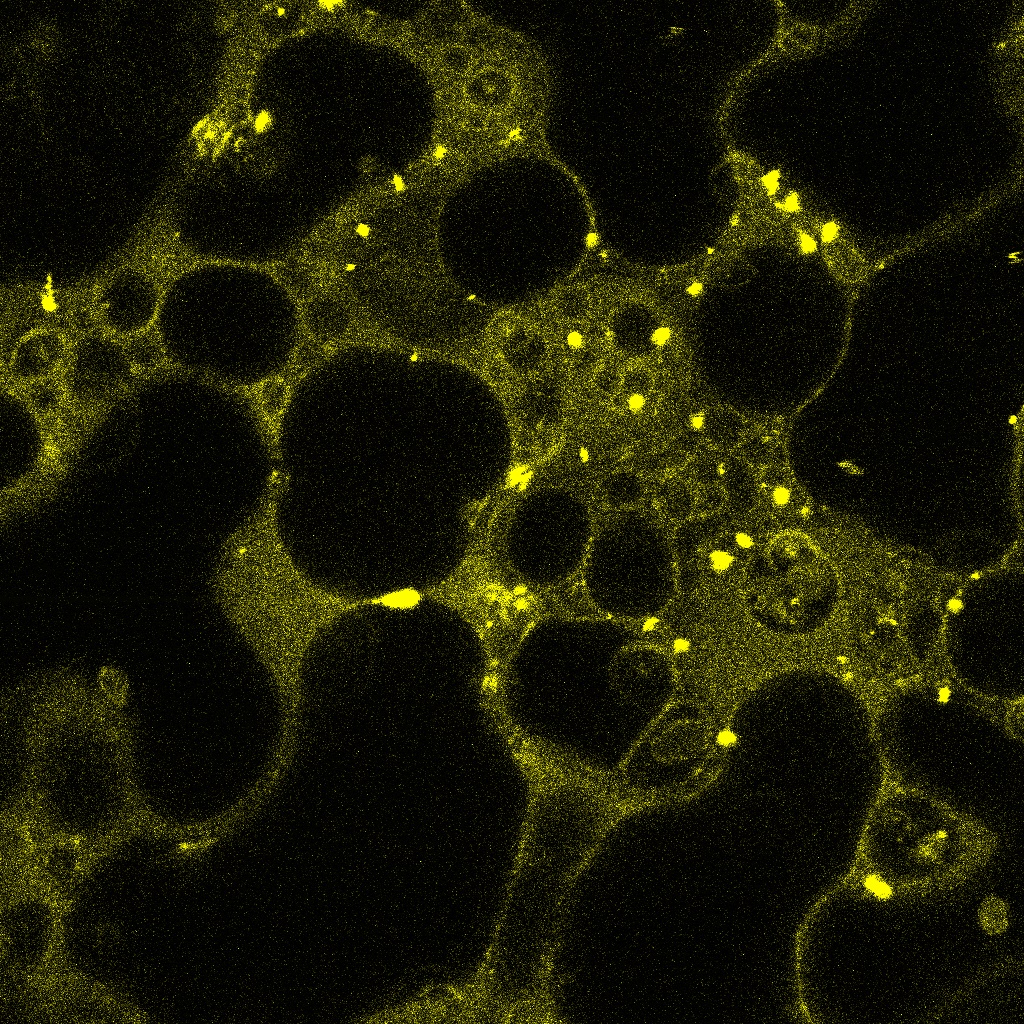

Supplement: Supplementary file 9 — Source data Fig. 4 [file 44319_2024_240_MOESM9_ESM.zip › Figure 4/Figure 4D/Fig(4-D)-PML5+Nucleo-Cytosol/Image 71 vacuole und cytosol engulfment_c1.jpg]

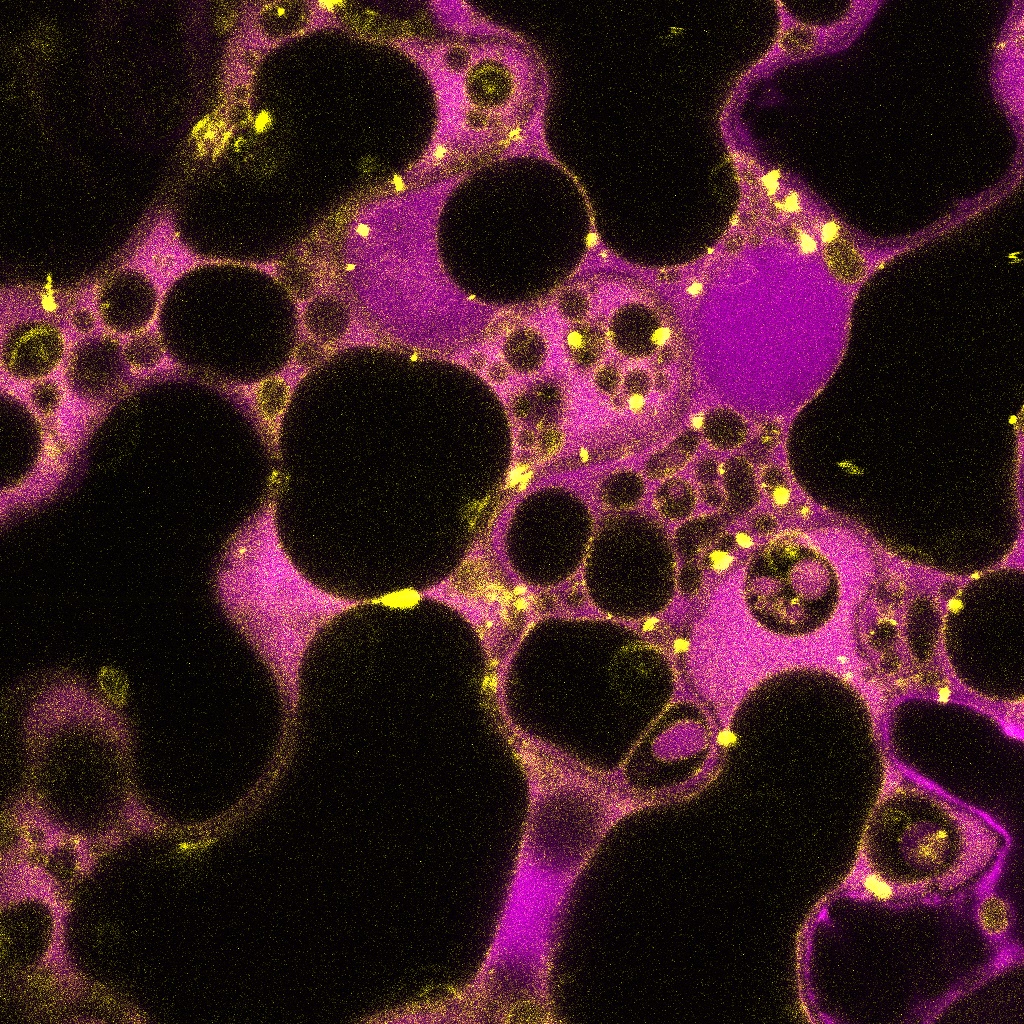

Supplement: Supplementary file 9 — Source data Fig. 4 [file 44319_2024_240_MOESM9_ESM.zip › Figure 4/Figure 4D/Fig(4-D)-PML5+Nucleo-Cytosol/Image 71 vacuole und cytosol engulfment_c1-4.jpg]

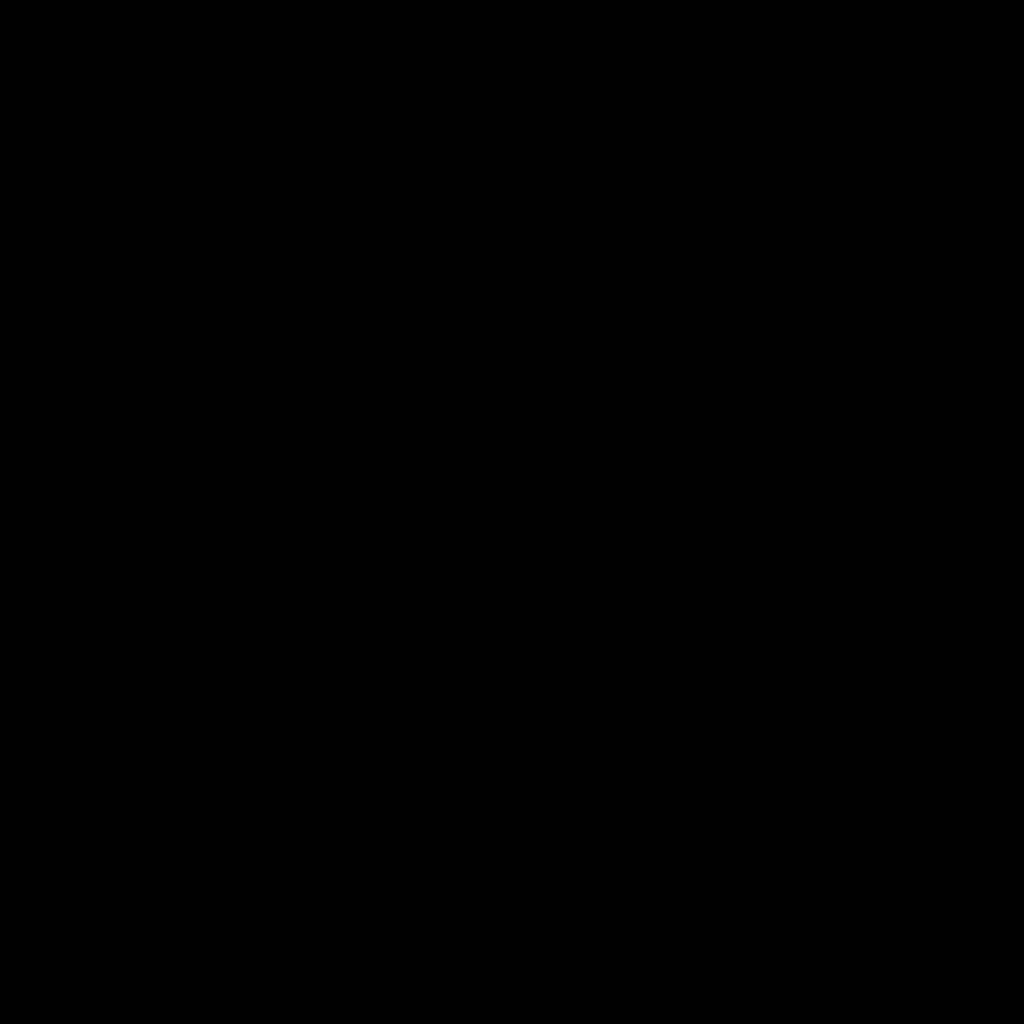

Supplement: Supplementary file 9 — Source data Fig. 4 [file 44319_2024_240_MOESM9_ESM.zip › Figure 4/Figure 4D/Fig(4-D)-PML5+Nucleo-Cytosol/Image 71 vacuole und cytosol engulfment_c2.jpg]

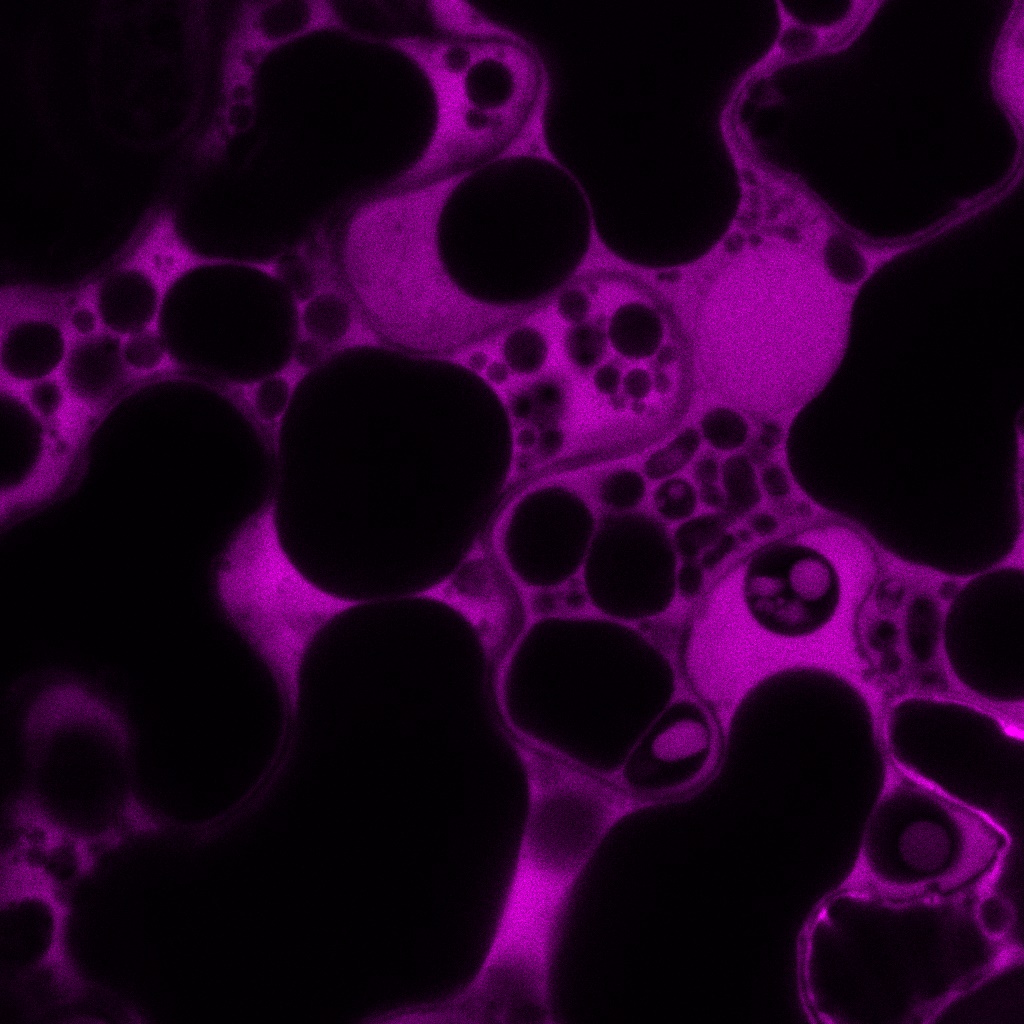

Supplement: Supplementary file 9 — Source data Fig. 4 [file 44319_2024_240_MOESM9_ESM.zip › Figure 4/Figure 4D/Fig(4-D)-PML5+Nucleo-Cytosol/Image 71 vacuole und cytosol engulfment_c3.jpg]

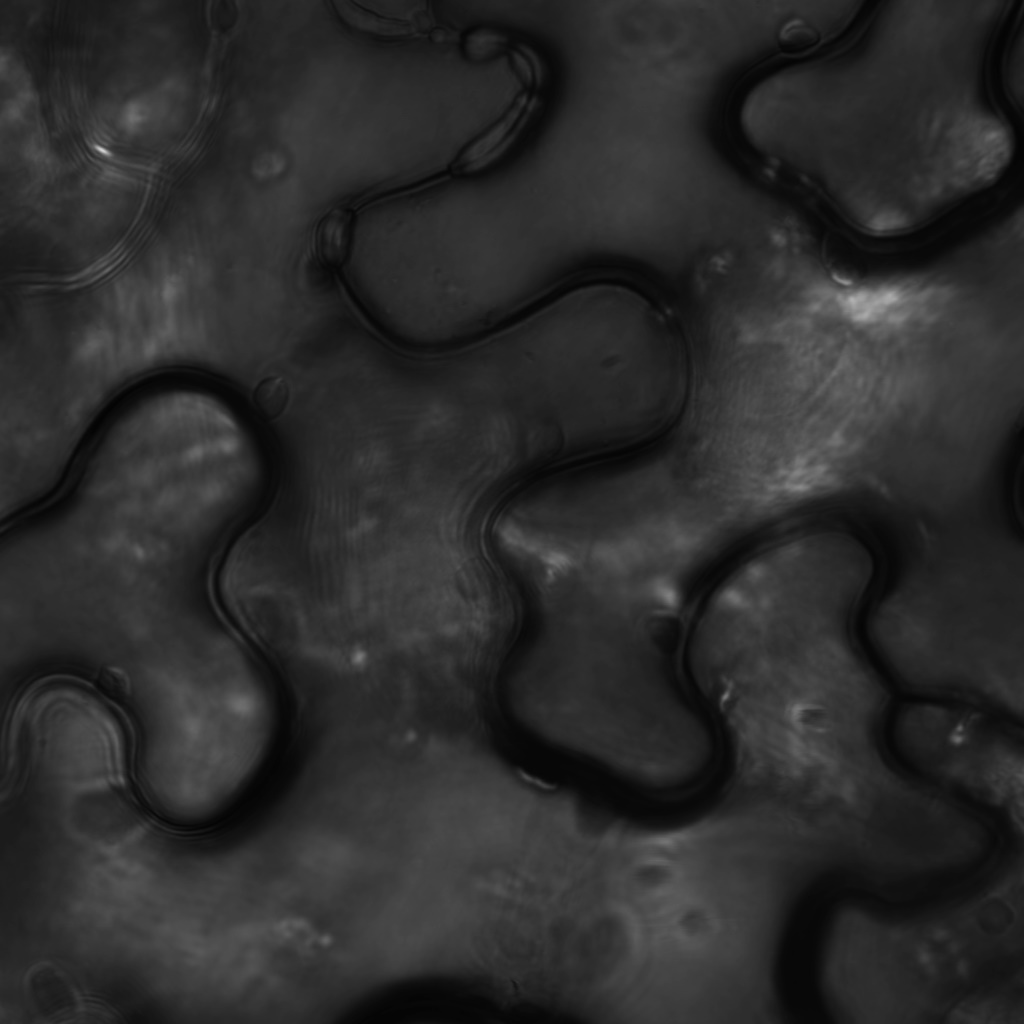

Supplement: Supplementary file 9 — Source data Fig. 4 [file 44319_2024_240_MOESM9_ESM.zip › Figure 4/Figure 4D/Fig(4-D)-PML5+Nucleo-Cytosol/Image 71 vacuole und cytosol engulfment_c4.jpg]

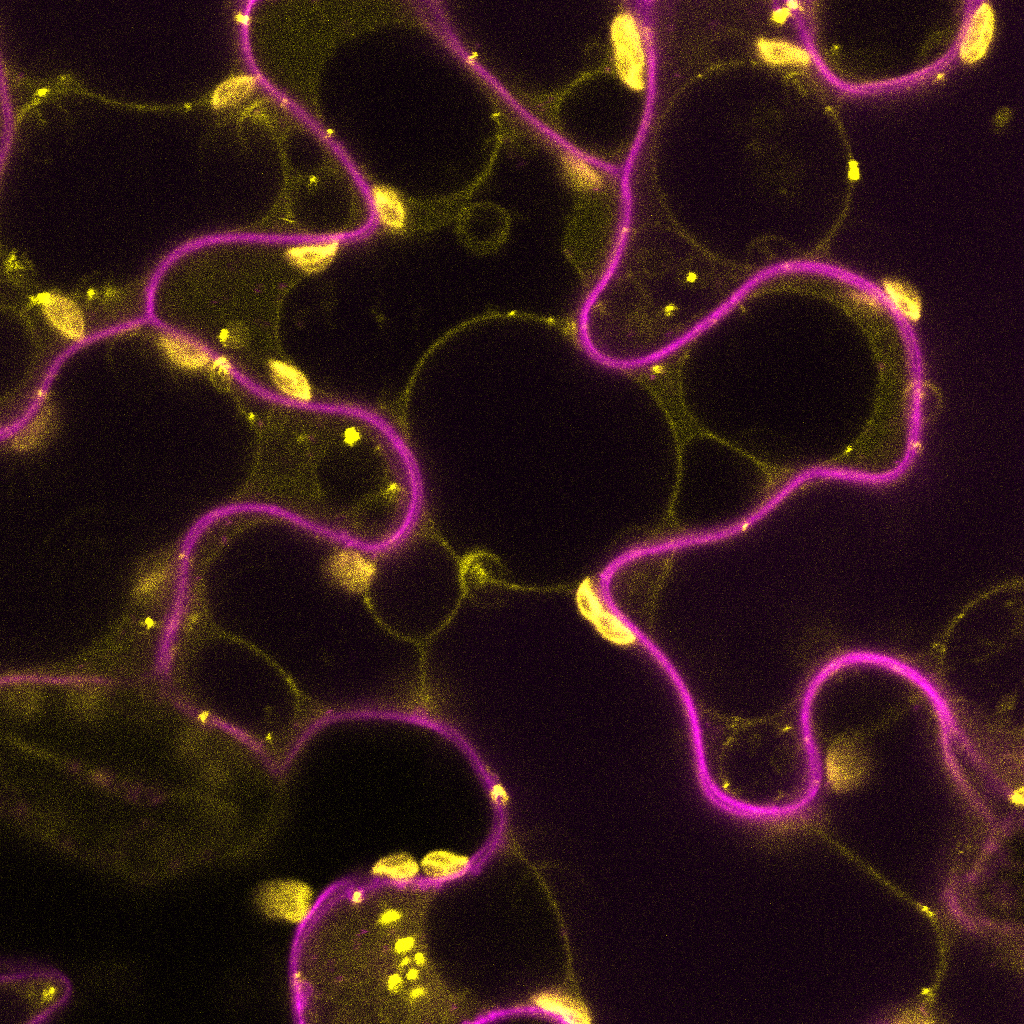

Supplement: Supplementary file 9 — Source data Fig. 4 [file 44319_2024_240_MOESM9_ESM.zip › Figure 4/Figure 4E/Fig(4-E)-PML5+PM.tiff]

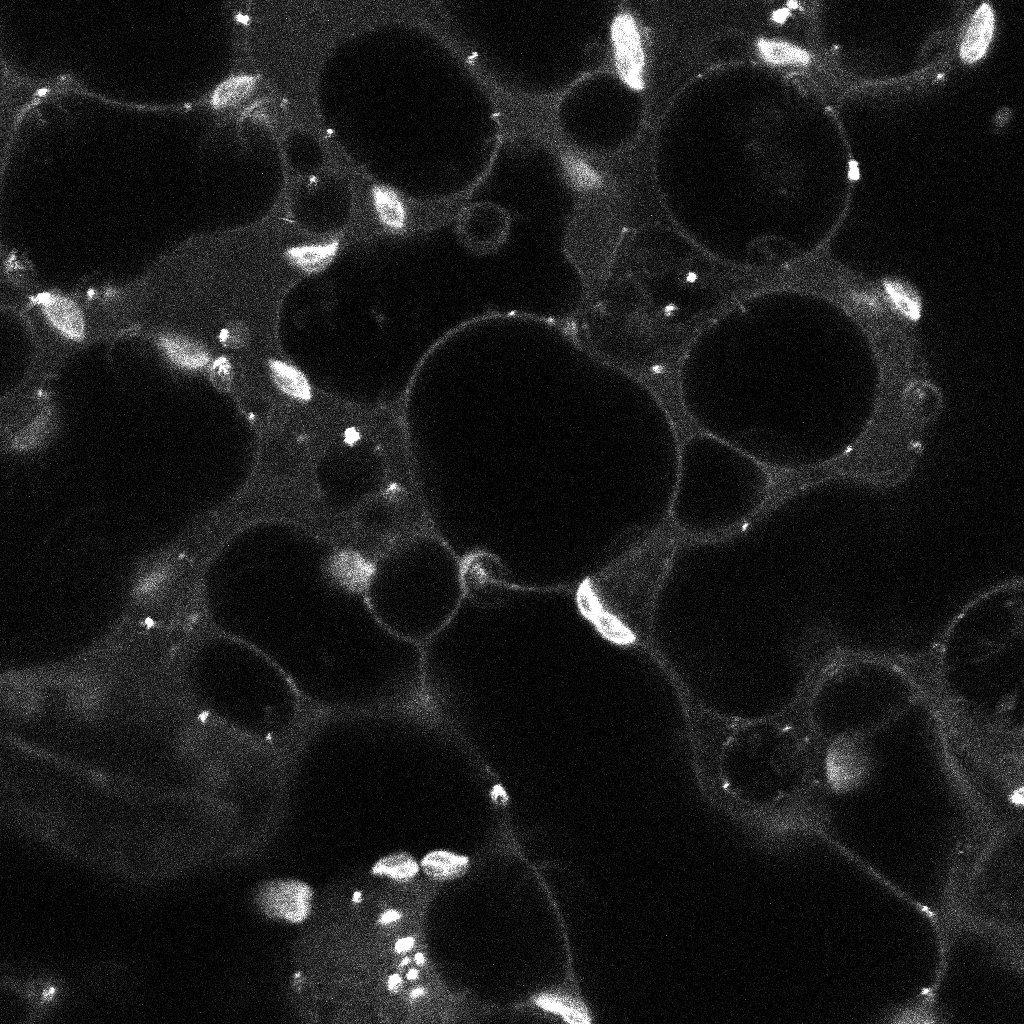

Supplement: Supplementary file 9 — Source data Fig. 4 [file 44319_2024_240_MOESM9_ESM.zip › Figure 4/Figure 4E/Fig(4-E)-PML5+PM.tiff_files/Fig(4-E)-PML5+PM_b0v0t0z0c0x0-1024y0-1024.tiff]

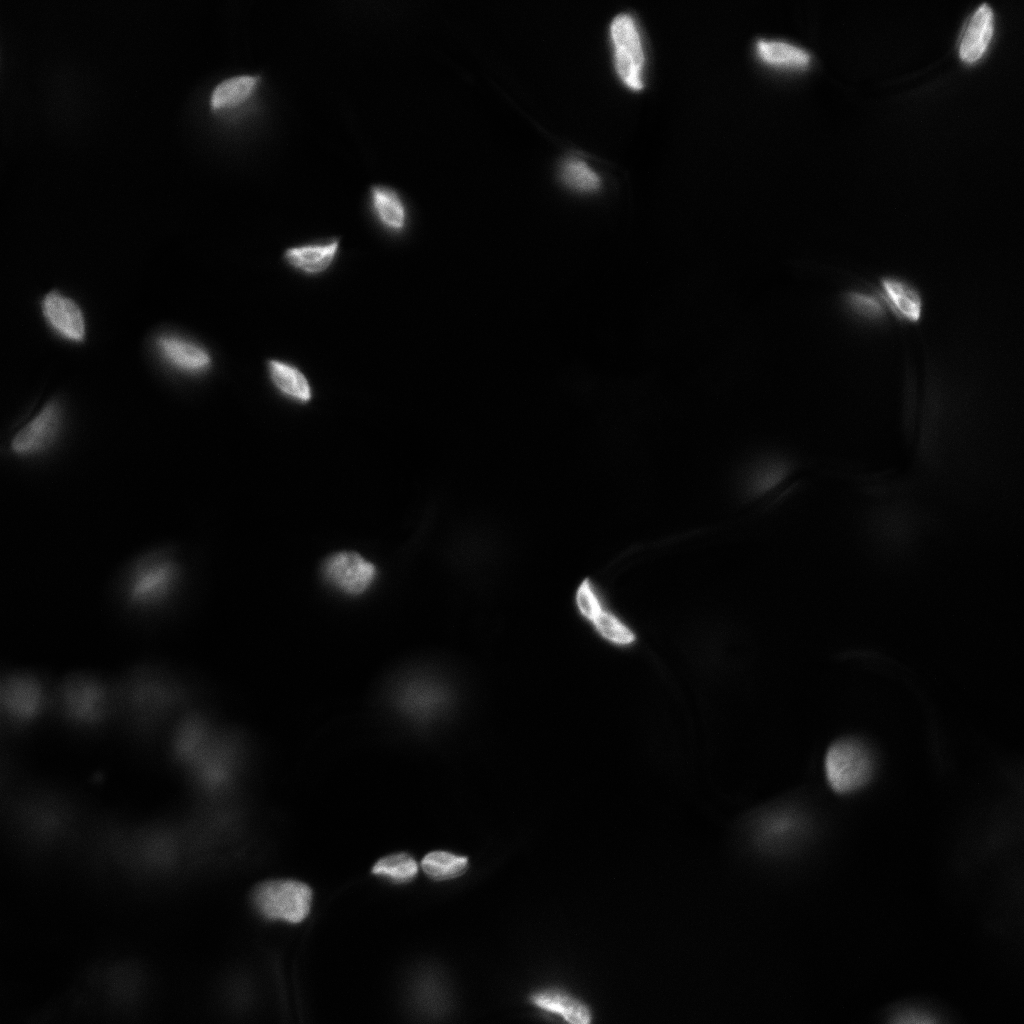

Supplement: Supplementary file 9 — Source data Fig. 4 [file 44319_2024_240_MOESM9_ESM.zip › Figure 4/Figure 4E/Fig(4-E)-PML5+PM.tiff_files/Fig(4-E)-PML5+PM_b0v0t0z0c1x0-1024y0-1024.tiff]

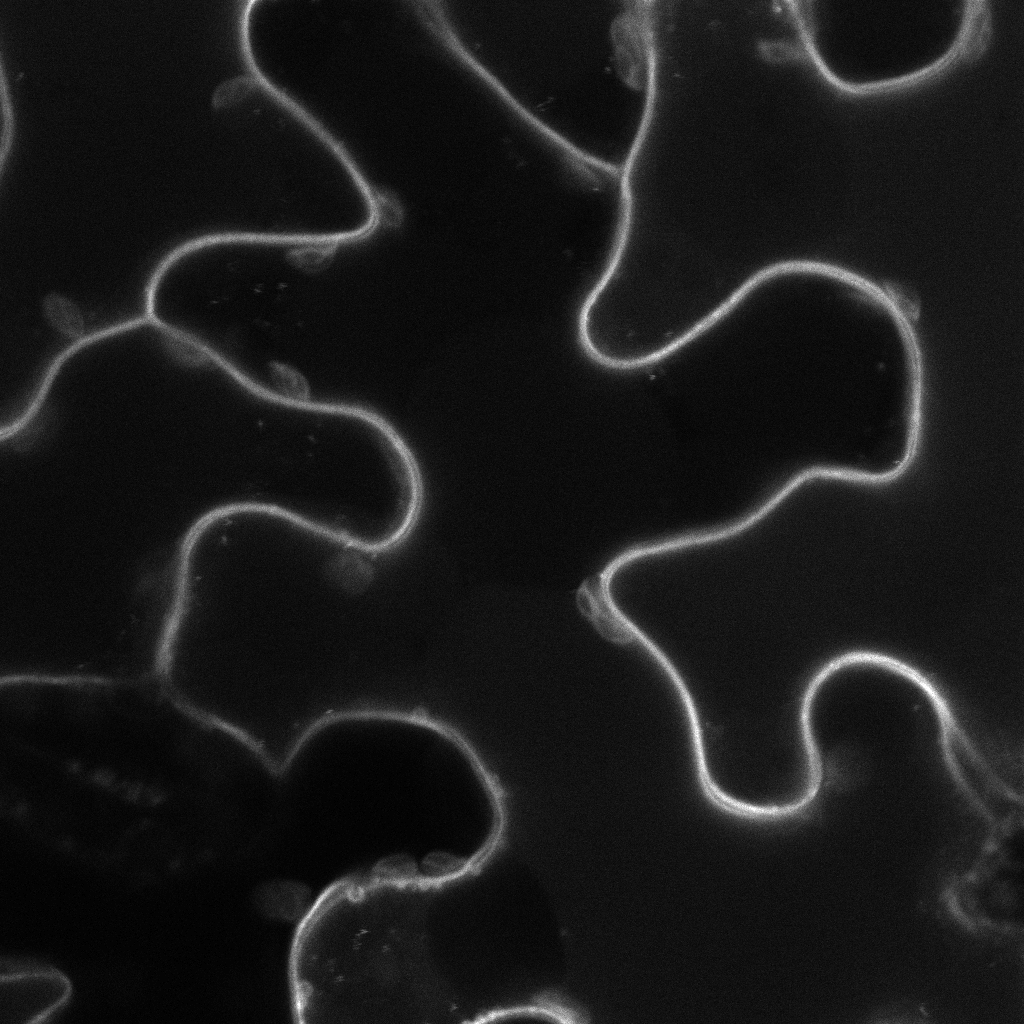

Supplement: Supplementary file 9 — Source data Fig. 4 [file 44319_2024_240_MOESM9_ESM.zip › Figure 4/Figure 4E/Fig(4-E)-PML5+PM.tiff_files/Fig(4-E)-PML5+PM_b0v0t0z0c2x0-1024y0-1024.tiff]

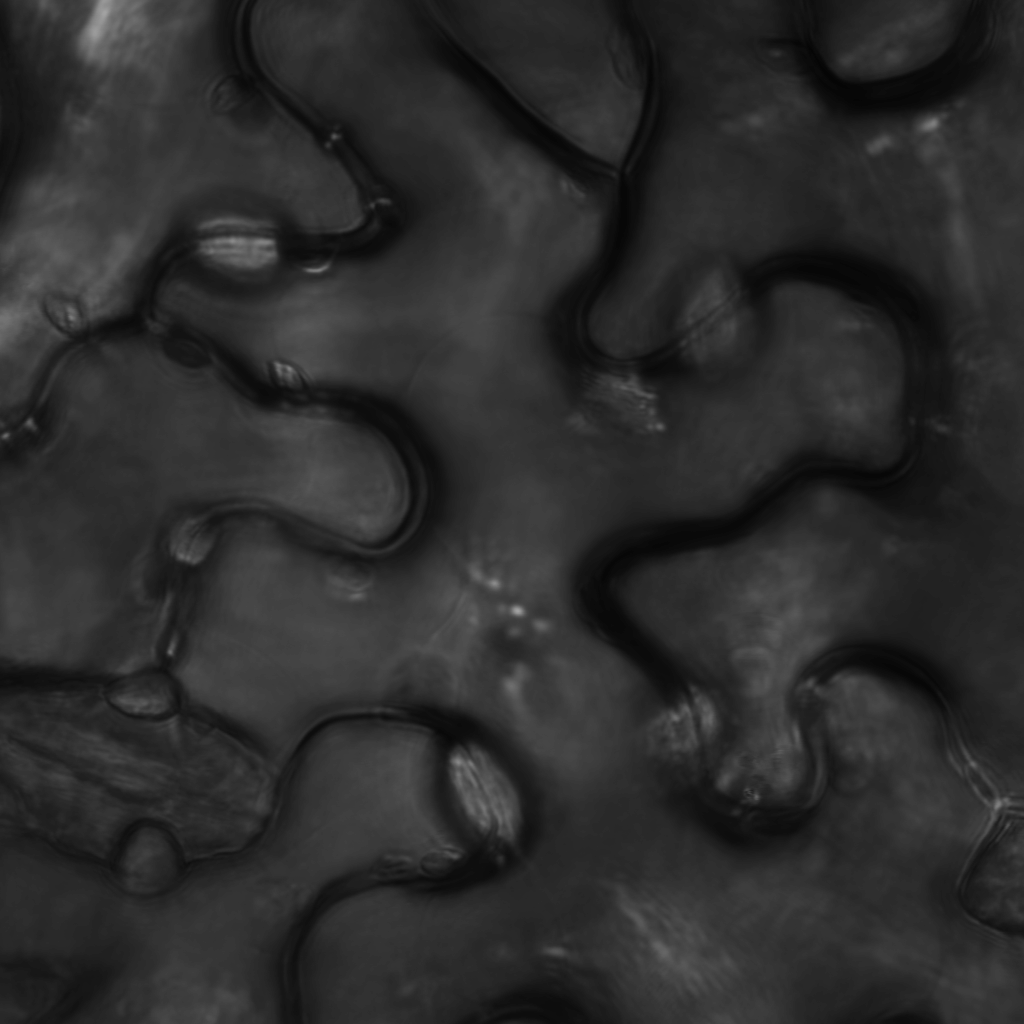

Supplement: Supplementary file 9 — Source data Fig. 4 [file 44319_2024_240_MOESM9_ESM.zip › Figure 4/Figure 4E/Fig(4-E)-PML5+PM.tiff_files/Fig(4-E)-PML5+PM_b0v0t0z0c3x0-1024y0-1024.tiff]

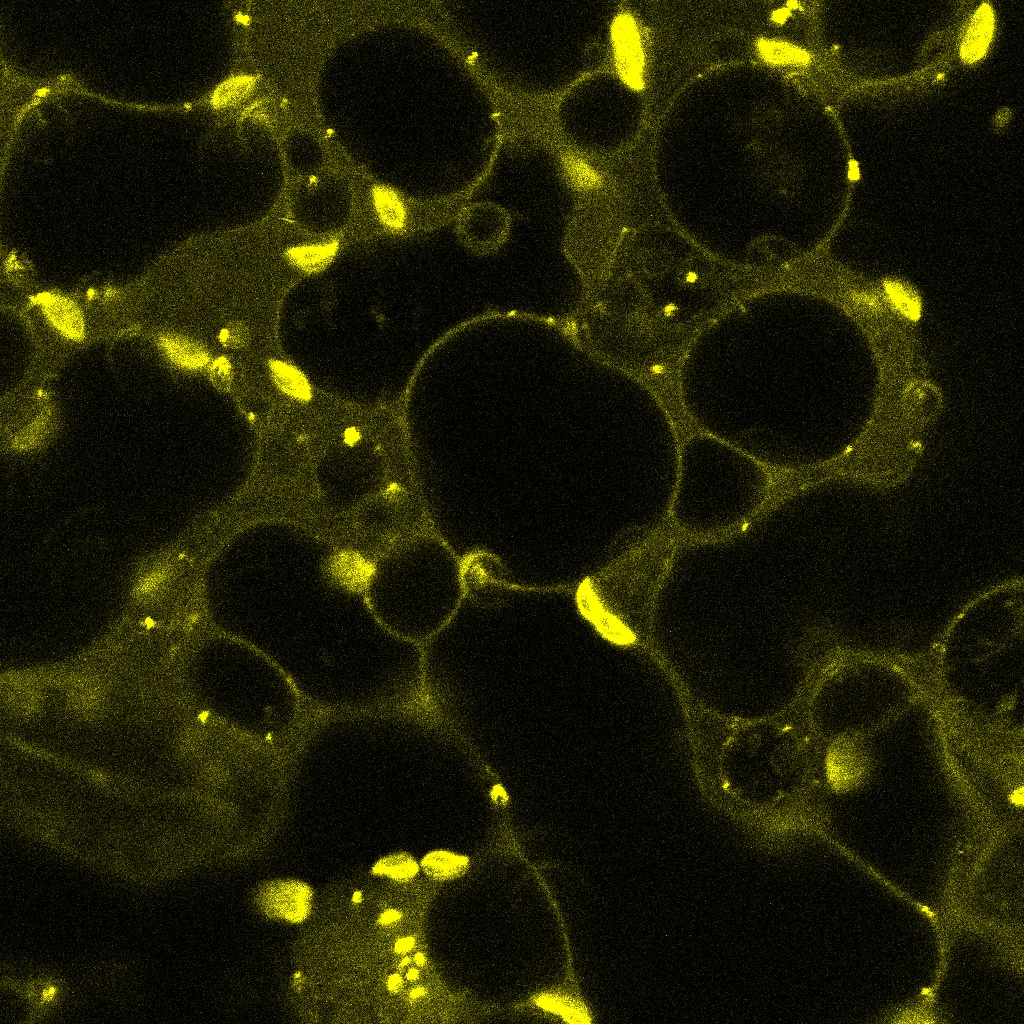

Supplement: Supplementary file 9 — Source data Fig. 4 [file 44319_2024_240_MOESM9_ESM.zip › Figure 4/Figure 4E/Fig(4-E)-PML5+PM/Image 28_c1.jpg]

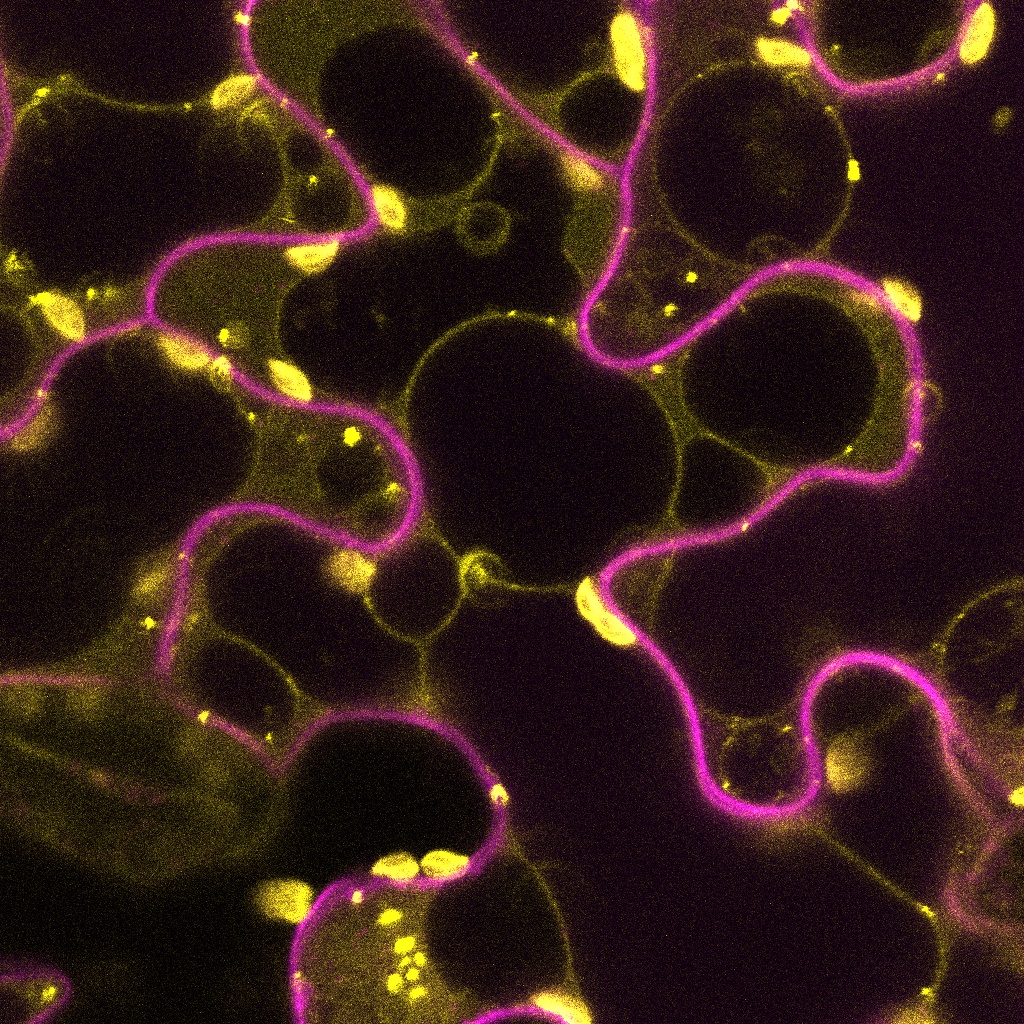

Supplement: Supplementary file 9 — Source data Fig. 4 [file 44319_2024_240_MOESM9_ESM.zip › Figure 4/Figure 4E/Fig(4-E)-PML5+PM/Image 28_c1-4.jpg]

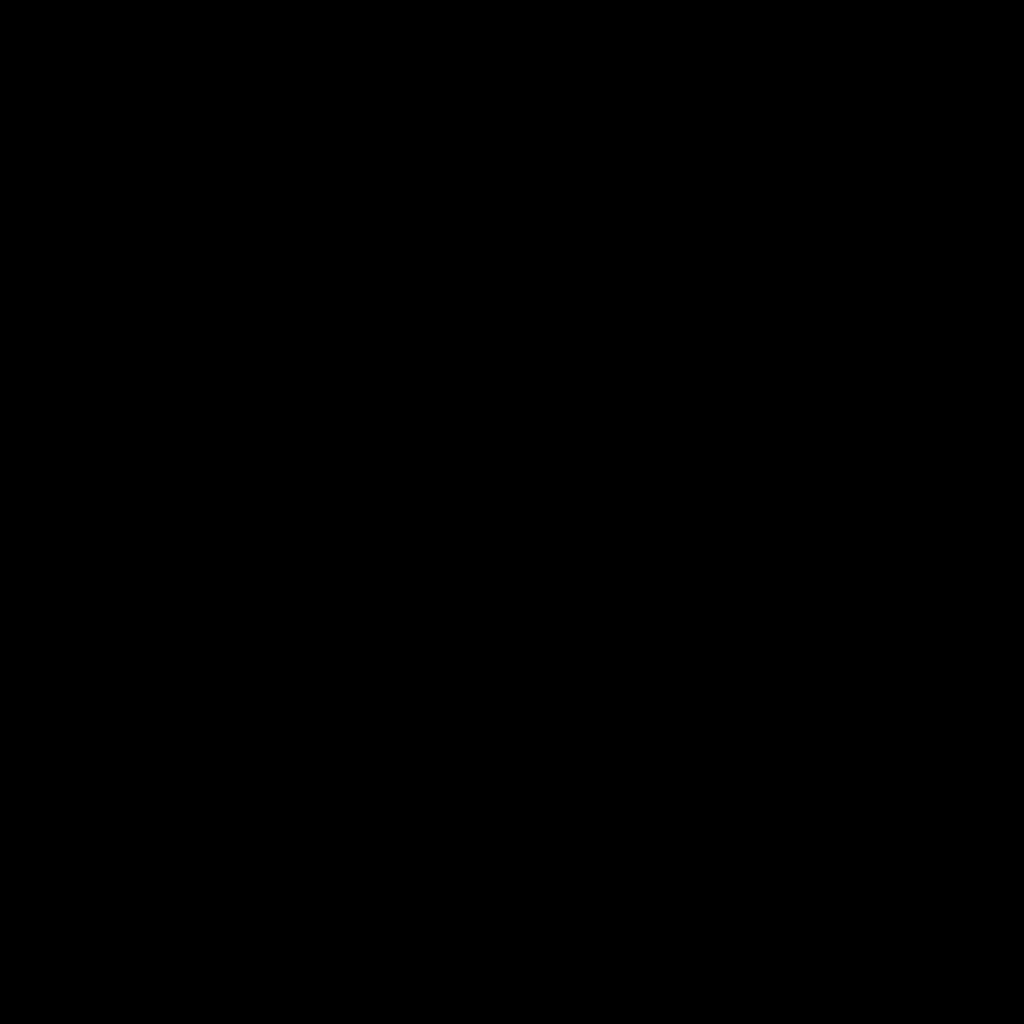

Supplement: Supplementary file 9 — Source data Fig. 4 [file 44319_2024_240_MOESM9_ESM.zip › Figure 4/Figure 4E/Fig(4-E)-PML5+PM/Image 28_c2.jpg]

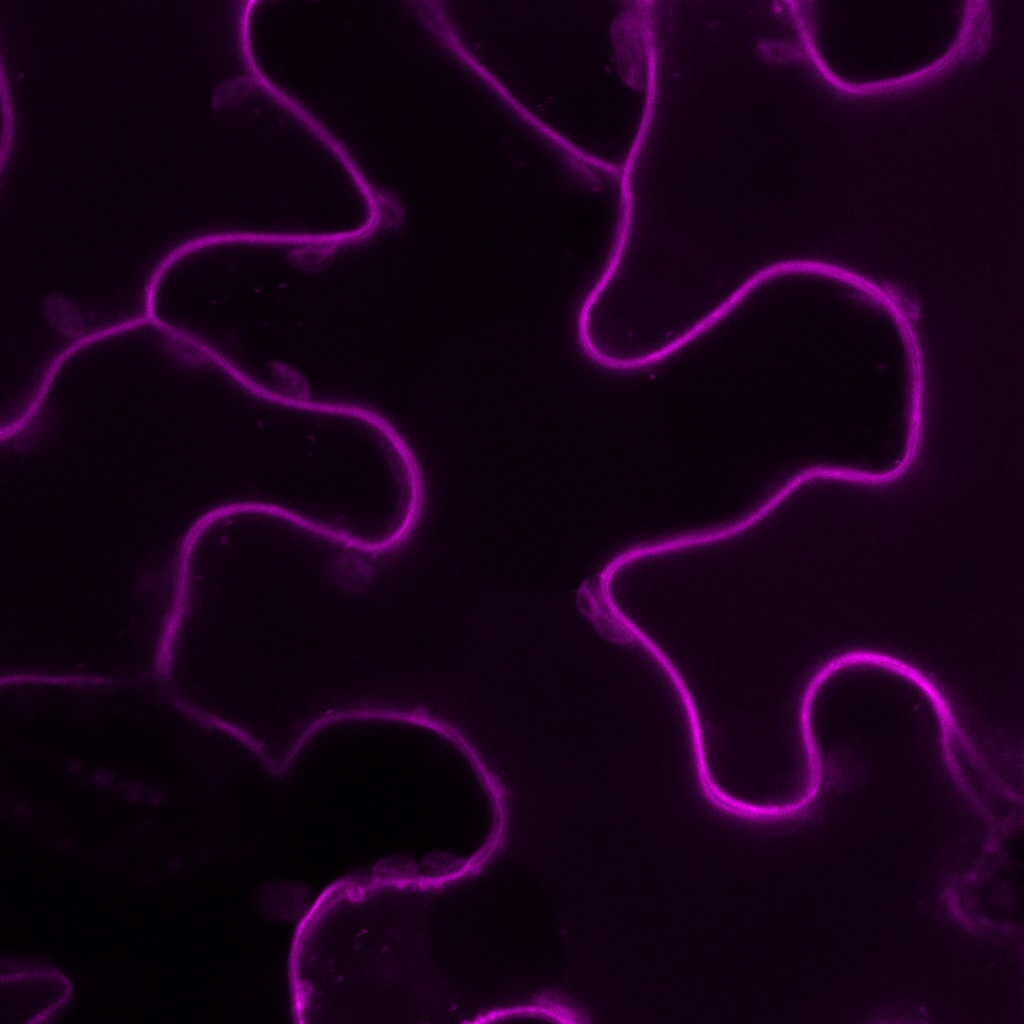

Supplement: Supplementary file 9 — Source data Fig. 4 [file 44319_2024_240_MOESM9_ESM.zip › Figure 4/Figure 4E/Fig(4-E)-PML5+PM/Image 28_c3.jpg]

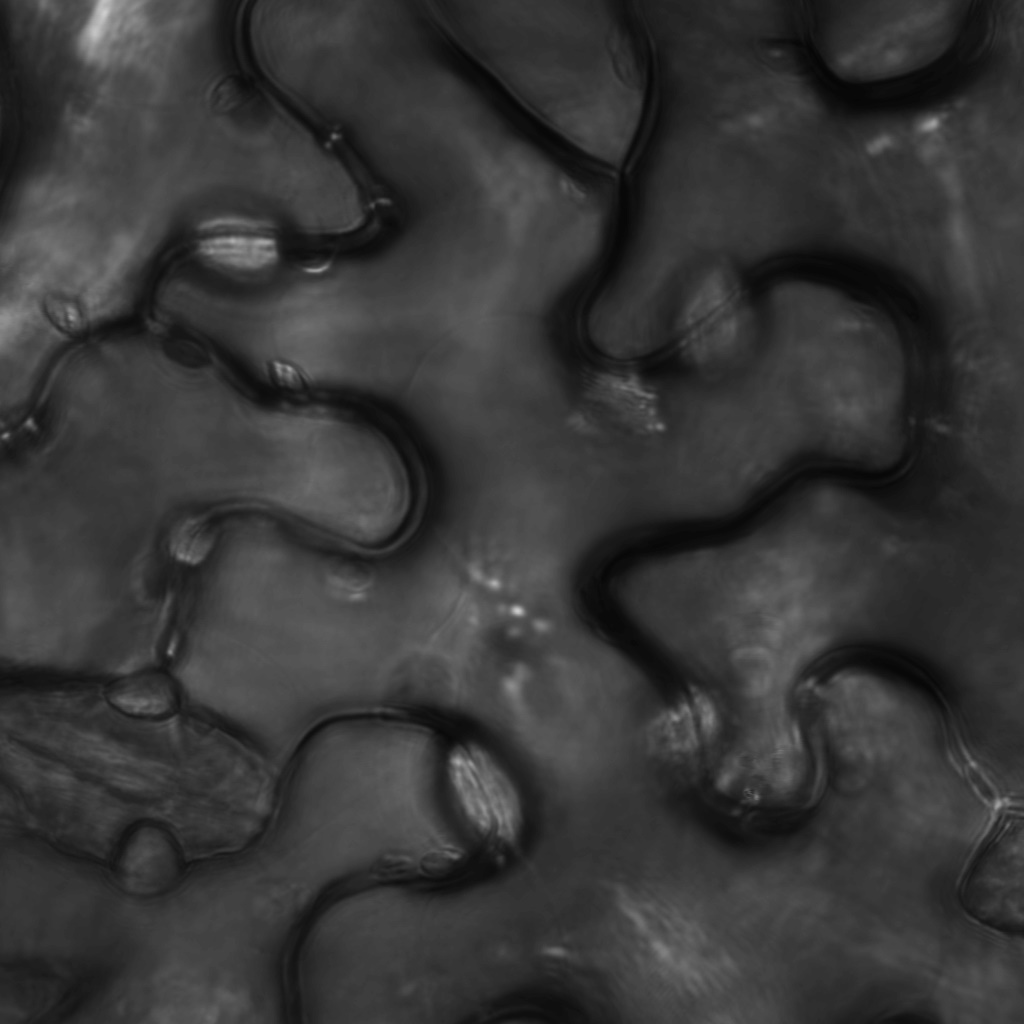

Supplement: Supplementary file 9 — Source data Fig. 4 [file 44319_2024_240_MOESM9_ESM.zip › Figure 4/Figure 4E/Fig(4-E)-PML5+PM/Image 28_c4.jpg]

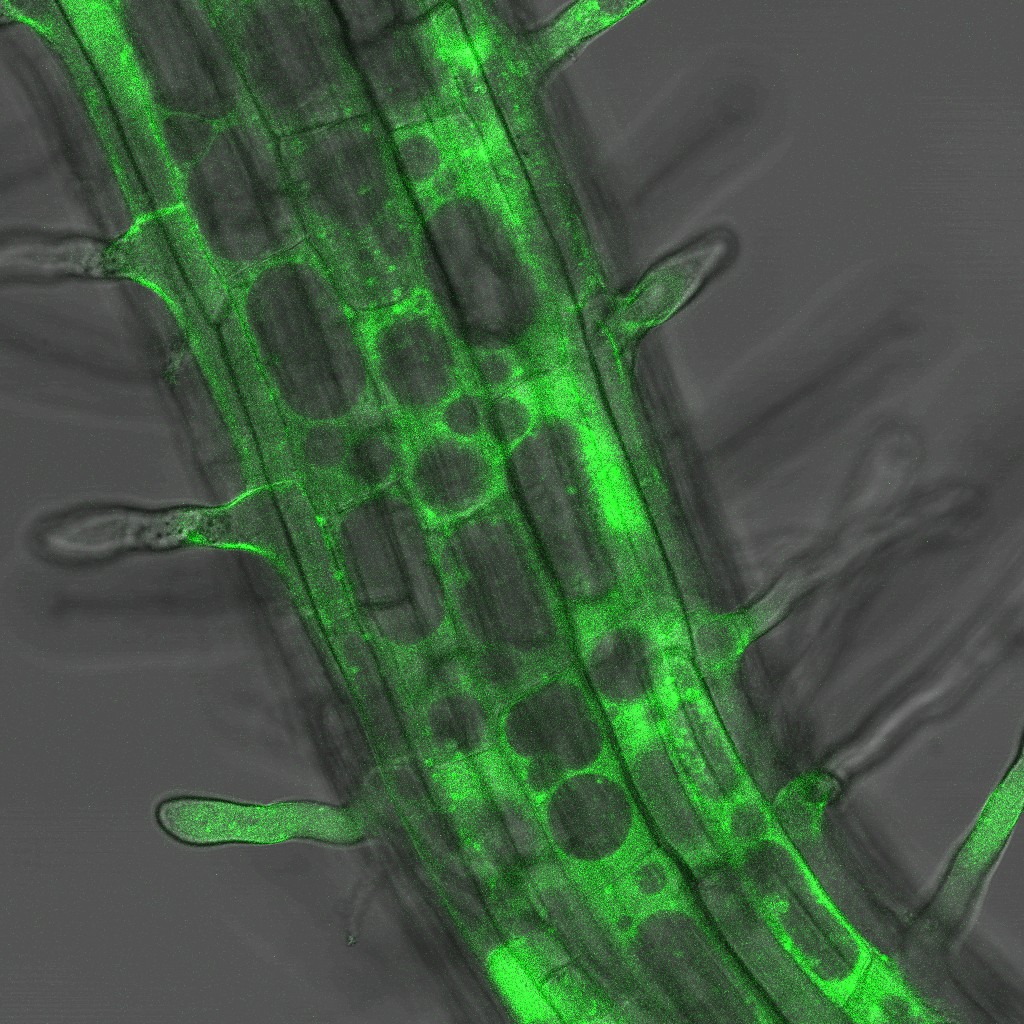

Supplement: Supplementary file 9 — Source data Fig. 4 [file 44319_2024_240_MOESM9_ESM.zip › Figure 4/Figure 4F/Fig(4-F)-PML5 Col-0 Lines.tiff]

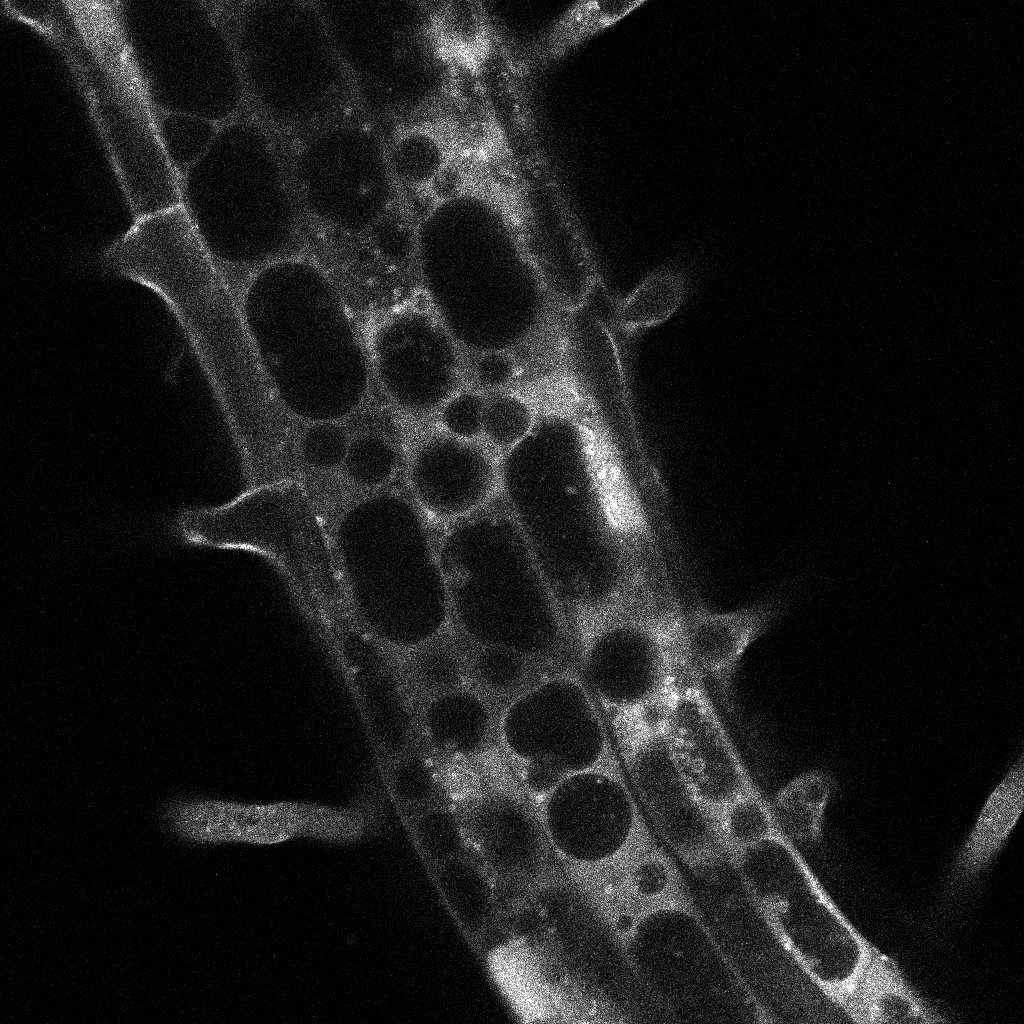

Supplement: Supplementary file 9 — Source data Fig. 4 [file 44319_2024_240_MOESM9_ESM.zip › Figure 4/Figure 4F/Fig(4-F)-PML5 Col-0 Lines.tiff_files/Fig(4-F)-PML5 Col-0 Lines_b0v0t0z0c0x0-1024y0-1024m0.tiff]

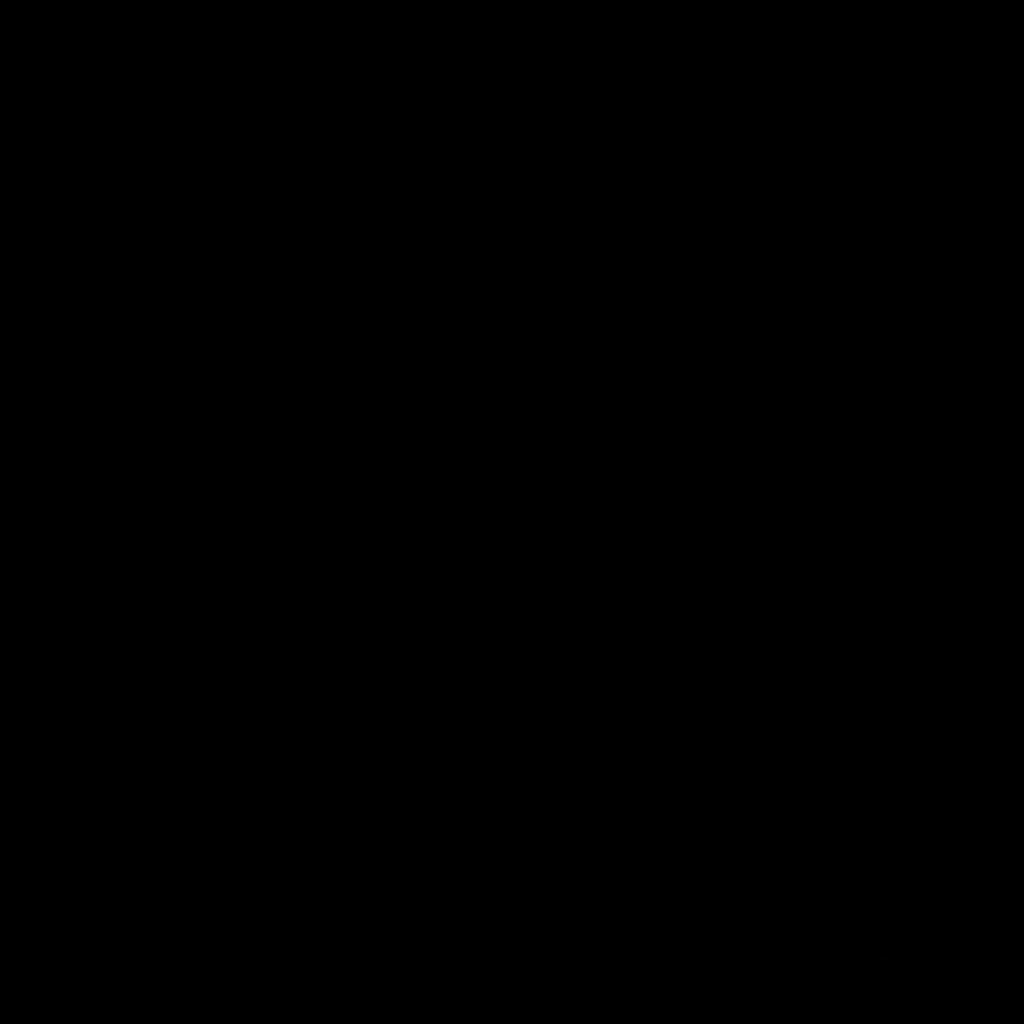

Supplement: Supplementary file 9 — Source data Fig. 4 [file 44319_2024_240_MOESM9_ESM.zip › Figure 4/Figure 4F/Fig(4-F)-PML5 Col-0 Lines.tiff_files/Fig(4-F)-PML5 Col-0 Lines_b0v0t0z0c1x0-1024y0-1024m0.tiff]

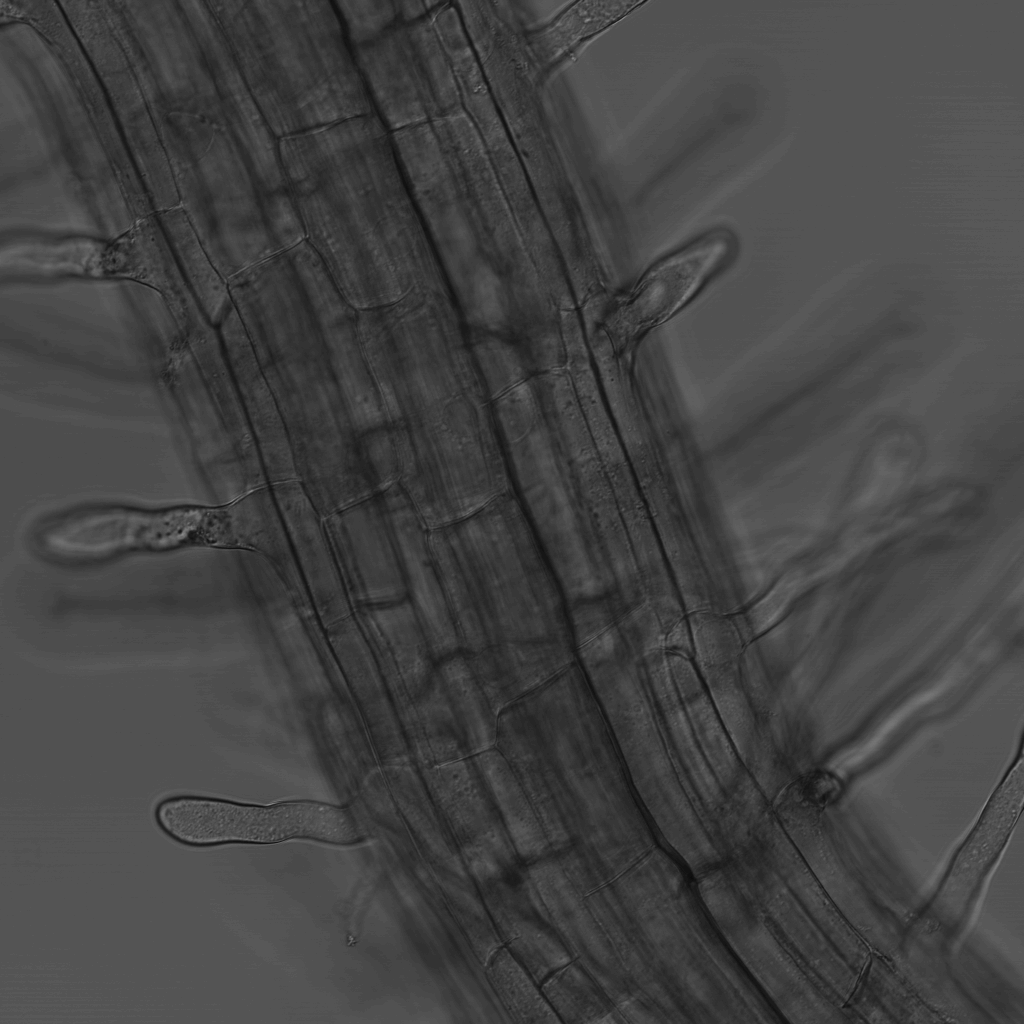

Supplement: Supplementary file 9 — Source data Fig. 4 [file 44319_2024_240_MOESM9_ESM.zip › Figure 4/Figure 4F/Fig(4-F)-PML5 Col-0 Lines.tiff_files/Fig(4-F)-PML5 Col-0 Lines_b0v0t0z0c2x0-1024y0-1024m0.tiff]

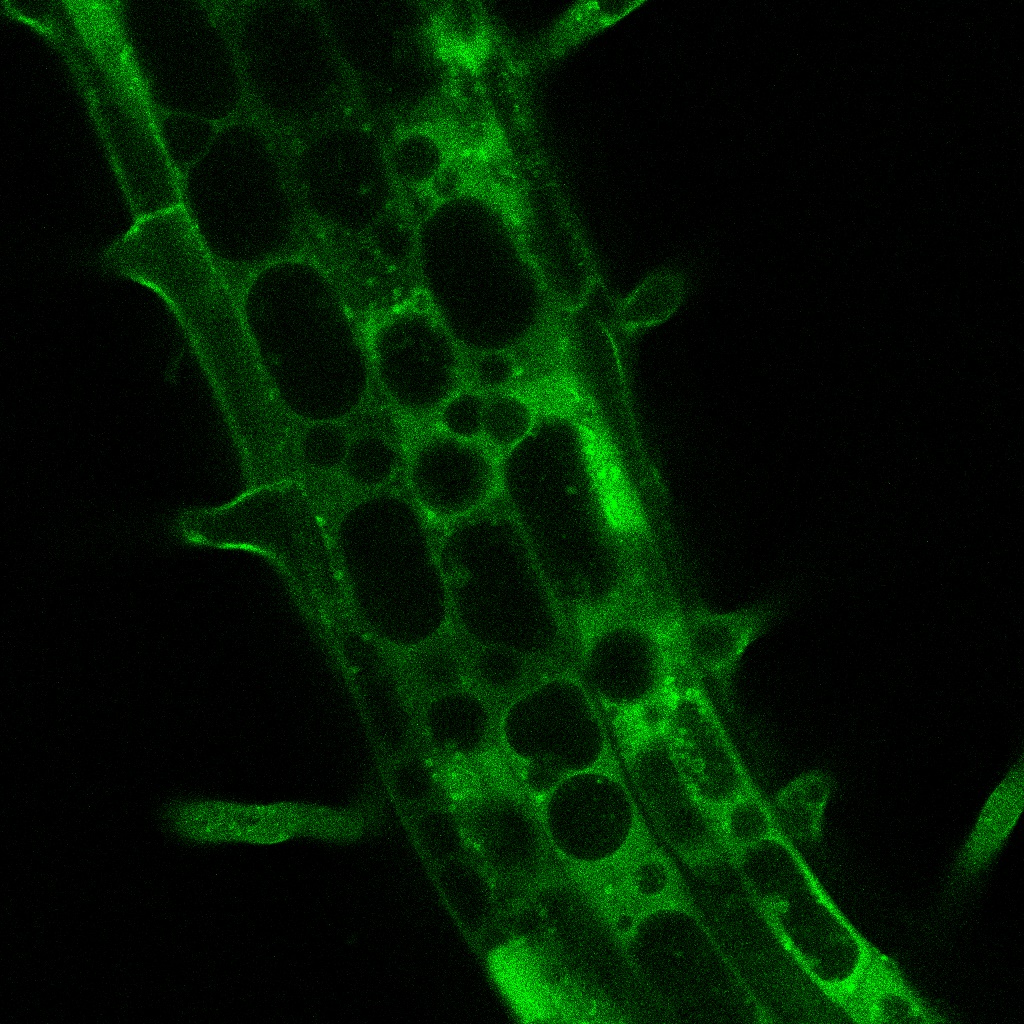

Supplement: Supplementary file 9 — Source data Fig. 4 [file 44319_2024_240_MOESM9_ESM.zip › Figure 4/Figure 4F/Fig(4-F)-PML5 Col-0 Lines/Image 14_c1.jpg]

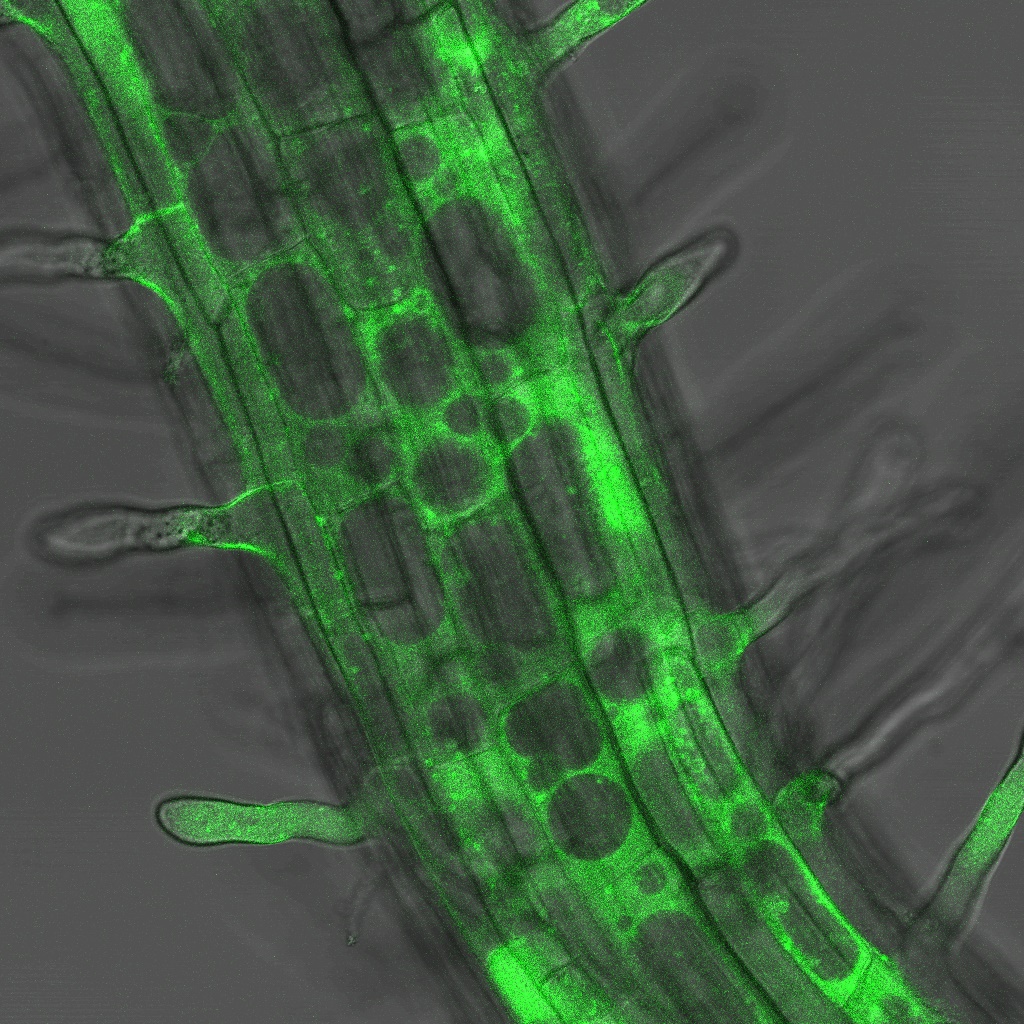

Supplement: Supplementary file 9 — Source data Fig. 4 [file 44319_2024_240_MOESM9_ESM.zip › Figure 4/Figure 4F/Fig(4-F)-PML5 Col-0 Lines/Image 14_c1-3.jpg]

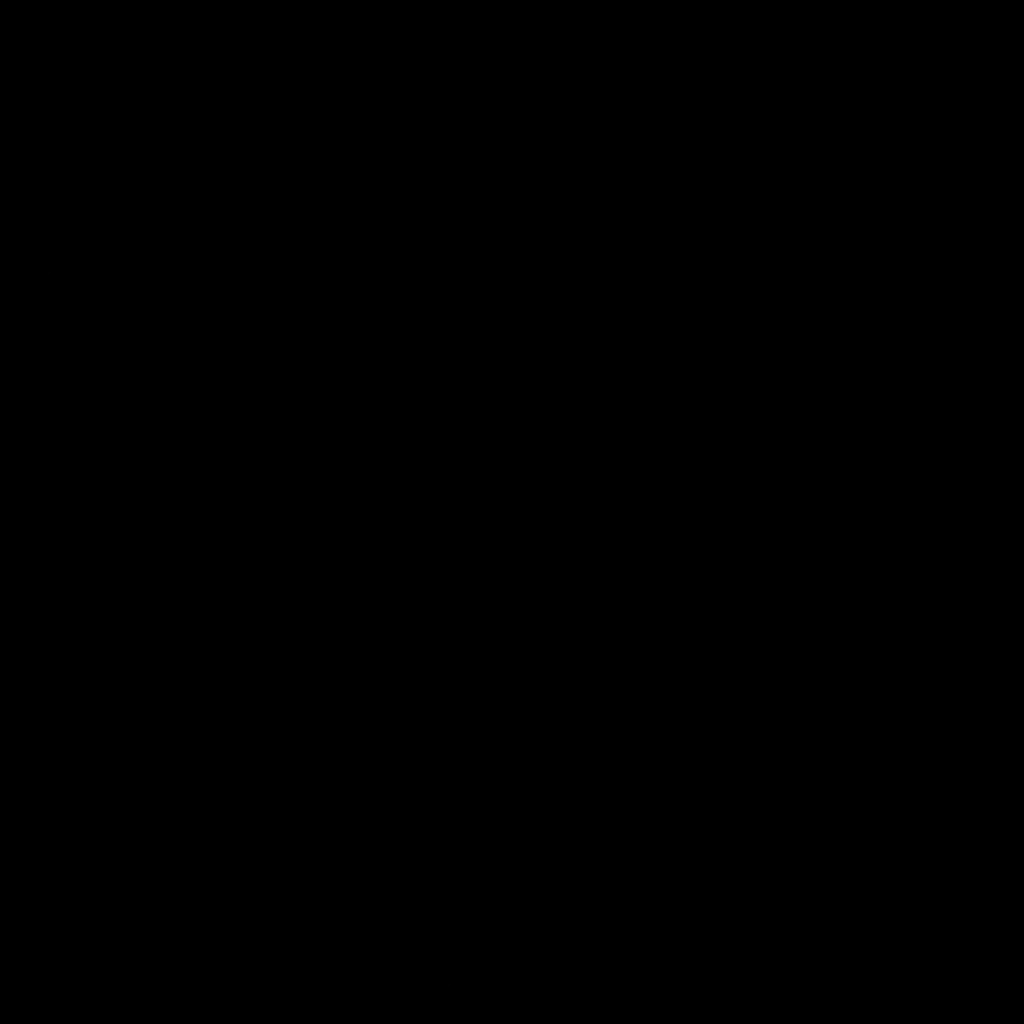

Supplement: Supplementary file 9 — Source data Fig. 4 [file 44319_2024_240_MOESM9_ESM.zip › Figure 4/Figure 4F/Fig(4-F)-PML5 Col-0 Lines/Image 14_c2.jpg]

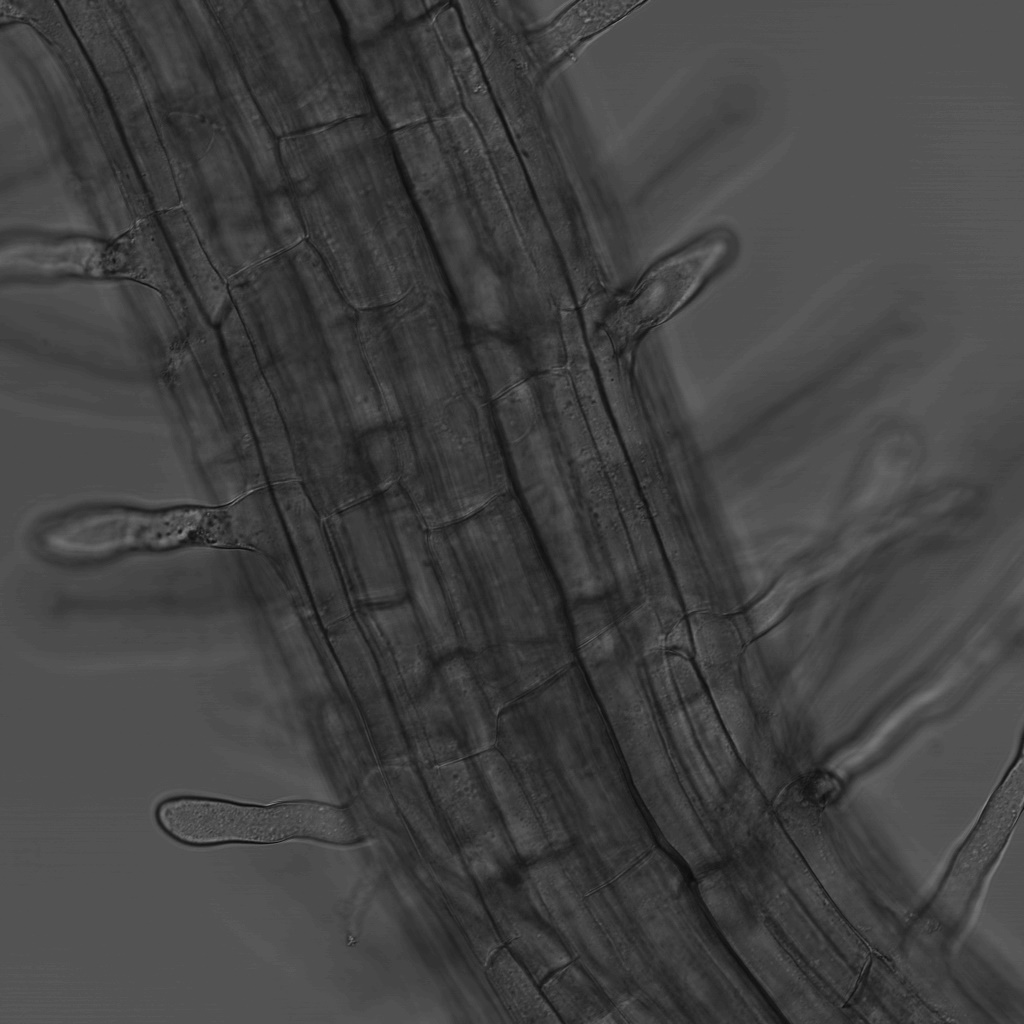

Supplement: Supplementary file 9 — Source data Fig. 4 [file 44319_2024_240_MOESM9_ESM.zip › Figure 4/Figure 4F/Fig(4-F)-PML5 Col-0 Lines/Image 14_c3.jpg]

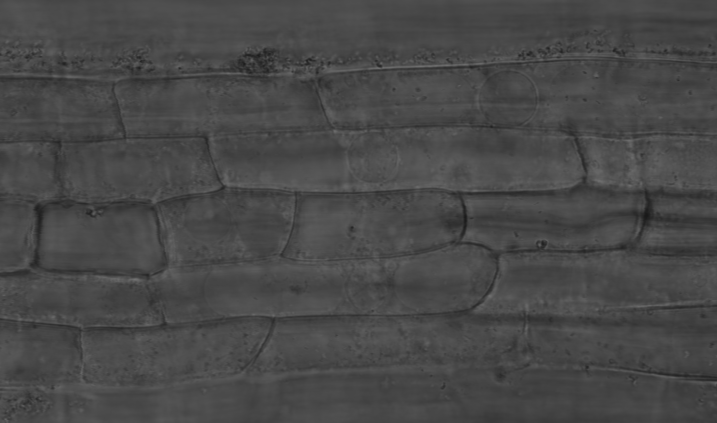

Supplement: Supplementary file 9 — Source data Fig. 4 [file 44319_2024_240_MOESM9_ESM.zip › Figure 4/Figure 4G/Black white/Bright Field.tif]

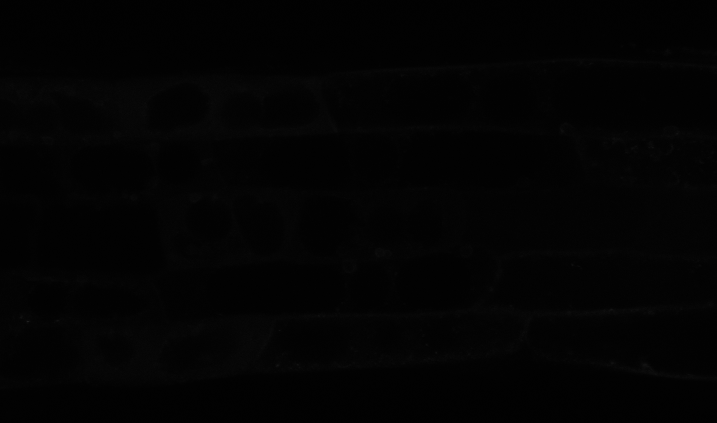

Supplement: Supplementary file 9 — Source data Fig. 4 [file 44319_2024_240_MOESM9_ESM.zip › Figure 4/Figure 4G/Black white/GFP (PML5-GFP).tif]

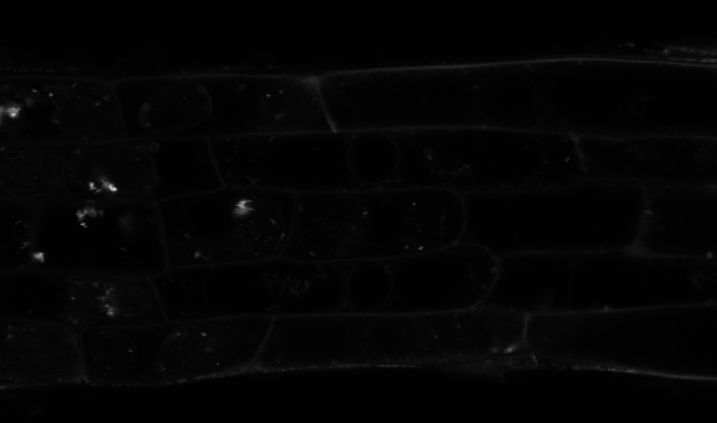

Supplement: Supplementary file 9 — Source data Fig. 4 [file 44319_2024_240_MOESM9_ESM.zip › Figure 4/Figure 4G/Black white/RFP (CBL6-mCherry).tif]

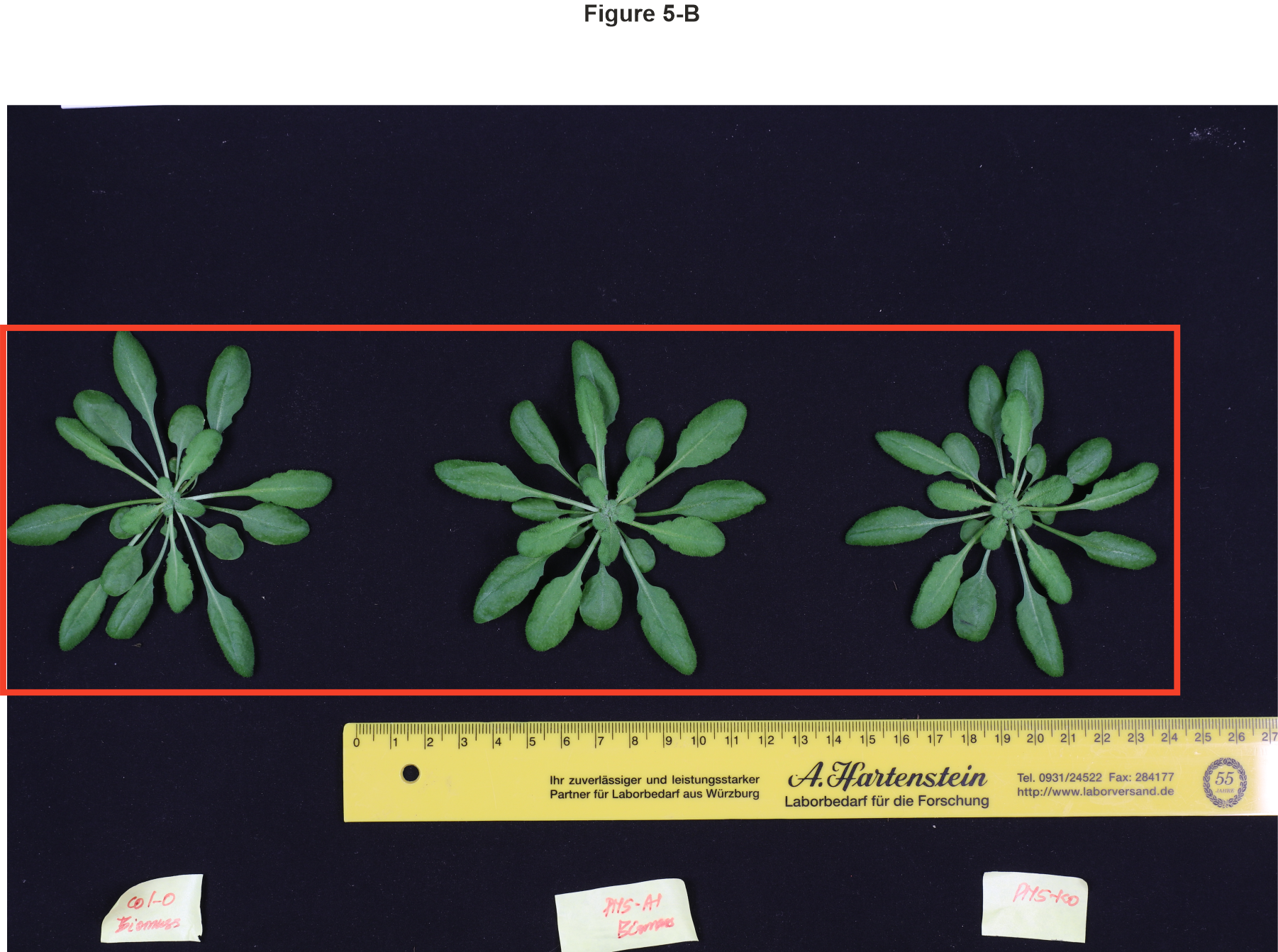

Supplement: Supplementary file 10 — Source data Fig. 5 [file 44319_2024_240_MOESM10_ESM.zip › Figure 5/5B/Fig 5B.png]

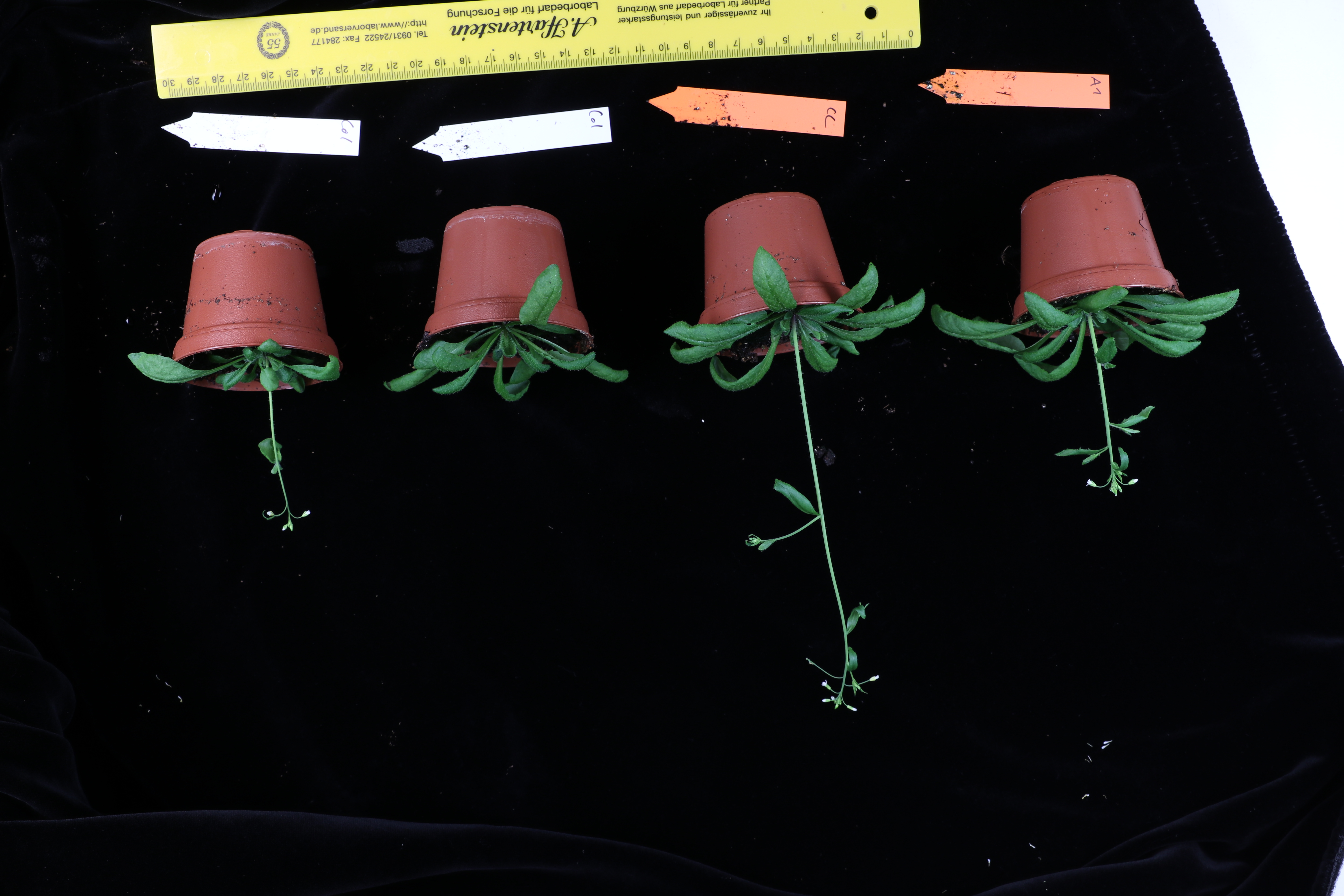

Supplement: Supplementary file 10 — Source data Fig. 5 [file 44319_2024_240_MOESM10_ESM.zip › Figure 5/5D/Fig 5D.JPG]

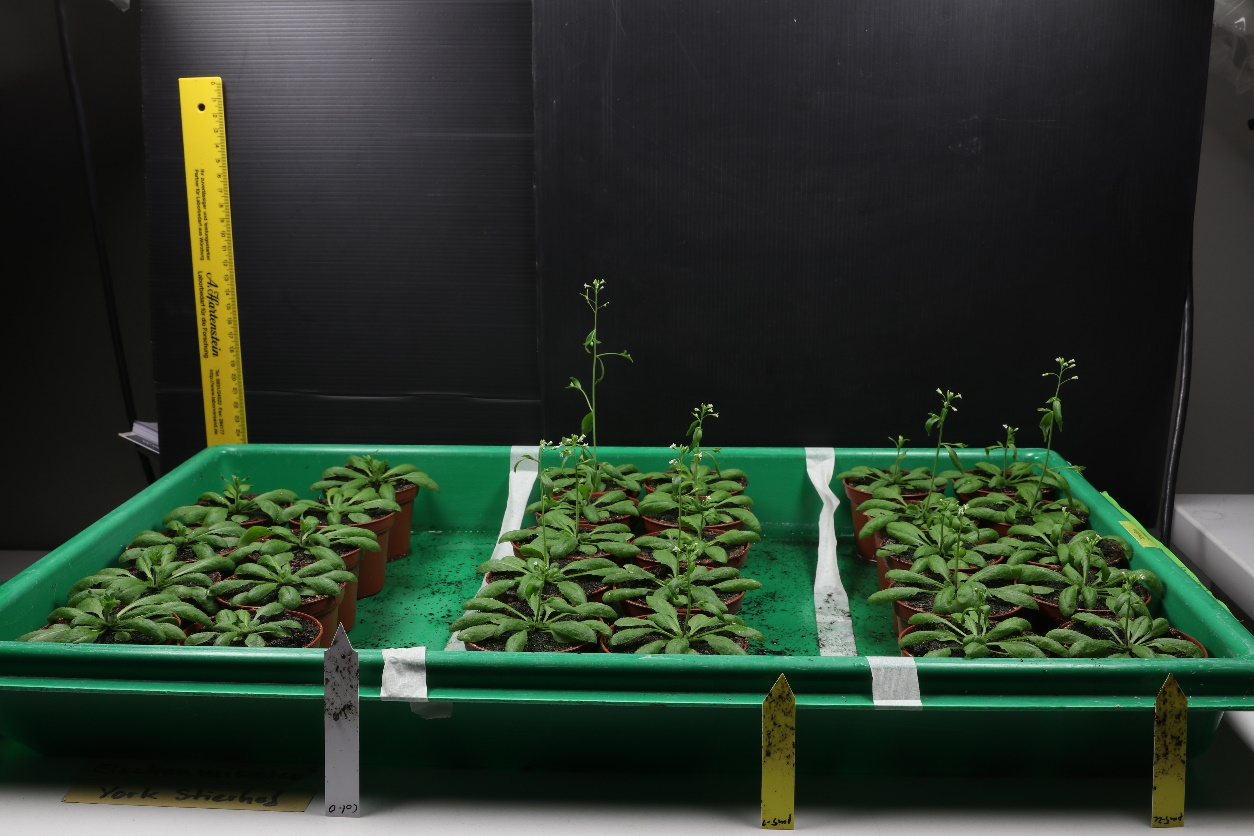


*Col-0 pml5-1 pml5-2c*

Supplement: Supplementary file 10 — Source data Fig. 5 [file 44319_2024_240_MOESM10_ESM.zip › Figure 5/5D/Fig 5D-2.docx]

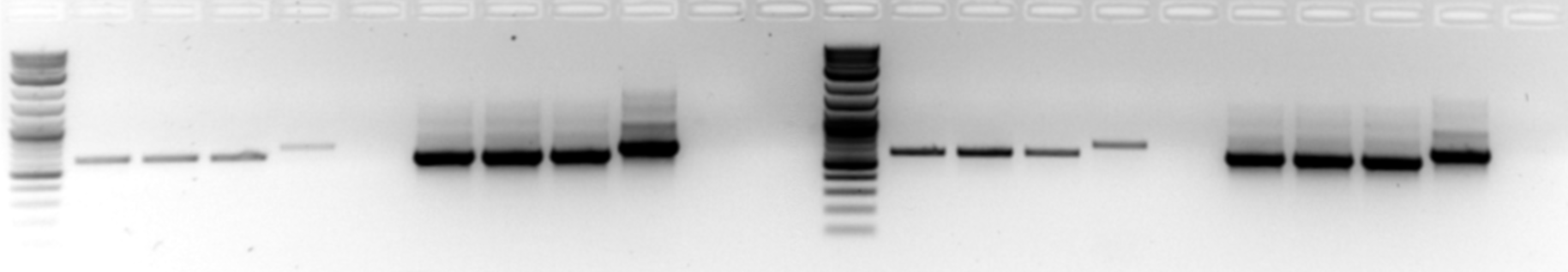


cDNA_*ACTIN*

RNA_*PML5*

cDNA_*PML5*


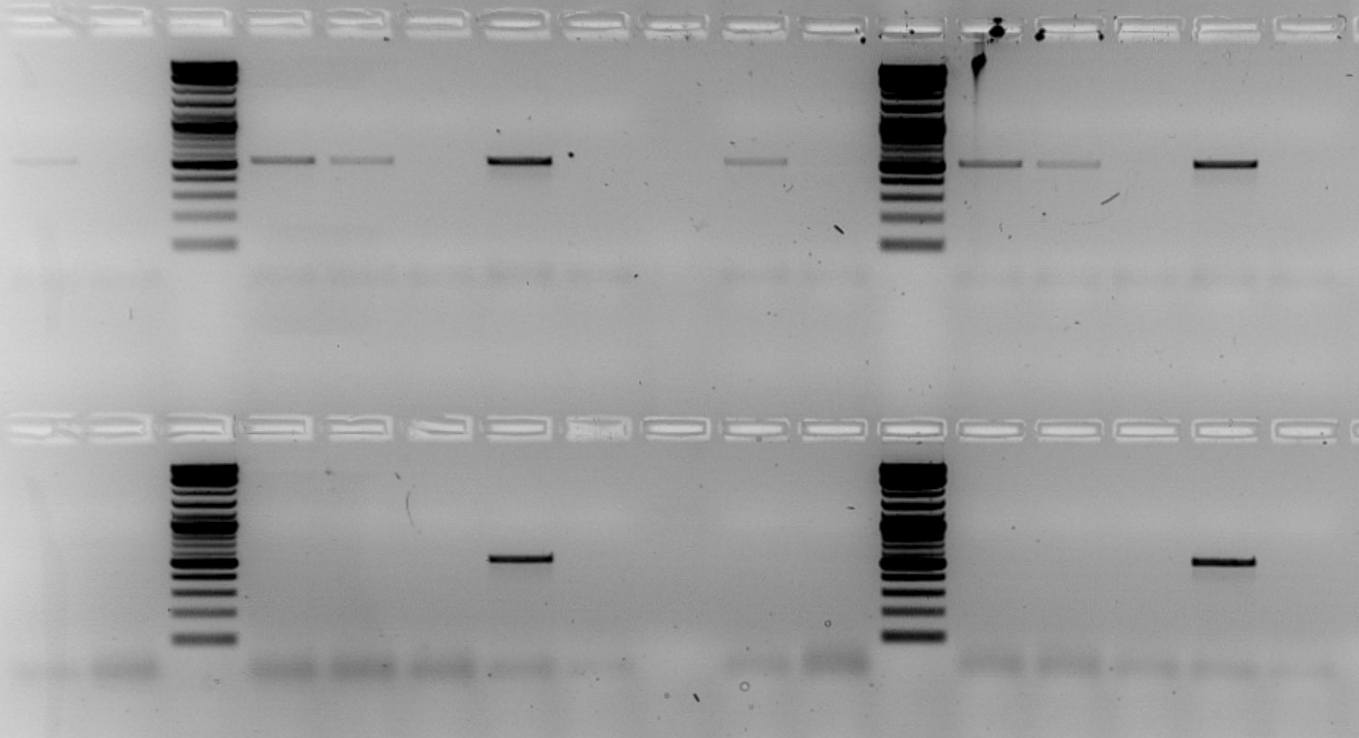

Supplement: Supplementary file 10 — Source data Fig. 5 [file 44319_2024_240_MOESM10_ESM.zip › Figure 5/5E/Fig 5E.docx]

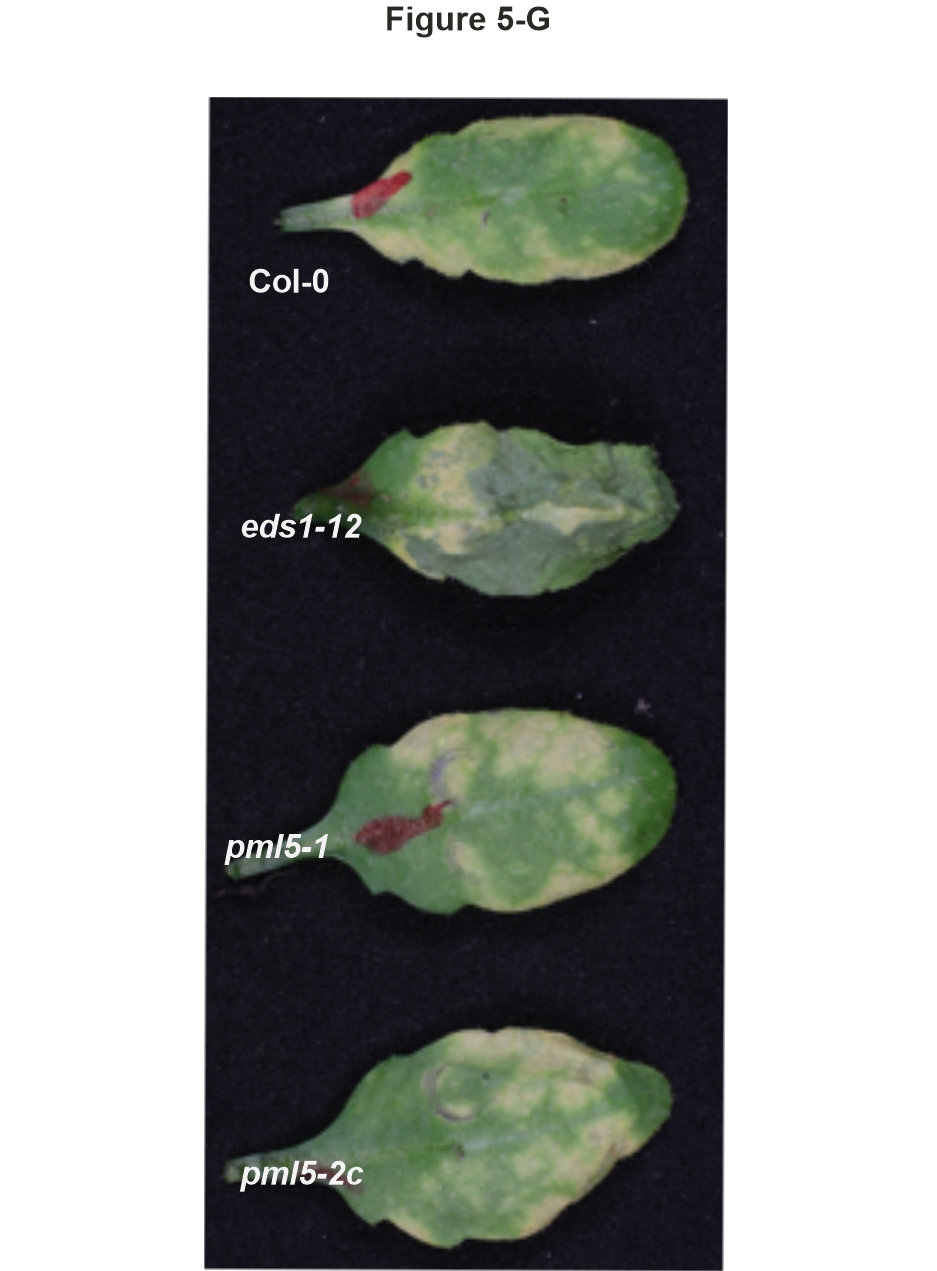

Supplement: Supplementary file 10 — Source data Fig. 5 [file 44319_2024_240_MOESM10_ESM.zip › Figure 5/5G/Fig 5G.png]
