# Supplementary material for: Activation of an atypical plant NLR with an N-terminal deletion initiates cell death at the vacuole
Source: EMBO Rep. 2024 Sep 6;25(10):19. doi: 10.1038/s44319-024-00240-4 (PMC11467418; doi:10.1038/s44319-024-00240-4)
Supplement: Supplementary file 11 — Expanded View Figures [file 44319_2024_240_MOESM11_ESM.pdf]

Expanded View Figures

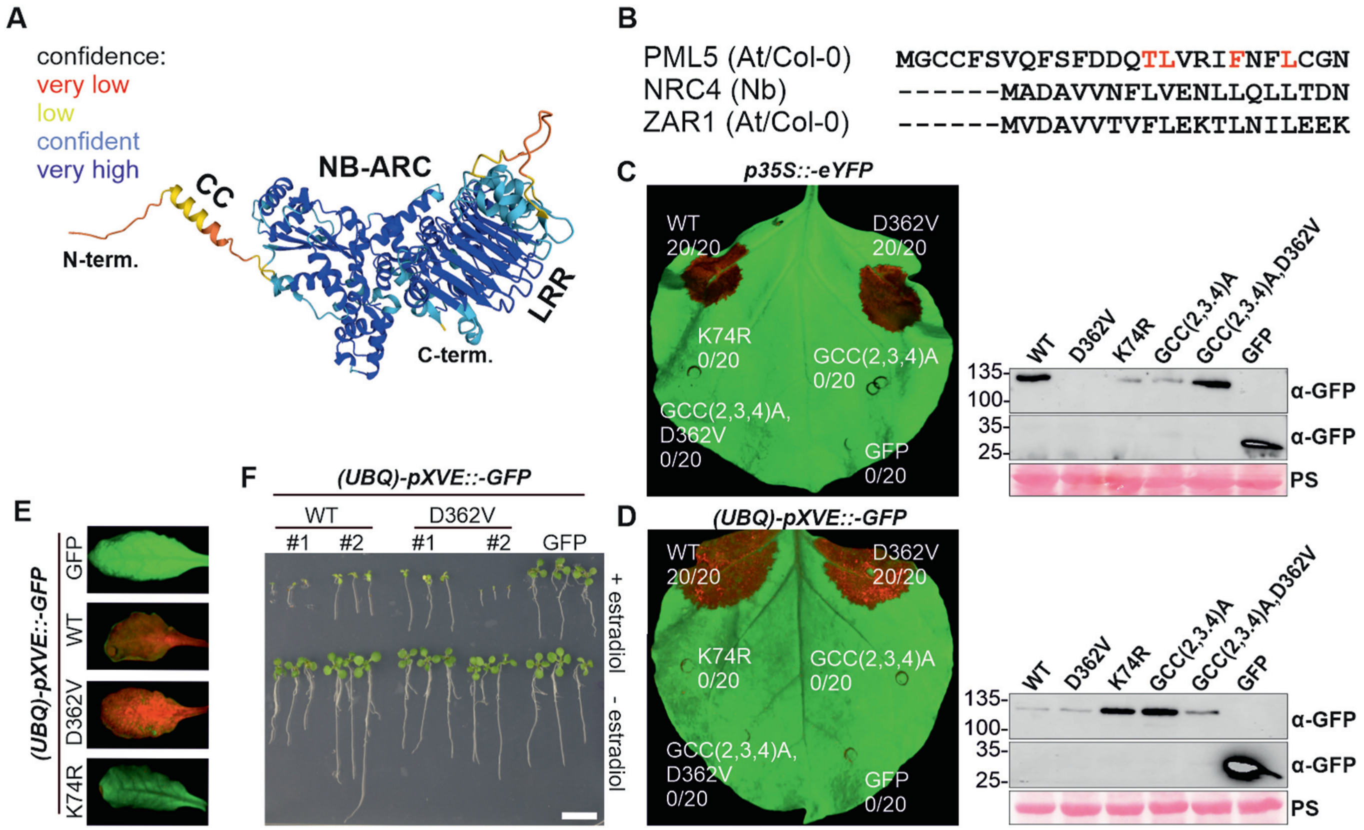

Figure EV1. PML5 functions as a canonical cell death inducing NLR.

(**A**) AlphaFold 2 structural prediction of PML5. CC, NB-ARC, and LRR domains are indicated. A potential alpha 1 helix is predicted between Asp/D13 and Ile/I 27. (**B**) Sequence alignment of the first 26 N-terminal amino acids of Col-0 PML5, ZAR1 and *N. benthamiana* (Nb) NRC4, highlighting the hydrophobic and polar uncharged amino acids mutated in PML5 (see Fig. 1D). (**C**, **D**) Cell death induced by transiently expressed wild-type PML5 and mutant variants (**C**) constitutively under 35S promoter (top left) or conditionally expressed under a 35S promoter-controlled estradiol inducible system (bottom left). GFP (**C**) and Citrine-HA (**D**) served as negative controls. Total protein extracts were immunoblotted and detected with an anti-GFP (α-GFP) antibody. Protein expression analyses of constructs infiltrated are shown on right side with single GFP (**C**) and Citrine-HA (**D**) in the respective lower blot around 30 kDa. Cell death images were taken 2 days post-infiltration (**C**) or 3 days post-induction (**D**). Leaves are shown in false color, red indicates cell death and green healthy/alive tissue. WT = wild-type PML5; D362V = MHD mutant; K74R = P-loop mutant; GCC(2,3,4) A = N-myristoylation and S-acylation (PTM) mutant; GCC/2,3,4)A,D362V = PTM/MHD quadruple mutant. (**E**) Cell death phenotype in a single rosette leaf of 4-week-old Arabidopsis plant 18 h post-induction with 20 µM estradiol is shown. Leaves are shown in false color, red indicates cell death in wild-type PML5 and D362V lines, and green healthy/alive tissue in PML5 K74R and GFP negative control lines. (**F**) Growth restriction/cell death phenotype of two independent transgenic *Arabidopsis* lines conditionally overexpressing PML5-GFP (WT), D362V-GFP. A *35::GFP* plant line was used as a control. 10-day-old Arabidopsis seedlings grown on ½ MS plates supplemented with or without 20 µM estradiol are shown. Scale bars, 1 cm.

© The Author(s)

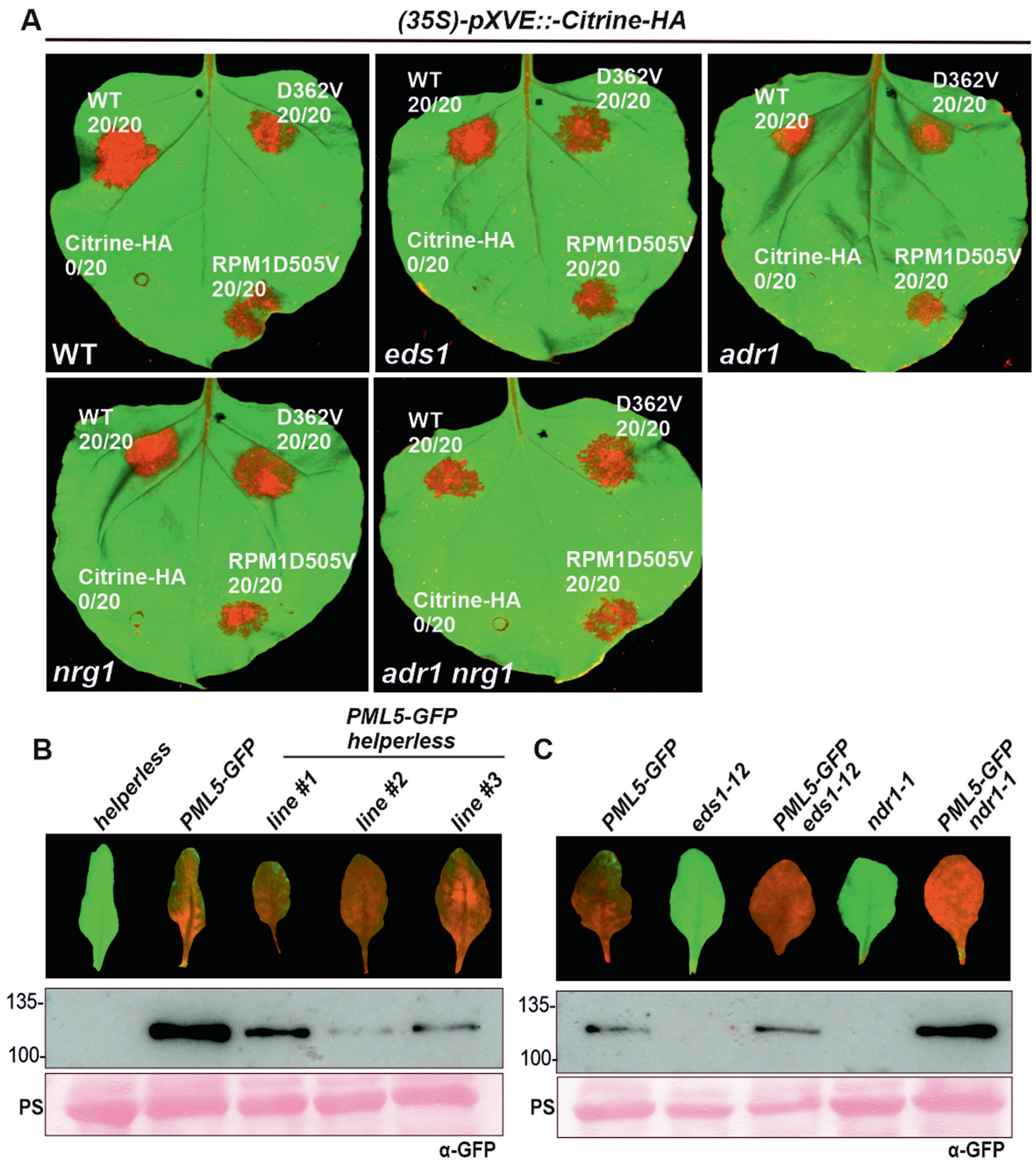
A
(35S)-pXVE::-Citrine-HA
WT
20/20
D362V
20/20
Citrine-HA
0/20
RPM1D505V
20/20
WT
eds1
adr1
nrg1
adr1 nrg1
B
helperless
PML5-GFP
PML5-GFP
helperless
line #1
line #2
line #3
135-
100-
PS
α-GFP
C
PML5-GFP
eds1-12
PML5-GFP
eds1-12
ndr1-1
PML5-GFP
ndr1-1
135-
100-
PS
α-GFP

Figure EV2. PML5-induced cell death is independent of EDS1, RNL helper NLRs, and NDR1.

(**A**) Cell death induced by transiently expressed wild-type PML5 (WT) and D362V mutant in WT, *eds1, adr1, nrg1, adr1 nrg1 N. benthamiana* mutant lines. The RPM1 D505V auto-active mutant was used as a positive control and Citrine-HA as a negative control. Images were taken 1 day post-induction with 20 μM estradiol. Leaves are shown in false color, red indicates cell death and green healthy/alive tissue. (**B**) Cell death in three independent transgenic *Arabidopsis helperless* mutant lines conditionally expressing PML5-GFP. PML5-GFP in Col-0 (PML5-GFP) served as the positive control. Protein blot (bottom) shows the expression of PML5-GFP detected with an anti-GFP (α-GFP) antibody in the positive control and three independent transgenic PML5-GFP *helperless* lines. Ponceau S (PS) staining is shown as a loading control. Images and samples for protein blot were taken 48 h post-induction with 20 μM estradiol. Leaves are shown in false color, red indicates cell death and green healthy/alive tissue. (**C**) Cell death in transgenic *Arabidopsis eds1-12* and *ndr1-1* mutant conditionally expressing PML5-GFP. PML5-GFP in Col-0 (PML5-GFP) served as the positive control. Protein blot (below) shows the expression of PML5-GFP detected with anti-GFP (α-GFP) antibody in the positive control and transgenic PML5-GFP *eds1-12* and *ndr1-1* mutants. Ponceau S (PS) staining is shown as a loading control. Samples for protein blot were taken 18 h post-induction (hpi) with 20 μM estradiol, cell death images were taken 48 hpi. Leaves are shown in false color, red indicates cell death and green healthy/alive tissue.

© The Author(s)

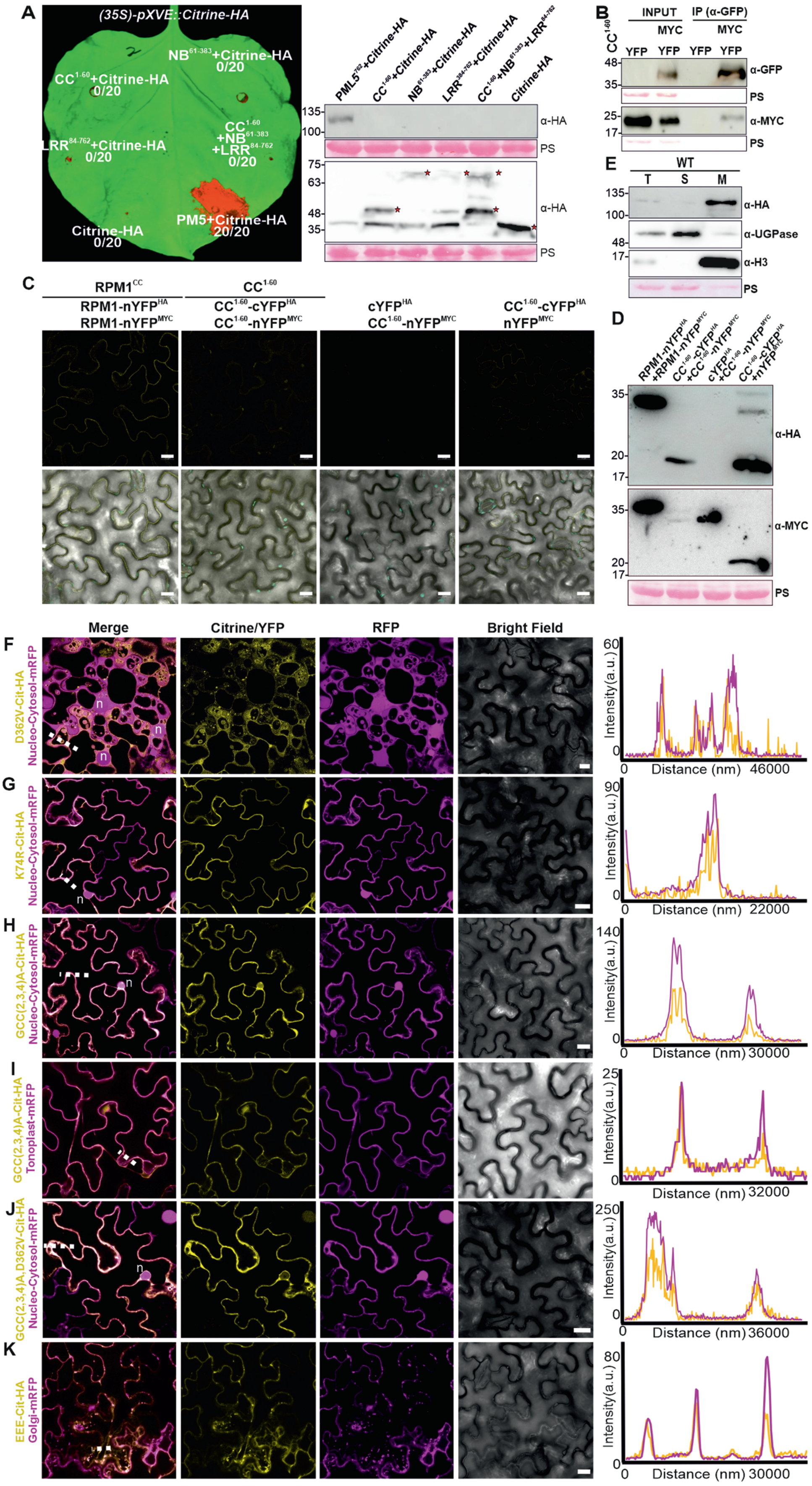
A
(35S)-pXVE::Citrine-HA
NB61-383+Citrine-HA 0/20
CC1-60+Citrine-HA 0/20
CC1-60 +NB61-383 +LRR84-762 0/20
LRR84-762+Citrine-HA 0/20
Citrine-HA 0/20
PM5+Citrine-HA 20/20
α-HA
PS
B
INPUT
IP (α-GFP)
MYC
YFP
α-GFP
α-MYC
E
WT
T
S
M
α-UGPase
α-H3
C
RPM1CC
RPM1-nYFPHA RPM1-nYFPMYC
CC1-60-cYFPHA CC1-60-nYFPMYC
cYFPHA CC1-60-nYFPMYC
CC1-60-cYFPHA nYFPMYC
D
Merge
Citrine/YFP
RFP
Bright Field
F
D362V-Cit-HA
Nucleo-Cytosol-mRFP
G
K74R-Cit-HA
H
GCC(2,3,4)A-Cit-HA
I
Tonoplast-mRFP
J
GCC(2,3,4)A,D362V-Cit-HA
K
EEE-Cit-HA
Golgi-mRFP
Intensity(a.u.)
Distance (nm)

Figure EV3. PML5 N-terminal 60 amino acids are sufficient for cell death induction.

(**A**) Combination of PML5 $CC^{1–60}$, $NB^{61–383}$, and $LRR^{384–762}$ domains is not sufficient to induce a WT-like cell death response. PML5 domains were transiently expressed alone or in co-infiltrations in *N. benthamiana*. Image was taken 2 days post-induction with 20 μM estradiol. (Right panel) Total protein extracts were immunoblotted and detected with an anti-HA (α-HA) antibody. Ponceau S (PS) staining is shown as a loading control. The red asterisks indicate the different fragments expressed. Leaves are shown in false color, red indicates cell death and green healthy/alive tissue. (**B**) PML5 $CC^{1–60}$ self-associates in *N. benthamiana* transient expression. C-terminally YFP and MYC tagged $CC^{1–60}$ were co-expressed. Total proteins were immunoprecipitated using anti-GFP (α-GFP) beads and immunoblotted using an anti-GFP (α-GFP) and anti-Myc (α-myc) antibody. Ponceau S (PS) staining is shown as a loading control. (**C**) Bimolecular fluorescence complementation (BiFC) experiment of PML5 CC^{1-60} fusion proteins, CC^{1-60}-$cYFP^{HA}$ and CC^{1-60}-$nYFP^{MYC}$, transiently expressed in *N. benthamiana*. No YFP complementation different to the negative control could be detected for the PML5 CC^{1-60} domain. RPM1 CC^{1-155} served as positive control showing YFP complementation. Co-expression of $cYFP^{HA}$ and $nYFP^{MYC}$ with PML5 CC^{1-60}-$nYFP^{MYC}$ and PML5 CC^{1-60}-$cYFP^{HA}$, respectively, served as negative controls. Scale bars, 20 μm. (**D**) Protein-blot analysis of total proteins from the transiently expressed proteins of the BiFC assay shown EV3C. Proteins were detected using anti-Myc (α-MYC) and anti-HA (α-HA) antibodies. Ponceau S (PS) staining is shown as a loading control. (**E**) Subcellular fractionation of transiently expressed PML5-Citrine-HA protein indicates a strong microsomal/membrane association. Total protein extracts were immunoblotted and detected with an anti-HA (α-HA) antibody for PML5, anti-UGPase (α-UGPase) as a cytosolic marker, and anti-Histone H3 (α-Histone H3) as a microsomal marker. T = total protein fraction; S = soluble protein fraction; M = microsomal protein fraction. (**F**–**K**) Confocal laser scanning microscopy showing subcellular localization of transiently expressed PML5-Citrine-HA mutants in comparison to different marker proteins: (**F**) D362V with nucleo-cytoplasmic mRFP; (**G**) K74R with nucleo-cytoplasmic mRFP; (**H**, **I**) GCC(2,3,4)A with nucleo-cytoplasmic mRFP and tonoplast mRFP; (**J**) GCC(2,3,4)A,D362V with nucleo-cytoplasmic mRFP; (**K**) T15E,L16E,F20E (EEE) with Golgi localized mRFP. White dotted lines indicate the area used for colocalization profile analysis and the corresponding profiles are shown on the right. *n* = nucleus. Scale bars, 10 μm; for information on compartment marker proteins, see the Methods section. Protein expression was induced with 20 μM estradiol and confocal imaging was performed at 6–8 h post-induction.

© The Author(s)

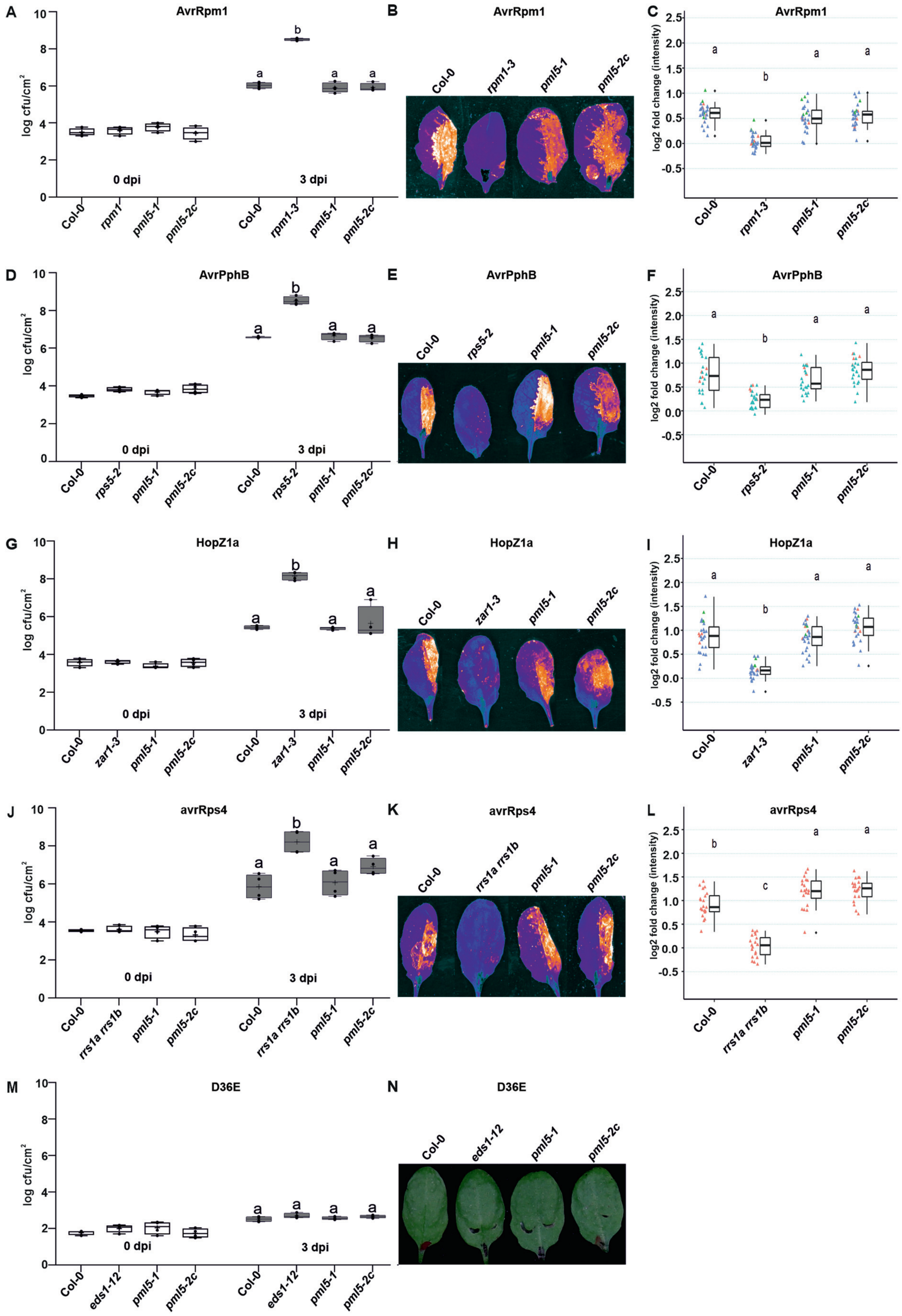
A
AvrRpm1
log cfu/cm^2
0 dpi
3 dpi
Col-0
rpm1
pml5-1
pml5-2c
rpm1-3
B
AvrRpm1
C
AvrRpm1
log2 fold change (intensity)
D
AvrPphB
rps5-2
E
AvrPphB
F
AvrPphB
G
HopZ1a
zar1-3
H
HopZ1a
I
HopZ1a
J
avrRps4
rrs1a rrs1b
K
avrRps4
L
avrRps4
M
D36E
eds1-12
N
D36E

 © The Author(s)

Figure EV4. *PML5* does not contribute to resistance against avirulent *Pst* DC3000.

(**A–M**) Resistance and cell death induced upon infection with different avirulent *Pst* DC3000 strains is not altered in *pml5* mutants. Rosette leaves of 6-week-old *Arabidopsis* plants were hand infiltrated with various *Pst* DC3000 strains using an $OD_{600} = 0.001$: (**A**) *Pst* DC3000 *AvrRpm1*, (**D**) *Pst* DC3000 *AvrPphB*, (**G**) *Pst* DC3000 *HopZ1a*, (**J**) *Pst* DC3000 *AvrRps4*, (**M**) *Pst* DC3000 D36E and bacterial growth was determined on day 0 and day 3 post infiltration. For (**A**, **D**, **G**, **J**, **M**), data are shown as boxplots and data points (colony forming units per square cm—cfu/cm^2) are indicated as black dots and represent 1 biological replicate with 4 technical replicates each ($n = 4$, with 6 leaf samples each). (**B**, **E**, **H**, **K**) Induction and (**C**, **F**, **I**, **L**) strength of cell death is not affected in *pml5* mutants. The right side of the leaves was hand infiltrated with *Pst* DC3000: *AvrRpm1* (**B**, **C**), *AvrPphB* (**E**, **F**), and *HopZ1a* (**H**, **I**) at an $OD_{600} = 0.1$ and with *Pf0*-1 *AvrRps4* (**K**, **L**) at an $OD_{600} = 0.2$. Infiltrated leaves were imaged with a Typhoon laser scanner 5 h post infiltration (hpi) for *AvrRpm1* (**B**), 22 hpi for *AvrPphB* (**E**), 24 hpi for *HopZ1a* (**H**), and *AvrRps4* (**K**). The leaf images are shown in false color: Purple/blueish parts indicate non-infiltrated healthy tissue, and orange/yellowish dead cells. (**C**, **F**, **I**, **L**) Quantification of cell death intensity measured of infiltrated leaves similar to leaves shown in (**B**, **E**, **H**, **K**). Details on the methodology can be found in Methods section. Data are presented as boxplots. Results shown are from 1 biological replicate with 20 technical replicates for (**L**), 2 biological replicates with 24 technical replicates for (**F**) and 3 biological replicates with 28 technical replicates for (**C**, **I**). Data points of the different biological replicates are indicated by differently colored triangles. (**N**) Infiltration of *Pst* DC3000 D36E does not induce visible disease symptoms on *pml5-1*, *pml5-2c* or Col-0 and *eds1-12*. Data information: For (**A**, **D**, **G**, **J**, **M**) data are presented as boxplots (center line, median; bounds of box, the first and the third quartiles; whiskers, 1.5 times the interquartile range; error bar, minima and maxima), similarly for (**C**, **F**, **I**, **L**). There, black dots additionally represent outliers. Data points with different letters indicate significant differences of $P \leq 0.05$ (one-way ANOVA with a post hoc Tukey's HSD test). Exact *P* values for all experiments are provided in Dataset EV1.

© The Author(s)

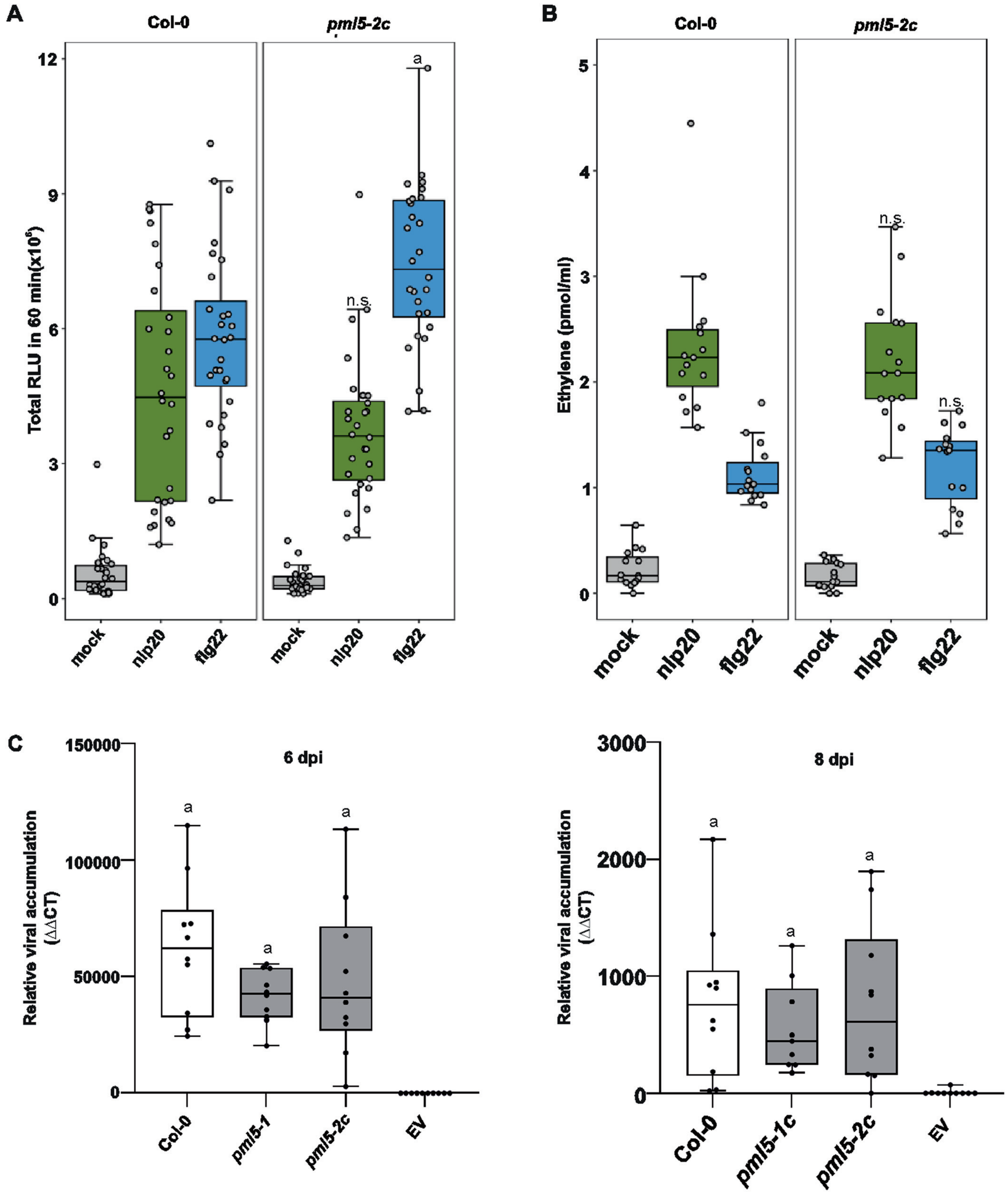
A
Col-0
pml5-2c
Total RLU in 60 min(x10⁶)
12
9
6
3
0
a
n.s.
mock
nlp20
flg22
mock
nlp20
flg22
B
Col-0
pml5-2c
Ethylene (pmol/ml)
5
4
3
2
1
0
n.s.
n.s.
mock
nlp20
flg22
mock
nlp20
flg22
C
6 dpi
Relative viral accumulation (ΔΔCT)
150000
100000
50000
0
a
a
a
Col-0
pml5-1
pml5-2c
EV
8 dpi
Relative viral accumulation (ΔΔCT)
3000
2000
1000
0
a
a
a
Col-0
pml5-1c
pml5-2c
EV

 © The Author(s)

Figure EV5. Loss of *PML5* has no effect on PRR-induced responses and TRV resistance.

(**A**) Total ROS production in leaf discs of Col-0 and *pml5-2c* mutant treated with water (mock), 1 µM nlp20, or 100 nM flg22 over 60 min. RLU, relative light unit. Data points are indicated as gray dots from four biological replicates each with seven technical replicates ($n = 28$) and plotted as boxplots. The gray color box indicates mock control, green indicates nlp20 treatment and blue indicates flg22 treatment in Col-0 and *pml5-2c* plants. (**B**) Ethylene accumulation in Col-0 and *pml5* mutant after 4 h treatment with water (mock), 1 µM nlp20, or 1 µM flg22. Data points are indicated as gray dots from five biological replicates each with 3 technical replicates ($n = 15$). (**C**) TRV viral accumulation was determined by qRT-PCR at 6 days post infection (dpi) (left) and 8 dpi (right) in infected Col-0, *pml5-1c*, and *pml5-2c* plants. Col-0 infected with an Empty Vector (EV) control served as negative control. Data points are from 10 different independent plants and the experiment was repeated 3 times (3 technical replicates) with similar results. Data information: Data in (**A**–**C**) are represented as boxplots (center line, median; bounds of box, the first and the third quartiles; whiskers, 1.5 times the interquartile range; error bar, minima and maxima). Statistical differences compared to the same treatment (**A**, **B**) in Col-0 were analyzed by two-sided Student's t-test ($\alpha = 0.05$) and are indicated with letters (a, $P < 0.01$, 'n.s.' not statistically different). In (**C**), data points with different letters indicate significant differences of $P \leq 0.05$ (one-way ANOVA with a post hoc Tukey's HSD test). Exact *P* values for all experiments are provided in Dataset EV1.
